# Systemic medications and dementia risk: a systematic umbrella review

**DOI:** 10.1038/s41380-025-03129-3

**Published:** 2025-07-24

**Authors:** Clara Belessiotis-Richards, Joseph Hayes, Ying Feng Yap, Shivangi Talwar, Michelle Eskinazi, Wenqianglong Li, Harry Ward, Pilar A. Letrondo, Madeleine Morelli-Batters, Andrea Bruun, Rongyu Lin, Talen Wright, Naaheed Mukadam

**Affiliations:** 1 Division of Psychiatry, University College London, London, United Kingdom; 2 North London NHS Foundation Trust, London, United Kingdom; 3 University College London Hospital NHS Trust, London, United Kingdom; 4 Kings’ College Hospital, Denmark Hill, London, United Kingdom; 5 Department of Psychology, Kingston University London, Kingston upon Thames, Surrey, United Kingdom; 6 Neural Injury Group, Nuffield Department of Clinical Neuroscience, John Radcliffe Hospital, University of Oxford, Oxford, United Kingdom; 7 Centre for Experimental Medicine and Rheumatology, Queen Mary University of London, London, United Kingdom; 8 Barts’ Health NHS Trust, The Royal London Hospital, London, United Kingdom; 9 Faculty of Health, Science, Social Care & Education, Kingston University London, Holmwood House, Grove Crescent, Kingston upon Thames, Surrey, United Kingdom; 10 Imperial College Healthcare NHS Trust, Hammersmith Hospital, London, United Kingdom

**Keywords:** Psychiatric disorders, Prognostic markers

## Abstract

**Background:**

Previous meta-analyses have found that systemic medications may modulate dementia risk. We aimed to provide an overview of this evidence to guide clinical practice and future research.

**Methods:**

We conducted an umbrella review of meta-analyses (PROSPERO CRD42021226307), searching databases from inception to 15th April 2024. Only peer-reviewed meta-analyses examining dementia risk and systemic medications in humans were included. Two authors independently screened studies for inclusion, extracted study data and assessed quality of meta-analyses using the AMSTAR-2 tool. Three authors independently rated the certainty of evidence for each drug using the GRADE framework.

**Results:**

68 meta-analyses were included, across 11 drug categories. Across meta-analyses, available data were primarily observational. Confounding by indication and potential reverse causality were important limitations. Randomised-controlled data were rare but supported an association between treatment of hypertension and reduced dementia incidence. Overall, we found moderate certainty evidence of reduced risk of dementia associated with anti-hypertensives, statins, sodium-glucose transport protein 2 (SGLT2) inhibitors, and glucagon-like peptide-1 receptor agonists (GLP-1 RAs), and moderate certainty of increased risk with anticholinergics.

**Discussion:**

Currently, there is insufficient evidence to advise repurposing any systemic drugs with the primary aim of reducing dementia risk. On the basis of our findings, we recommend proactive treatment of hypertension to reduce risk of all-cause dementia. Our findings did not find a difference between antihypertensive drug classes, but dementia risk was associated with blood pressure reading. In addition, we advise avoidance of anticholinergic drugs in cognitive impairment, with assessment of anticholinergic burden and consideration of alternatives during routine clinical contacts.

## Introduction

Worldwide, partly due to a welcome increase in longevity, the number of people with dementia is rapidly increasing: from an estimated 44 million in 2013 to an anticipated 135 million people by 2050 [1]. Unfortunately, no cure is available for dementia, leading to interest in modulation of risk of dementia, including through pharmacological agents and drug repurposing. Some High-Income Countries (HICs) have reported a decline in the age-adjusted incidence of dementia [2, 3]. This decline is thought to be due to reducing risk factors [4], leading to increasing interest in the prevention or delay of this condition [5–8]. The latest Lancet Commission review of dementia prevention suggested that interventions targeting 14 risk factors for dementia across the life-course could potentially delay or prevent up to 45% of dementia cases [4]. These include risk factors that are treatable through systemic medications, such as hypertension, diabetes, obesity, and depression [9]. Whether dementia risk can be modulated by systemic medications remains uncertain.

Previous studies have investigated the association between systemic medication use and dementia outcome by looking at treatment of risk factors for dementia (eg. hypertension [10]). There has also been recent interest in direct repurposing of systemic medications (eg. glucagon-like peptide-1 receptor agonists [11]). Identifying agents that may modulate dementia risk is crucial given the lack of readily available disease-modifying treatments for dementia and the increasing public health urgency of this condition.

The aim of this umbrella review was to synthesise current evidence regarding systemic medication use and dementia risk, to provide an objective evaluation of evidence gaps, and to identify priorities for future research or new drug trials. We aimed to provide a broad overview on multiple medication exposures [12] with the outcome of all-cause dementia risk.

## Methods

This was an umbrella review, presenting summary estimates from previously conducted individual meta-analyses. The review was registered in PROSPERO (CRD42021226307) [13] and is reported according to the PRIOR checklist [14] (Supplement 1).

### Search strategy

Searches were conducted across MEDLINE, AMED, PsycINFO, and Embase from inception to 1st June 2022 with no restrictions on language or publication dates. Searches were then re-run on 25th January 2023 and on 15th April 2024 to ensure up to date studies were included. Reference lists of included studies were also searched. We only included peer-reviewed publications. When clarification regarding data was needed, study authors were contacted.

The search strategy included terms for dementia and terms corresponding to the World Health Organisation Anatomical Therapeutic Chemical (ATC) classification [15] for medications (Supplement 2). The ATC classification system groups drugs by their mechanism of action according to five levels, primarily based on their pharmacological subgroup and the target organ or system. There are 14 main anatomical or pharmacological categories of the ATC, which we used to guide our search strategy.

First, we searched using the ATC classification. Search terms included, for example, “Antihistamine*”. “Antithrombotic*”, “Tacrolimus”, and “Opioid*”, separated by ‘OR’. We then searched for ‘dementia’, ‘cognitive impairment’, and ‘mild cognitive impairment’, separated by ‘OR’. We then combined the dementia search terms and the ATC search terms with Boolean operator ‘AND’. All searches were multi-purpose and applied to the following fields: abstract, title, subject headings, keyword heading name, drug trade name, candidate term words. Searches were limited to systematic reviews that included meta-analyses. The full search strategy is included in Supplement 2.

### Inclusion criteria

We included meta-analyses looking at systemic medications and risk of incident dementia in human participants of any age. Where systematic reviews used data from cohort studies, we only included results from the latest paper using that cohort data. We included individual patient data meta-analyses as we reasoned data from these would be informative although they do not follow the systematic review process.

68% of studies were independently screened by two reviewers during title and abstract selection, with the remaining 32% being screened by one author (YY). Two authors (CB-R or YY and NM) independently screened all potentially relevant full-text studies for inclusion.

### Exclusion criteria

We excluded studies looking at cognitive decline (in favour of a clinically applicable diagnosis of dementia), animal studies, and studies that were not peer-reviewed. We excluded analyses comparing subtypes of medications to each other as this was beyond the scope of our review. We excluded studies of topical medications.

### Quality assessment

For all included studies, two reviewers (any combination of CB-R, YY, ST, AB, AL, HW, PL, MM-B, RL, ME, TW, NM) independently extracted data using an agreed extraction proforma and quality-rated studies using the AMSTAR-2 tool [16]. Disagreements were settled through discussion with a senior author (NM). AMSTAR-2 is specifically designed for rating the quality of systematic reviews. It includes seven critical quality domains that cover protocol registration, search adequacy, justification of exclusions, risk of bias assessment, statistical methods, risk of bias in interpretation of results, and assessment of publication bias (Supplement 3). In our results, we reported the extent to which papers met individual critical quality criteria of AMSTAR-2 to allow for increased nuance in quality assessment.

### Data extraction

Data on number and type of studies, sample size, sex, mean age, follow-up, medication subtype, diagnostic criteria, summary effect estimates (random-effects models unless otherwise specified), heterogeneity (I^2^), and publication bias were extracted from each meta-analysis. The data extraction form was piloted and refined after discussion with senior authors (NM, JH).

Where overall sex and mean age were not reported in the systematic review these were extracted from individual studies and an overall figure was calculated. If studies presented data by study type and did not provide overall pooled estimates, we preferentially reported pooled estimates according to level of evidence (eg. randomised-controlled trial (RCT) instead of cohort data), if not otherwise stated. Where studies presented outcome data according to dementia type, we preferentially reported all-cause dementia outcome as this is more reflective of clinical practice, where mixed dementia is common. We preferentially reported adjusted measures of effect when these were available.

For each drug class, we examined overlap of individual studies by creating a citation matrix and calculating a corrected covered area [17–19] (Supplement 4). We did not conduct our own meta-analysis of extracted effect estimates because results would have overestimated the effect due to overlapping individual studies.

### Certainty of evidence

Three authors (CB-R, JH, NM) independently rated the certainty of evidence for each drug using the Grading of Recommendations, Assessment, Development, and Evaluations (GRADE) framework [20]. This framework describes four certainty levels: high, moderate, low, and very low. Observational data is automatically rated at low, while RCT data at high. Evidence is then downgraded based on any of the four following categories: risk of bias, imprecision, inconsistency, and publication bias. Evidence can be upgraded for the following reasons: magnitude of effect, dose-response gradient, and residual confounding decreasing or increasing magnitude of effect. Differences in ratings were resolved through discussion.

## Results

### Overview

**Fig. 1:**
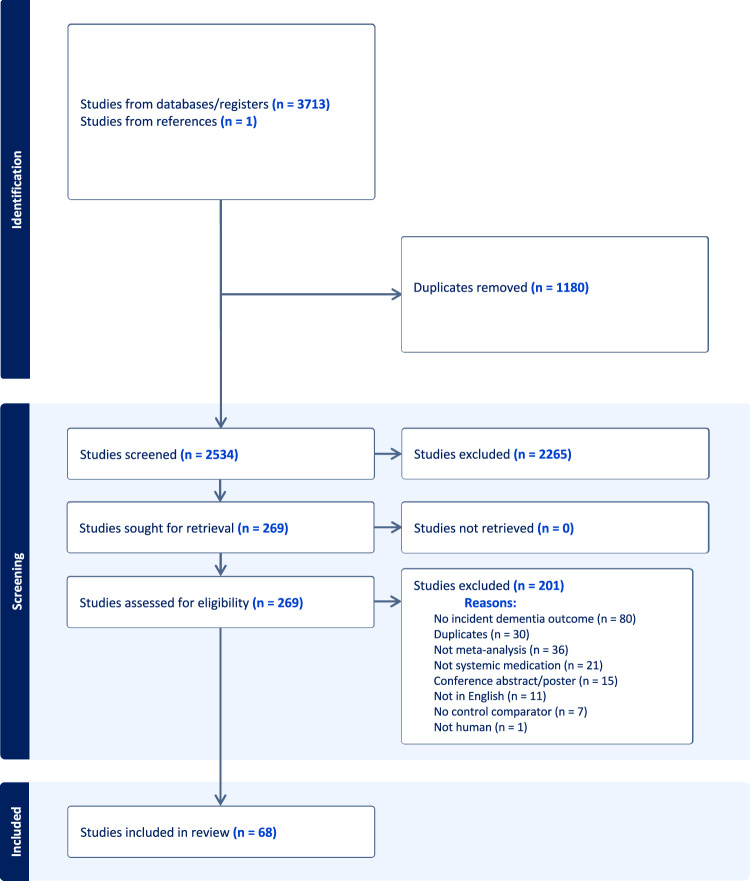
PRISMA flow diagram of searches and included studies.

Initial searches found 3444 articles, with 91 and 178 additional articles found in January 2023 and April 2024, respectively, yielding a total of 3713 papers for screening (Fig. 1). Following removal of duplicates, 2534 unique titles were screened, and 269 studies were sought for retrieval, of which 201 were excluded. The most common reason for exclusion was a lack of incident dementia as study outcome. Although we did not intend to exclude papers based on language, resource constraints meant we had to exclude 11 papers that were not in English.

In total, 68 meta-analyses were included in the final review, across 11 medication groups. The number of meta-analyses per medication group ranged from two to 18 and contained between two and 36 studies per meta-analysis (Table 1). The total number of participants in each meta-analysis ranged from 842–9,162,509. Participants’ mean age ranged from 59–78 years and between zero and 100% of participants were female. Follow-up periods ranged from 10.6 months to 32 years. Most meta-analyses included cohort and case-control studies, with limited RCT data available. Overall, six meta-analyses reported evidence of publication bias, across five medication classes. 56 out of 68 meta-analyses were published after 2014.

**Table 1:** Study details of meta-analyses included in the umbrella review.

Study	Study designs	Sample size in studies (range)	Female (%)	Mean age (years)	Follow up range (years)	Medication subtypes	Dementia diagnoses	Criteria used	Publication bias
**Anti-hypertensives**									
Adesuyan et al. [21]	3 Cohort	3962–6416	60	73	2.2–8.4	Any antihypertensive - subclasses angiotensin converting enzymes inhibitor (ACEI), angiotensin receptor II blocker (ARB), beta blocker (BB), calcium channel blocker (CCB)	AD	NINCDS-ADRDA, ICD-9, UK diagnostic codes, prescription of dementia medications, S. Korea diagnostic codes	Not reported
Chang-Quan et al. [30]	7 Cohort	302–6989	49	63	2.2 –32	Indapamide, nitrendipine +/- enalapril, perindopril and indapamide, telmisartan, hydrochlorothiazide, atenolol or reserpine, ACEi/BB/CCB/diuretics, CCB, unknown in 4 studies	Dementia, AD, VD	DSM-IV, NINCDS-ADRDA	No publication bias
Cunningham et al. [26]	4 RCTS	2418–4937	62	75	1.8–5	Diuretic, ARB, calcium based blocker based, beta blocker, isradipine methyldopa	Dementia	DSM ICD10 NINCDS-ADRDA	Not reported
Ding et al. [31]	6 Cohort	2379–8236	47	70	7–22	ACEi ARB BB CCB diuretic	Dementia	DSM-III/IV/V, NINCDS-ADRDA	Not reported
Hughes et al. [23]	7 RCTS	2902–11,140	51	71	4 (mean)	Diuretics, BB, ACEI, CCB, ARB	Dementia	DSM-IV, DSM-III-R, ICD-10, clinical	No publication bias
Hussain et al. [28]	7 Cohort, 2 open label extensions and 1 post-hoc analysis of a trial	1092–18,423	53	72	3–13	Dihydropyridine and non-dihydropyridine CCB class	Dementia	DSM-III-R, medical record, NINCDS-AIREN, NINCDS-ADRDA, DSM IV	No publication bias
Lennon et al. [38]	14 cohort studies	461–29335	58	73	4.3 (4.3) (mean and SD)	No data on class of antihypertensive	Dementia	DSM	Not reported
Levi-Marpillat et al. [33]	4 RCTs, 7 Cohort	1054–799,069	55	74	2–11	angiotensin-converting enzyme inhibitor; angiotensin receptor blocker II; beta-blocker; calciumchannel blocker; diuretic	VD, AD	DSM-III-R; ICD-10; NINCDS-ADRDA	No publication bias
McGuinness et al. 2009	3 RCTs	4736–5622	62	73	3 (mean)	calcium-channel blocker, diuretic, angiotensin receptor blocker	Dementia	DSM, ICD 10, NINCDS-ADRDA or acceptable equivalents	Not reported
Ou et al. [24]	12 Cohort	422–6537	57	76	2–32	ARBS, BBs, Diuretics, Loop diuretics, Potassium-sparing diuretics, thiazide diuretics, ACEIs, CCBs, Dihydropyridine, Nondihydropyridine	Dementia, AD	DSM, NINCDS-ADRDA, NINCDS-AIREN	No publication bias
Parsons et al. [32]	4 RCTs	2418–4937	62	75	2–4.5	Diuretic, DHP-CCB, BB	Dementia	Standardised diagnostic criteria but not specified	Not reported
Peters et al. [35]	2 RCTs, 2 Cohort	1092–3197	54	75	1–22.8	CCB, calcium channel blocker.	AD	DSM-III-R, ICD-10, NINCDS-ADRDA, INTERVIEW	Some publication bias
Peters et al. [36]	4 RCTs, 6 trials, 2 clinical trials treated as Cohort, 21 Cohort	275–6645	58	74	4.3 (median)	CCB, ACEi, ARB, diuretic, BB	Dementia	DSM-III-R or DSM-IV	No publication bias
Peters et al. [22]	5 RCTs	2822–11,008	47	69	2–11	Any antihypertensive	Dementia	DSM-IIIR or DSM-IV	Not reported
van Middelaar et al. [29]	9 RCTs	2902–11,140	48	71	2–9.8	Medications: chlorothalidon, nitrendipine, perindopril and indapamide, indapamide, telmisartan	AD, VD	Not stated	No publication bias
Xu et al. [25]	6 Cohort	302–6537	58	77	2.2–32	Not stated	Dementia, AD, VD	Not stated	No publication bias
Ye et al. [27]	7 Cohort	1107–819,491	44	73	3.2–8	ACEI, ARB, CACEI, NCACEI	AD	DSM-IV, Clinical, ICD-9, NINCDS-ADRDA, DSM-IIIR,	No publication bias
Zhuang et al. [37]	3 RCTs, 3 Cohort, 2 case-control	132–819491	49	74	2–6	ACEI, ARB, RASB	Dementia	DSM-IV, ICD-10, NINCDS-ADRDA, ICD-9, Read codes	No publication bias
**Statins**									
Chu et al. [42]	16 Cohort	123–2,004,692	33	59	2–25	simvastatin, atrovastatin, fluvastatin, pravastatin, rosuvastatin	Dementia, AD, VD	NINCDS-ADRDA, ICD-10, DS	No publication bias
Olmastroni et al. [40]	30 Cohort, 6 case-control	123–2,004,692	53	72	1–18	Hydrophilic vs lipophilic statins; High potency (Atorvastatin, Rosuvastatin) vs Low potency statins	Dementia, AD	NINCDS-ADRDA, ICD-10, DSM	Slight publication bias for dementia but not AD
Poly et al. [41]	23 Cohort, 7 case-control	974–4,500,000	54	72	1–18	Atorvastatin, Fluvastatin, Lovastatin, Pravastatin, Rosuvastatin, Simvastatin	Dementia, AD, VD	NINCDS-ADRDA, ICD-10, DSM	No publication bias
Song et al. [43]	8 Cohort	748–165,688	58	71	1–9	Not stated	Dementia, AD, VD	Case ascertainment using medical records	No publication bias
Wong et al. [45]	1 RCT, 10 Cohort, 1 nested case-control	1141–2,004,692	48	70	3–25	Atorvastatin, Fluvastatin, Lovastatin, Pravastatin, Rosuvastatin, Simvastatin	Dementia, AD	NINCDS-ADRDA, ICD-10, DSM	No publication bias for AD but for all-cause dementia larger studies reported results closer to null
Zhang et al. [44, 62]	22 Cohort, 9 case-control	548–1,290,071	54	72	0.8–18	Atorvastatin, Fluvastatin, Lovastatin, Pravastatin, Rosuvastatin, Simvastatin	Dementia, AD, VD, mixed dementia	Not stated	No publication bias
Zhou et al. [46]	4 Cohort, 2 case-control	845–5092	59	77	3–9	Simvastatin, atorvastatin, pravastatin	Dementia, AD	Not stated	Not reported
Antacids									
Desai et al. [51]	1 RCT, 5 Cohort	3076–73,679	50	76	1.5–11	Any PPI	Dementia	Not stated	Not reported
Hussain et al. [49]	8 Cohort, 4 case-control	148–353576	61	76	1.5–8.4	Not stated	Dementia, AD	ICD-10, DSM-IV, NINCDS-ADRDA	No publication bias
Li et al. [53]	6 Cohort	148–73,679	65	74	0.67–9	Any PPI	Dementia, AD	DSM-IV, diagnostic codes, ICD-9-CM	No publication bias
Song et al. 2019	5 Cohort, 4 case-control, 1 cross-sectional	3076–353,576	61	77	1.5–8.4	Not stated	Dementia, AD	Not stated	No publication bias
Virk et al. [52]	2 Cohort	148–694	70	71	5 (mean)	Any type of antacids - where studies reported separate ORs for use of any antacids and use of aluminum-containing antacids, the latter were selected for analysis	AD	NINCDS-ADRDA	No publication bias
Yoon et al. 2020	4 Cohort, 1 nested case-control	3076–73,679	62	74	1.5–8.4	Not stated	Dementia, AD	Not stated	No publication bias
Zhang et al. [48]	6 Cohort	148–73,679	61	74	3–8.4	dexlandsooprazole, esomeprazle, lanssooprazole, oomeprazole, pantoprazole, rabeprazoole	Dementia	ICD-9, ICD-10, diagnostic codes in medical records	No publication bias
**Medications for diabetes**									
Campbell et al. [58]	6 Cohort	365–145928	45	69	4–7	Metformin	Dementia, AD	ICD-9, ICD-10, clinical	Not reported
Kuate Defo et al. [61]	22 cohort studies, 5 case-control studies	123–1277250	41	68	0.5–16	Metformin, DPP4 inhibitors, insulin, thiazolidinediones, alpha glucosidase inhibitor, sulfonylurea, SGLT2i, sodium-glucose cotransporter-2 inhibitor, Pioglitazone, Rosiglitazone, GLP1RA, Meglitinide, Vildagliptin, Acarbose	Dementia	NINCDS-ADRDA, DSM, NINDS-AIREN, ICD	Some evidence of publication bias
McMillan et al. [55]	9 Cohort, 1 case-control, 1 cross-sectional	1109–377,838	47	70	2-12	Metformin, sulfonylurea, thiazolidinedione, Insulin, alpha glucosidase inhibitor	Dementia, AD, VD	ICD-9, DSM-IV, NINCDS–ADRDA, DSM-III	Not reported
Tang et al. [60]	7 cohort, 2 case-control	14,515–342,426	56	68	1.3–7.2	SGLT2 inhibitor, GLP-1RAs, DPP-4 inhibitors	Dementia, AD, VD	ICD-9, ICD-10	Not reported
Tian et al. [59]	19 cohort, 5 case-control studies	123–377838	44	70	2.0–15.0	Metformin, Sulfonylureas, Thiazolidinediones, Glucagon-like peptide-1 receptor agonists (GLP-1 RAs), Meglitinide, Sodium-glucose transport protein 2 (SGLT2) inhibitors, Alpha-glucosidase inhibitors, Dipeptidyl peptidase-4 (DPP-4) inhibitors, Insulin	Dementia, AD, VD	ICD‐9‐CM); ICD‐10; Diagnostic and Statistical Manual of Mental Disorders, 4th edition (DSM‐IV); DSM‐5	No publication bias
Ye et al. [57]	6 Cohort	365–445,179	57	70	3–11	Thiazolidinediones, metformin	Dementia, AD	ICD-9, ICD-10, algorithm, DSM-IV	No publication bias
Zhou et al. [56]	17 Cohort	1857–377,838	35	67	2-12	Metformin, sulfonylurea, thiazolidinediones, DPP4 inhibitors, alpha glucosidase inhibitor, benzoic acid derivatives, insulin, acarbose	Dementia	Not stated	No publication bias
**NSAIDs**									
deCraen et al. [66]	6 Cohort, 4 case-control	347–10,065	65	68	1–16	NSAID: Diclofenac + misoprostol, nimseulide, rofecoxib, naproxen	Dementia, AD	Not stated	No publication bias
Etminan et al. [63]	6 Cohort, 3 case-control	46–4915	59	71	2–15	exposure to, none stated	AD	Clinical investigation	No publication bias
Ji et al. [65]	8 Cohort	588–33,229	66	70	3.6–12	non-asprin NSAIDs and aspirin and all NSAID	Dementia, AD	Not stated	No publication bias
Szekely et al. [64]	3 case-control, 4 cross-sectional	100–6258	62	75	6.8 (median)	non-asprin NSAIDs and aspirin	AD	DSM, NINCDS-ADRDA	No publication bias
Veronese et al. [67]	5 Cohort	141–23,915	59	65	3–10	Aspirin	Dementia	DSM-III-R, DSM-IV, NINCDS-ADRDA, NINCDS-AIREN	No publication bias
Zhang et al. [44, 62]	10 Cohort	205–166,145	63	71	2–15	Aspirin, NSAID, acetaminophen	AD	Not stated	No publication bias
**Hormone replacement therapy**									
LeBlanc et al. 2001	2 Cohort, 10 case-control	84–1446	100	74	5–16	Different formulations: Oral HRT; conjugated equine estrogen (CEE); Use of any form for more than 6 month, Ever use, Ever use of any form, Current use, Current use of any form	Dementia, AD, VD	NINCDS-ADRDA	Some publication bias
Nerattini et al. [72]	4 RCTs, 24 case-control, 20 cohort, 1 cross sectional	84–4,696,633	100	72	4–18	HT, EPT, ET, MPA	Dementia, AD	Not stated	No publication bias
O’Brien et al. [68]	1 RCT, 5 Cohort, 5 case-control	227–7479	100	70	4–8	Duration (never, <6 months, ≥6 months, <1 year), Formulation (Estrogen; Estrogen and Progestogen)	Dementia, AD	DSM-III-R, DSM-IV, NINCDS-ADRDA	No publication bias
Wu et al. [69]	2 RCTs, 3 Cohort	2906–7233	100	70	4–18	Estrogen; Estrogen and Progestogen; Estradiol and Combination; Estrogen and combination; Estrogen, Progesterone and Combination; Hormone Therapy	Dementia, AD	Not stated	Publication bias among the studies reporting on AD but not for all-cause dementia
Yaffe et al. [71]	2 Cohort, 8 case-control	84–1446	100	75	Not reported	Estrogen replacement therapy; hormonal treatment (undefined);	Dementia, AD	NINCDS-ADRDA	Not reported
**Oncology treatments**									
Cui et al. [77]	11 Cohort	1314–196,684	0	74	2.7–10	Androgen Deprivation Therapy (ADT)	Dementia, AD	ICD-9; ATC; CPT; Read codes; clinical notes	No publication bias
Kim et al. [78]	7 Cohort	1314–30,703	0	74	2.3–5	androgen deprivation therapy (ADT)	Dementia, AD	Not stated	Not reported
Sari Motlagh et al. [76]	9 Cohort	1314–201,797	0	72	2.3–9.3	Androgen Deprivation Therapy (ADT)	Dementia, AD	ICD	Some publication bias
Zhang et al. [75]	8 Cohort	9117–1,238,879	0	74	2.3–9.3	All types of cancer treatment	Dementia, AD, VD	ICD, DSM, anti-dementia medication codes, NINCDS-ADRDA	No publication bias
Hinojosa-gonzalez et al. 2024	20 Cohort	1314–1,238,879	0	72	4–27	ADT	Dementia, AD	Not stated	Funnel plots reported but not interpreted
**Psychotropic medications**									
AlDawsari et al. [79]	35 observational studies	74–1,051,372	60	74	1.5–22	BZDs, Z drugs, BZDs and Z drugs combined, tricyclic antidepressants, SSRIs, SARIs, (lithium vs other antipsychotics), anticonvulsants	Dementia	Not stated	No publication bias
Lucchetta et al. [80]	5 Cohort, 7 case-control	273–417,172	52	76	6–22	BZDs	Dementia, AD	ATC, ICD-9, ICD-10, DSM-III, DSM-IV, DSM-5	No publication bias
Islam et al. [81]	2 Cohort, 6 case-control	150–9636	48	78	3–14.5	BZDs	Dementia	Not stated	Highly significant publication bias
Velosa et al. [83]	5 Cohort, 1 case-control	114–41,251	66	63	1.5–10	Lithium	Dementia, AD	ICD-8, ICD-9, ICD-10, DSM-IV	No publication bias
Wang et al. [82]	1 RCT, 3 Cohort, 2 case-control	716–141,740	74	75	2.7–8	anticholinergic ads, TCAs, MOAIs, SSRIs, heterocyclic ads	Dementia	Not stated	No publication bias
**Anticholinergics**									
Dmochowski et al. [84]	3 Cohort, 3 case-control	327–324,703	65	73	6–20	Overactive bladder treatments	Dementia	DSM‐III‐R, read codes for dementia diagnosis or anticholinesterase inhibitor prescription, ICD-9, ICD-10	No publication bias
Pieper et al. [85]	5 Cohort, 2 case-control	797–324,703	60	74	2–20	Not stated	Dementia	Unclear	Not reported
Zheng et al. [86]	11 Cohort, 3 case-control	109–790,240	57	73	1–20	antiparkinson, urological, antidepressant, cardiovascular, and gastrointestinal drugs	Dementia, AD, VD	DSM, ICD, clinical records, NINCDS-ADRDA	No publication bias
**Dietary supplementation**									
Wang et al. [88]	5 Cohort	633–4448	62	74	4–8	Vitamin E supplements	AD	NINCDS-ADRDA	No publication bias
Zhao et al. [87]	6 Cohort	587–7540	37	76	3–7	Vitamin E supplements	Dementia, AD, VD	Not specified	No publication bias
Zhou et al. [89]	5 Cohort	587–5269	31	77	5–10	Vitamin E supplements, Vitamin C supplements	Dementia, AD	NINCDS-ADRDA, DSM III & IV, ICD-10	No publication bias
**Anticoagulants**									
Moffitt et al. [91]	1 RCT, 3 Cohort	258–5221	50	77	2.7–3.4	Anticoagulant, subtypes not specified	Dementia	Clinical	No publication bias
Mongkhon et al. [90]	1 RCT, 4 Cohort	258–444,106	47	74	2.7–10	Warfarin, phenprocuomon, non vitamin K oral anticoagulants	Dementia	ICD-9, ICD-10	No publication bias

*AD* alzheimer’s dementia, *VD* vascular dementia, *Dementia* all-cause dementia, *NINCDS* national institute of neurological and communicative disorders and stroke, *ADRDA* the alzheimer’s disease and related disorders association, *ICD* international classification diseases, *DSM* diagnostic and statistical manual of mental disorders, *RCT* randomised controlled trial, *AIREN* association internationale pour la Recherche et l’Enseignement en Neurosciences, *ATC* anatomical therapeutic chemical, *CPT* current procedural terminology.

### Anti-hypertensives

18 meta-analyses [21–38] investigated the association between anti-hypertensive use and dementia risk (Table 1). Meta-analyses included between three and 12 studies with participant numbers ranging from 8072–831,674. Pooled effect estimates ranged from hazard ratio (HR) of 0.68 (95% confidence interval (CI) 0.28–0.99) to relative risk (RRs) of 0.94 (95% CI 0.90–0.99) for all anti-hypertensives (Table 2). I^2^ ranged from 0–70%. Of the three individual patient data (IPD) meta-analyses included, two were of cohort [31, 38] and one was of RCT data [22]. Out of a possible total of seven critical quality components from the AMSTAR-2, meta-analyses met between two and six of these. There was 5% of overlap of included studies between meta-analyses (Supplement 4).

**Table 2:** Results of systemic medication class and all-cause dementia risk, showing number of studies and total participantsper meta-analysis for analyses examining dementia risk and systemic medication use, ordered from highest to lowest quality.

Class of drug and study	N° of studies	N° of participants	Measure of effect (95% confidence interval)	I^2^ (%)	Quality rating^a^
**Anti-hypertensives**					
*Any or combination anti-hypertensive*					
Lennon et al. [38]^b^	17	34,519	HR^c^ = 0.68 (0.28–0.99)^d^	N/A	N/A
Peters et al. [22]^b^	5	28,008	OR = 0.87 (0.75–0.99)	N/A	N/A
Ding et al. [31]^b^	6	14,520	HR = 0.88 (0.79–0.98)	N/A	N/A
Cunningham et al. [26]	4	15,427	OR^e^ = 0.89 (0.72–1.09)	17	6
Hughes et al. [23]	7	41,719	OR = 0.87 (0.78–0.97)	0	6
Adesuyan et al. [21]	3	16,627	RR^f^ = 0.94 (0.90–0.99)	0	5
McGuinness et al. 2009	3	15,295	OR = 0.89 (0.69–1.16)	45	5
Parsons et al. [32]	4	15,427	RR = 0.90 (0.76–1.07)	Not reported	5
van Middelaar et al. [29]	9	57,682	RR = 0.93 (0.84–1.02)	16	5
Xu et al. [25]	6	22,518	RR = 0.86 (0.75–0.99)	41	5
Chang-Quan et al. [30]	7	21,665	RR = 0.86 (0.77–0.96)	0	4
Ou et al. [24]	12	37,553	RR = 0.79 (0.70–0.89)	68	4
Levi-Marpillat et al. [33]	11	831,674	HR^f^ = 0.91 (0.89–0.94)	70	2
*Calcium-channel blockers*					
Peters et al. [35]	4	9434	RR = 0.79 (0.53–1.17)	63	6
Hussain et al. [28]	10	75,239	RR = 0.70 (0.58–0.85)	88	5
Peters et al. [36]	11	11,936	OR = 0.92 (0.62–1.34)	42	5
Ding et al. [31]^b^	6	11,174	HR = 0.92 (0.75–1.14)	N/A	N/A
*Angiotensin-receptor blockers*					
Zhuang et al. [37]	5	42,512	RR = 0.79 (0.64–0.94)	92	6
Peters et al. [36]	7	9263	OR = 0.95 (0.56–1.61)	52	5
Ye et al. [27]	4	42,932	HR = 0.70 (0.50–0.96)	89	3
Ding et al. [31]^b^	3	5073	HR = 0.78 (0.50–1.22)	0	N/A
*ACE inhibitors*					
Zhuang et al. [37]	6	403,071	RR = 0.89 (0.82–0.96)	85	6
Peters et al. [36]	9	17,792	OR = 1.14 (0.90–1.44)	0	5
Ye et al. [27]	6	209,700	HR = 0.86 (0·75–0.98)	80	3
Ding et al. [31]^b^	6	11,112	HR = 1.03 (0.83–1.27)	0	N/A
*Diuretics*					
Parsons et al. [32]	2	8072	RR = 0.89 (0.72–1.09)	Not reported	5
Peters et al. [36]	12	16,508	OR = 0.84 (0.55–1.29)	68	5
Ding et al. [31]^b^	6	10,623	HR = 0.97 (0.76–1.24)	0	N/A
*Beta-blockers*					
Peters et al. [36]	10	13,207	OR = 1.17 (0.90–1.53)	19	5
Ding et al. [31]^b^	5	9826	HR = 0.96 (0.77–1.20)	0	N/A
**Statins**					
Song et al. [43]	8	753,197	RR = 0.62 (0.43–0.81)	73	7
Poly et al. [41]	30	9,162,509	RR = 0.83 (0.79–0.87)	62	7
Chu et al. [42]	16	2,745,149	RR = 0.85 (0.79–0.92)	50	7
Olmastroni et al. [40]	36	5,738,737	OR = 0.80 (0.75–0.86)	98	6
Wong et al. [45]	12	4,001,859	RR = 0.82 (0.69–0.97)	31	6
Zhang et al. [44, 62]	31	3,332,706	RR = 0.85 (0.80–0.89)	65	5
Zhou et al. [46]	6	13,770	RR = 0.77 (0.45–1.3)	Not reported	2
*Hydrophilic statins*					
Chu et al. [42]	3	3,297,350	RR = 0.88 (0.82–0.94)	Not reported	7
Poly et al. [41]	4	141,414	RR = 0.72 (0.63–0.82)	Not reported	7
Olmastroni et al. [40]	11	1,087,347	OR = 0.80 (0.71–0.89)	98	6
Wong et al. [45]	2	Not reported	RR = 1.07 (0.70–1.63)	48	6
*Lipophilic statins*					
Chu et al. [42]	3	3,297,350	RR = 0.74 (0.48–1.15)	Not reported	7
Poly et al. [41]	5	142,425	RR = 0.84 (0.75–0.94)	Not reported	7
Olmastroni et al. [40]	12	2,377,418	OR = 0.83 (0.76–0.90)	99	6
Wong et al. [45]	2	Not reported	RR = 0.94 (0.61–1.44)	0	6
**Antacids**					
Yoon et al. 2020	5	106,451	HR = 1.17 (0.91–1.49)	92	6
Zhang et al. [48]	6	166,146	HR = 1.29 (1.12–1.49)	61	6
Hussain et al. [49]	12	618,911	RR = 1.05 (0.96–1.15)	95	4
Li et al. [53]	6	106,599	RR = 1.23 (0.90–1.67)	95	4
Song et al. 2019	10	642,305	HR = 1.04 (0.92– 1.15)	96	4
Virk et al. [52]	2	842	RR = 0.83 (0.39–1.78)	0	3
Desai et al. [51]	6	178,191	HR = 1.16 (0.86–1.47)	93	2
**Medications for diabetes**					
*Any medication*					
Zhou et al. [56]	17	1,258,879	HR = 0.90 (0.83–0.99)	91	6
McMillan et al. [55]	11	703,918	RR = 1.01 (0.93–1.10)	92	5
Tian et al. [59]	24	1,683,415	OR = 0.80 (0.68–0.93)	Not reported	5
*Any oral medication*					
McMillan et al. [55]	3	392,421	RR = 0.95 (0.88–1.03)	83	5
Metformin					
Kuate Defo et al. [61]	15	2,187,059	RR = 0.83 (0.71–0.96)	99	7
Zhou et al. [56]	10	361,989	HR = 0.86 (0.74–1.00)	95	6
Campbell et al. [58]	6	354,766	HR = 0.76 (0.60–0.97)	63	5
McMillan et al. [55]	3	106,128	RR = 1.08 (0.49–2.36)	84	5
Tian et al. [59]	12	981,363	OR = 0.71 (0.46–1.09)	Not reported	5
Ye et al. [57]	6	288,772	RR = 0.79 (0.82–1.01)	63	2
*Thiazolidinediones*					
Kuate Defo et al. [61]	10	2,117,776	RR = 0.77 (0.59–1.00)	98	7
Zhou et al. [56]	7	265,416	HR = 0.81 (0.65–1.02)	75	6
McMillan et al. [55]	2	165,131	RR = 0.71 (0.55–0.93)	72	5
Tian et al. [59]	9	903,341	OR = 0.60 (0.51–0.69)	Not reported	5
Ye et al. [57]	3	516,612	RR = 0.75 (0.56–1.00)	0	2
*Sulfonylureas*					
Kuate Defo et al. [61]	11	2,136,275	RR = 1.39 (1.04–1.87)	100	7
Zhou et al. [56]	5	170,516	HR = 0.96 (0.82–1.11)	56	6
McMillan et al. [55]	2	30,813	RR = 0.96 (0.69–1.34)	54	5
Tian et al. [59]	8	818,758	OR = 1.43 (1.11–1.82)	Not reported	5
*Insulin*					
Kuate Defo et al. [61]	7	1,756,870	RR = 0.97 (0.78–1.20)	99	7
Zhou et al. [56]	3	452,586	HR = 1.39 (0.99–1.94)	79	6
McMillan et al. [55]	6	573,167	RR = 1.21 (1.06–1.39)	96	5
Tian et al. [59]	5	452,945	OR = 1.00 (0.72–1.39)	Not reported	5
*Dipeptidyl peptidase-4 (DPP-4) inhibitors*					
Kuate Defo et al. [61]	7	1,546,801	RR = 1.04 (0.79–1.38)	98	7
Zhou et al. [56]	2	131,243	HR = 0.65 (0.55–0.76)	57	6
Tian et al. [59]	4	839,888	OR = 0.78 (0.61–0.99)	Not reported	5
Tang et al. [60]	7	421,437	RR = 0.84 (0.74–0.94)	87	4
*Alpha-glucosidase inhibitors*					
Kuate Defo et al. [61]	5	168,869	RR = 1.04 (0.89–1.22)	61	7
Zhou et al. [56]	2	162,652	HR = 1.03 (0.87–1.21)	0	6
Tian et al. [59]	4	791,181	OR = 1.16 (0.92–1.47)	Not reported	5
*Benzoic acid derivatives*					
Zhou et al. [56]	2	162,652	HR = 1.05 (0.82–1.36)	48	6
*Sodium-glucose transport protein 2 (SGLT2) inhibitors*					
Kuate Defo et al. [61]	4	380,552	RR = 0.39 (0.20–0.76)	96	7
Tian et al. [59]	2	264,589	OR = 0.41 (0.22–0.76)	Not reported	5
Tang et al. [60]	3	114,475	RR = 0.62 (0.39–0.97)	83	4
*Glucagon-like peptide-1 receptor agonists (GLP-1 RAs)*					
Kuate Defo et al. [61]	3	177,535	RR = 0.35 (0.16–0.78)	99	7
Tian et al. [59]	2	124,180	OR = 0.34 (0.14–0.85)	Not reported	5
Tang et al. [60]	4	210,521	RR = 0.72 (0.54–0.97)	91	4
*Meglitinides*					
Kuate Defo et al. [61]	2	69,676	RR = 1.87 (1.43–2.45)	0	7
Tian et al. [59]	2	59,779	OR = 1.47 (0.90–2.42)	Not reported	5
**Non-steroidal anti-inflammatory drugs (NSAIDs)**					
*Any or combination NSAID*					
Etminan et al. [63]	9	19,569	RR = 0.72 (0.56–0.94)	49	7
deCraen et al. [66]	10	24,702	RR = 0.79 (0.68–0.92)	Not reported	6
Ji et al. [65]	8	54,575	RR = 0.99 (0.83–1.18)	51	6
Szekely et al. [64]	7	13,248	RR = 0.74 (0.62–0.89)	Not reported	6
Veronese et al. [67]	5	26,159	OR = 0.82 (0.55–1.22)	67	6
Zhang et al. [44, 62]	16	236,022	RR = 0.81 (0.70–0.94)	76	6
*Aspirin*					
Etminan et al. [63]	8	18,260	RR = 0.87 (0.70–1.07)	0	7
Ji et al. [65]	4	18,444	RR = 1.22 (1.02–1.45)	30	6
Zhang et al. [44, 62]	10	47,057	RR = 0.89 (0.70–1.13)	71	6
Veronese et al. [67]	5	26,159	OR = 0.82 (0.55–1.22)	67	6
*Nonaspirin*					
Ji et al. [65]	4	10,124	RR = 0.97 (0.70–1.35)	68	6
Zhang et al. [44, 62]	11	202,340	RR = 0.84 (0.58–1.23)	61	6
**Hormone replacement therapy**					
*Any medication*					
LeBlanc et al. 2001	12	5269	RR = 0.66 (0.53–0.82)	Not reported	4
Nerattini et al. [72]^g^	32	5,369,640	RR = 0.81 (0.70–0.94)	97	5
O’Brien et al. [68]	11	15,358	RR = 0.94 (0.71–1.26)	Not reported	3
Wu et al. [69]	5	18,773	OR = 1.16 (1.02–1.31)	20	3
Yaffe et al. [71]	10	3977	OR = 0.71 (0.53–0.96)	Not reported	2
*Oestrogen monotherapy*					
Nerattini et al. [72]^g^	18	1,422,346	RR = 0.85 (0.77–0.95)	84	5
Wu et al. [69]	10	416,199	OR = 1.09 (1.06–1.11)	73	3
*Oestrogen-progesterone*					
Nerattini et al. [72]^g^	11	1,481,300	RR = 0.91 (0.77–1.07)	96	5
Wu et al. [69]	2	177,673	OR = 1.16 (1.12–1.21)	37	3
**Oncology treatment**					
*Androgen depression therapy in prostate cancer*					
Hinojosa-Gonzalez et al. 2024	20	2,346,841	HR = 1.20 (1.11–1.29)	98	5
Kim et al. [78]	7	90,543	HR = 1.59 (1.16–2.18)	82	6
Cui et al. [77]	11	749,115	HR = 1.21 (1.13–1.30)	74	5
Zhang et al. [75]	8	1,535,436	RR = 1.18 (1.09–1.27)	90	5
Sari Motlagh et al. [76]	9	442,665	HR = 1.21 (1.11–1.33)	79	2
*Any chemotherapy*					
Zhang et al. [75]	5	3,666,529	RR = 0.77 (0.67–0.89)	81	5
*Chemotherapy for breast cancer*					
Zhang et al. [75]	2	74,642	RR = 0.83 (0.73–0.95)	48	5
*Endocrine therapy for breast cancer*					
Zhang et al. [75]	3	86,339	RR = 0.93 (0.82–1.06)	71	5
**Psychotropic medications**					
*Benzodiazepines*					
Lucchetta et al. [80]	12	980,860	OR = 1.38 (1.06–1.77)	98	7
AlDawsari et al. [79]	20	2,342,896	OR = 1.33 (1.19–1.49)	99	6
Islam et al. 2017	8	66,177	OR = 1.78 (1.33–2.38)	99	4
*Z-drugs*					
AlDawsari et al. [79]	8	954,852	OR = 1.43 (1.17–1.74)	98	6
*Antidepressants*					
AlDawsari et al. [79]	11	1,851,295	OR = 1.14 (0.88–1.46)	97	6
Wang et al. [82]	6	302,506	RR = 1.21 (1.12–1.29)	71	4
*Antipsychotics*					
AlDawsari et al. [79]	5	1,177,342	OR = 0.97 (0.68–1.39)	91	6
*Anticonvulsants*					
AlDawsari et al. [79]	2	46,787	OR = 0.98 (0.85–1.13)	0	6
*Lithium*					
Velosa et al. [83]	5	49,979	OR = 0.51 (0.36–0.72)	92	5
*Anticholinergics*					
Pieper et al. [85]	7	506,045	OR = 1.20 (1.09–1.32)	86	5
Dmochowski et al. [84]	6	645,865	RR = 1.46 (1.17–1.81)	97	4
Zheng et al. [86]	14	1,564,181	RR = 1.20 (1.15–1.26)	83	4
**Dietary supplementation**					
*Vitamin E*					
Zhou et al. [89]	5	14,427	RR = 0.80 (0.70–0.92)	0	5
Wang et al. [88]	5	13,311	RR = 0.81 (0.50–1.33)	69	5
Zhao et al. [87]	6	24,092	OR = 0.83 (0.73–0.94)	0	4
*Vitamin C*					
Zhou et al. [89]	4	10,798	RR = 0.81 (0.70–0.93)	0	5
**Anticoagulants**					
*Any medication*					
Moffitt et al. [91]	4	7063	OR = 0.89 (0.47–1.69)	66	6
Mongkhon et al. [90]	5	448,418	RR = 0.79 (0.67–0.93)	60	6
*Vitamin K antagonist*					
Mongkhon et al. [90]	5	435,429	RR = 0.77 (0.66–0.90)	48	6

^a^Number of critical quality components met (max = 7).

^b^Individual patient data meta-analysis.

^c^Hazard Ratio.

^d^Converted to reflect the HR for treated hypertension compared to untreated hypertension.

^e^Odds Ratio.

^f^Relative Risk.

^g^Observational data reported instead of randomised-controlled trial data due to only one study being included.

Six meta-analyses [22, 23, 26, 29, 31, 34] reported analyses of RCT data exclusively (Table 1). Pooled estimates from these studies ranged from odds ratio (OR) 0.87 (95% CI 0.78–0.97) to RR 0.93 (95% CI 0.84–1.02). Four out of six pooled effect estimates had 95% CIs crossing the null value (Table 2).

Meta-analyses examining subtypes of anti-hypertensives found no association between dementia incidence and diuretics [31, 32, 36] and inconsistent results for beta-blockers [31, 36] and ACE-inhibitors [27, 31, 36, 37]. There was a tendency towards a possible protective effect of calcium-channel blockers [28, 31, 35, 36] and angiotensin-receptor blockers [27, 31, 36, 37] but some 95% CIs contained the null value, although these had smaller numbers of participants (Table 2). Ding et al. [31] found no evidence for superiority of any specific anti-hypertensive drug subtype and dementia incidence.

Lennon et al. [38], carried out an IPD meta-analysis and, using a fully adjusted model, found a protective effect of any anti-hypertensive treatment when compared to untreated hypertension (HR 0.68, 95% CI 0.28–0.99). Ding et al. [31] in another IPD of cohort studies stratified by blood pressure readings found an association between use of antihypertensive medication and reduced dementia incidence compared to non-use among individuals in the highest blood pressure group (HR 0.88, 95% CI 0.79–0.98). This association was not sustained in the normal blood pressure stratum. In addition, the IPD meta-analysis by Peters et al. [22] of five RCTs found the association between antihypertensive use and reduced dementia incidence was strongest amongst individuals treated to a systolic blood pressure (SBP) of <147 mmHg – consistent with guidelines [39] – and bigger effects on dementia were seen with larger reductions in SBP.

Using the GRADE framework, we judged there was moderate certainty of evidence for a reduced risk of dementia with anti-hypertensives (Table 3).

**Table 3:**
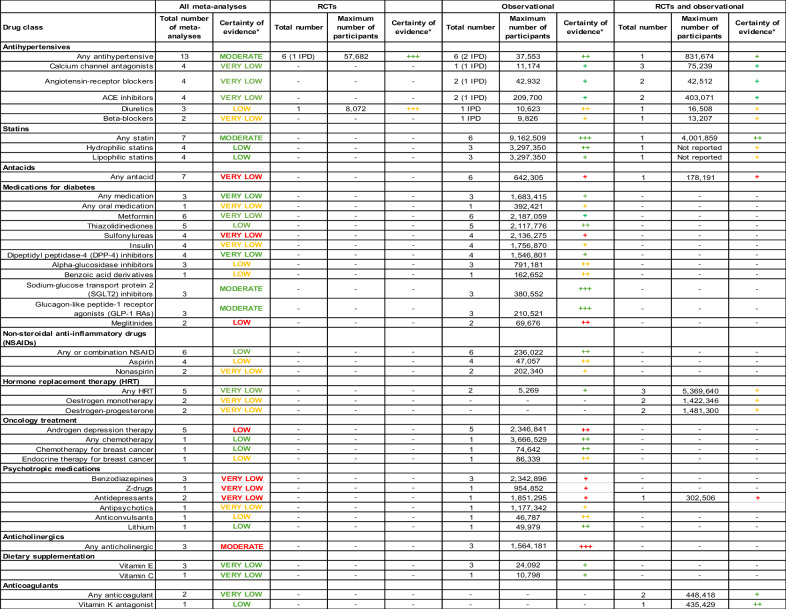
Detailed GRADE ratings for each drug type by evidence type.

^*^+++ Strong evidence for effect, ++ partial evidence of effect, + weak evidence of effect. Green = protective effect, Amber = no effect, Red = harmful effect.

### Statins

Seven meta-analyses [40–46] investigated the association between statin use and dementia risk (Table 1). Meta-analyses included between six and 36 studies with participant numbers ranging from 13,770–9,162,509. Pooled RR estimates ranged from 0.62 (95% CI 0.43–0.81) to 0.85 (95% CI 0.80–0.89) (Table 2). Heterogeneity ranged from 31–98%. Number of critical quality components met ranged from two to seven. There was 27% of overlap of studies between meta-analyses (Supplement 4).

Wong et al. [45] was the only meta-analysis to include an RCT investigating incidence of dementia among 20,536 participants [47]. They found no association between simvastatin use and dementia risk (OR 1.00, 95% CI 0.61–1.65) (Table 2). Meta-analyses reported high levels of bias within individual studies, and most were limited to cohort studies.

There was evidence of reduced incidence of all-cause dementia among hydrophilic statins in three meta-analyses (RR 0.72, 95% CI 0.63–0.82, to RR 0.88, 95% CI 0.82–0.94), though Wong et al. [45] found no association (RR 1.07, 95% CI 0.70–1.63). The evidence for lipophilic statins was weaker, with 95% CIs for effect estimates in two out of four meta-analyses including the null value, and upper limits of 95% CIs in the remaining two approaching the null (Table 2).

There was a possible dose-response gradient between statin use and risk of dementia in one meta-analysis [44], showing that risk of dementia reduced by 20% per one year of statins use (RR: 0.80; 95% CI, 0.73–0.87), and with increasing doses of statins (RR: 0.89; 95% CI, 0.83–0.96). GRADE was upgraded to a moderate certainty of a reduced risk of dementia (Table 3).

### Antacids

Seven meta-analyses [43, 48–53] investigated the association between antacid use and dementia risk (Table 1). Meta-analyses included between two and 12 studies with participant numbers ranging from 842–642,305. Pooled estimates ranged from RR of 0.83 (95% CI 0.39–1.78) to HR of 1.29 (95% CI 1.12–1.49) (Table 2). Most meta-analyses had high heterogeneity levels, ranging from 92–96%, apart from one meta-analysis [52] with low (0%) and one meta-analysis [48] with moderate heterogeneity (61%). Number of critical quality components met ranged from two to six. There was 32% of overlap of studies between meta-analyses (Supplement 4).

Desai et al. [51] was the only meta-analysis to include RCT data, from one study [54]. They found no evidence for an association between dementia and pantoprazole use (HR 1.20, 95% CI 0.81–1.78) (Table 2). GRADE rating was of very low certainty of an increased risk of dementia with antacid use (Table 3).

### Medications for diabetes

Seven meta-analyses [55–61] investigated the association between medications for diabetes and dementia risk (Table 1). Meta-analyses included between six and 27 studies with participant numbers ranging from 30,813–2,136,275. These meta-analysed results compared users of diabetes medications with users of other diabetes medications or people without diabetes. Number of critical quality components met ranged from two to seven. There was 21% of overlap of studies between meta-analyses (Supplement 4).

Pooled effect estimates for any diabetes treatment ranged from HR 0.90 (95% CI, 0.83–0.99) to RR 1.01 (95% CI 0.93–1.10) across three reviews [55, 56, 59] (Table 2). I^2^ was high (91–92%). RCTs were available for cognitive performance, not incident dementia [58], so were not included in the meta-analyses.

Three recent meta-analyses [59–61] found reduced incidence of dementia associated with glucagon-like peptide-1 receptor agonists (GLP-1 RAs) and sodium-glucose transport protein 2 (SGLT2) inhibitors. Pooled effect estimates ranged from OR 0.34 (95% CI 0.14–0.85) to RR 0.72 (95% CI 0.54–0.97) for GLP-1 RAs, and RRs ranged from 0.39 (95% CI 0.20–0.76) to 0.62 (95% CI 0.39–0.97) for SGLT2 inhibitors (Table 2).

Results tended towards reduced risk of incident dementia with thiazolidinediones (effect estimates 0.71–0.81), but three out of five 95% CIs contained the null value. There was some evidence of possible reduced incidence of dementia with DPP4-inhibitors (HR 0.65, 95% CI 0.55–0.76 to RR 0.85, 95% CI 0.74–0.94), though this was not reflected in the most recent meta-analysis [61] (RR 1.04, 95% CI 0.79–1.38) (Table 2). There was no association between dementia and acarbose or benzoic acid derivatives [56] or alpha-glucosidase inhibitors use, and there were inconsistent results for sulfonylurea and insulin use.

More recent meta-analyses [59, 61] suggested a possible increased risk with sulfonylurea and reduced risk with insulin use. These meta-analyses also showed a tendency towards increased risk of dementia with meglitinide use, though confidence intervals contained the null for one meta-analysis and numbers were small. Results were inconsistent for metformin, with a tendency towards reduced dementia risk though most pooled effect estimates contained the null in their 95% CI. The most recent meta-analysis [61], which was very large and of high quality, found reduced dementia incidence with metformin use (RR 0.83, 95% CI 0.71–0.96).

Overall, our GRADE assessment was of very low certainty of a protective effect for all diabetes medications (Table 3). However, we judged certainty of reduced risk of dementia to be moderate for GLP-1 RAs and SGLT2 inhibitors due to magnitude of effect. There was low and very low certainty of increased risk of dementia associated with meglitinides and sulfonylureas, respectively.

### Non-steroidal anti-inflammatory drugs (NSAIDs)

Six meta-analyses [62–67] investigated the association between NSAID use and dementia risk, only including observational data (Table 1). Meta-analyses included between five and 16 studies with participant numbers ranging from 13,248–236,022. Pooled RRs ranged from 0.74 (95% CI 0.62–0.89) to 0.99 (95% CI 0.83–1.18) (Table 2). Four out of six [62–64, 66] estimates reported CIs excluding the null value. This putative protective effect seemed more pronounced when analyses were restricted to studies where NSAID use was prolonged for two or more years (HR 0.42, 95% CI 0.26–0.66) [64].

I^2^ ranged from 49–76%. Number of critical quality components met ranged from six to seven. There was 19% of overlap of studies between meta-analyses (Supplement 4).

Studies looking at aspirin use generally [67] found no clear association with dementia incidence. This was also the case when subgroup analyses were carried out for non-aspirin NSAIDs (Table 2).

Overall, we judged there to be low certainty of evidence of a reduced risk of dementia with NSAIDs use (Table 3).

### Hormone replacement therapy (HRT)

Five meta-analyses [68–71] investigated the association between HRT and dementia risk. Meta-analyses included between five and 12 studies with participant numbers ranging from 3977–5,369,640. Pooled effect estimates ranged from RR 0.66 (95% CI 0.53–0.82) to OR 1.16 (95% CI 1.02–1.31) (Table 2). Heterogeneity was only reported in two studies [69, 72] (20–97%). Number of critical quality components met ranged from two to five. There was 16% of overlap of studies between meta-analyses (Supplement 4).

Studies [70, 71] before 2014 based on case-control designs suggested a possible protective effect of any HRT on dementia (RR 0.66, 95% CI 0.53–0.82 and OR 0.71, 95% CI 0.53–0.96) (Table 2). More recent studies have found evidence of increased risk of dementia with HRT using RCT data. In particular, data from the Women’s Health Initiative Memory Study (WHIMS) [73], a RCT examining 7479 women aged 65–79 years at baseline, reported in three meta-analyses [68, 69, 72], found an overall increased risk of dementia with any HRT use (HR 1.76, 95% CI 1.19–2.60) over 4.5 years follow-up compared to placebo.

Two meta-analyses [69, 72] investigated the effect of oestrogen-progesterone compared to oestrogen monotherapy. Wu et al. [69] found a positive association for both oestrogen-progesterone (OR 1.16, 95% CI 1.12–1.21) and oestrogen monotherapy (OR 1.09, 95% CI 1.06–1.11) with dementia (Table 2). A more recent and much larger meta-analysis by Nerattini et al. [72] found a slightly protective effect of oestrogen monotherapy on risk of dementia (RR 0.85, 95% CI 0.77–0.95), that was not sustained in the oestrogen-progesterone group (RR 0.91, 95% CI 0.77–1.07) in observational data. This is in keeping with the WHIMS RCT [73], which found an increased risk of dementia in the oestrogen-progesterone group (HR 2.05, 95% CI 1.21–3.48) at 4.5 years follow-up, but not with oestrogen monotherapy (HR 1.49, 95% CI 0.83–2.66). However, a recent meta-analysis by Nerattini et al. [72] included two long-term follow-up studies of the WHIMS, at 13 [74] and 18 years [74], the latter of which found no association with dementia for oestrogen-only (RR 1.02, 95% CI 0.73–1.43) or oestrogen with progesterone replacement therapy (RR 1.32, 95% CI 0.98–1.77).

Overall, we judged there to be very low certainty of evidence of a reduced risk of dementia with HRT use (Table 3).

### Oncology treatments

Five meta-analyses [75–78] investigated the association between androgen depression therapy (ADT) for prostate cancer and dementia risk. Meta-analyses included between seven and 20 studies with participant numbers ranging from 90,543–2,346,841. Pooled effect estimates ranged from RR 1.18 (95% CI 1.09–1.27) to HR 1.59 (95% CI 1.16–2.18) (Table 2). I^2^ was between 74–98%. Number of critical quality components met ranged from two to six. There was 29% of overlap of studies between meta-analyses (Supplement 4).

Zhang et al’s meta-analysis [75] also looked at any chemotherapy use for all cancer patients (RR 0.77, 95% CI 0.67–0.89) in five studies (*n* = 3,666,529), chemotherapy for breast cancer (RR 0.83, 95% CI 0.73–0.95) in two studies (*n* = 74,642), and endocrine therapy for breast cancer (RR 0.93, 95% CI 0.82–1.06) in three studies (*n* = 86,339) and dementia risk (Table 2).

Our GRADE assessment found low certainty of an increased risk of dementia with ADT, and low certainty of a reduced risk of dementia with chemotherapy (Table 3).

### Psychotropic medications

Three meta-analyses [79–81] investigated the association between benzodiazepine use and dementia risk. Meta-analyses included between eight and 20 studies with participant numbers ranging from 66,177–2,342,896. Pooled ORs ranged from 1.33 (95% CI 1.19–1.49) to 1.78 (95% CI 1.33–2.38) (Table 2). I^2^ was high (98–99%). Number of critical quality components met ranged from four to seven. There was 30% of overlap of studies between meta-analyses (Supplement 4).

Two meta-analyses, by AlDawsari [79] and Wang [82], investigated the association between antidepressant use and dementia risk. AlDawsari et al. included 11 studies (*n* = 1,851,295) and Wang et al. six studies (*n* = 302,506). Pooled effect estimates were OR of 1.14 (95% CI 0.88–1.46) [79] and RR of 1.21 (1.12–1.29) [82], respectively (Table 2). I^2^ was high (97%) [79] and moderate (71%) [82], respectively. Number of critical quality components met were six [79] and four [82], respectively. There was no overlap of studies between meta-analyses (Supplement 4).

AlDawsari et al. also found no association between dementia risk and anticonvulsant use (OR 0.98, 95% CI 0.85–1.13) in two studies (*n* = 46,787), and antipsychotic use (OR 0.97, 95% CI 0.68–1.39) in five studies (*n* = 1,177,342) (Table 2). The same authors found a possible increased risk of dementia associated with Z-hypnotic drugs (OR 1.43, 95% CI 1.17–1.74) in a meta-analysis of eight studies (*n* = 954,852).

Velosa et al. carried out a meta-analysis [83] of five studies (*n* = 49,979), finding evidence of a possible reduced risk of dementia with lithium use (OR 0.51, 95% CI 0.36–0.72) (Table 2). This review met five critical quality components.

Our GRADE assessment found very low certainty of an increased risk of dementia with benzodiazepines, antidepressants, and Z-drugs, and low certainty of a reduced risk of dementia with lithium (Table 3).

### Anticholinergics

Three meta-analyses [84–86] investigated the association between anticholinergics and dementia risk. Meta-analyses included between six and 14 studies with participant numbers ranging from 506,045–1,564,181. Pooled RRs ranged from 1.20 (95% CI 1.15–1.26) to 1.46 (95% CI 1.17–1.81) (Table 2). I^2^ was between 83–97%. Number of critical quality components met ranged from four to five. There was 21% of overlap of studies between meta-analyses (Supplement 4).

There was evidence of a possible dose-dependent relationship, with subgroup analyses in the meta-analysis by Pieper et al. [85] finding a stronger association between anticholinergic use for at least one year and dementia incidence (RR 1.50, 95% CI 1.22–1.85) than for shorter durations of use (RR 1.20, 95% CI 1.09–1.32) (Table 2). There was a lack of data on subtypes of anticholinergics.

Our GRADE assessment upgraded this evidence to moderate certainty of increased risk of dementia with anticholinergics due to a dose-response relationship (Table 3).

### Dietary supplementation

Three meta-analyses investigated vitamin E supplementation [87–89] and dementia risk (Table 2). Meta-analyses included between five and six studies with participant numbers ranging from 587–7540. Pooled effect estimates ranged from RR 0.80 (95% CI 0.70–0.92; I^2^ 0%) to OR 0.83 (95% CI 0.73–0.94; I^2^ 69%) (Table 2). Number of critical quality components met ranged from four to five. There was 38% of overlap of studies between meta-analyses (Supplement 4). All data were observational.

One meta-analysis investigated vitamin C supplementation [89] and dementia risk (RR 0.81, 95% CI 0.70–0.93) across four studies (*n* = 10,798) (Table 2). This review met five critical quality components.

Our GRADE assessment found there was very low certainty of a reduced risk of dementia with vitamin E and C (Table 3).

### Anticoagulants

Two meta-analyses [90, 91] investigated the association between any anticoagulants and dementia risk. Mongkhon et al. [90] included five studies (*n* = 448,418) and Moffitt et al. [91] four studies (*n* = 7063), with pooled effect estimates of RR of 0.79 (95% CI 0.67- 0.93) and OR 0.89 (95% CI 0.47, 1.69), respectively (Table 2). I^2^ was moderate in both (60–66%). Number of critical quality components met were six for both. There was a high degree of overlap (28%) (Supplement 4).

One RCT [92] was included in both meta-analyses, however the outcome of interest was cognitive performance, not incident dementia. Mongkhon et al. found a protective effect of warfarin when compared to no anticoagulant use (RR 0.77, 95% CI 0.66–0.90) (Table 2).

Our GRADE assessment found there was very low certainty of a reduced risk of dementia with anticoagulation, and low certainty for vitamin K antagonists specifically (Table 3).

## Discussion

To our knowledge, this is the first umbrella review investigating the association between systemic medications and dementia risk. Overall, we found 68 meta-analyses across 11 drug classes. The most studied drug class was anti-hypertensive medications (*n* = 18) and the least studied were anticoagulants (*n* = 2). Most meta-analyses examined risk of all-cause dementia and we preferentially reported these results, though some focussed on Alzheimer’s disease (AD) specifically (Table 1).

Using the GRADE framework (Table 3), we found moderate certainty evidence of a reduced risk of dementia associated with anti-hypertensives, statins, sodium-glucose transport protein 2 (SGLT2) inhibitors, and glucagon-like peptide-1 receptor agonists (GLP-1 RAs), and moderate certainty of an increased risk of dementia with anticholinergics.

The strongest evidence we found supported the use of anti-hypertensive medications in reducing dementia risk, reflected in both RCT and observational studies (Table 2). This reinforces the need for effective control of vascular risk factors in dementia prevention. Effects on dementia incidence appeared associated with their effect on blood pressure rather than drug subtype and this effect seemed to be sustained across a range of settings and populations [38]. Though a Cochrane review of RCTs of anti-hypertensives [26] concluded there was insufficient evidence to advise their use for primary dementia prevention, a more recent individual patient data meta-analysis by Peters et al. [22] of RCTs found reduced incidence of dementia in treated hypertension. However, RCTs for anti-hypertensive treatments primarily examined cardiovascular events (eg. Stroke), mortality, and cognitive testing as primary outcomes, with dementia included as a secondary outcome.

In our umbrella review, there was limited RCT evidence available for other systemic medications (Table 3). Even when RCT evidence was available, dementia was a secondary outcome. An exception to this was the WHIMS RCT, for which dementia was a primary outcome. Results of the WHIMS RCT were included in several meta-analyses, which found evidence that oestrogen-progesterone HRT [74] and antidepressants [93] increased the risk of all-cause dementia. In contrast to RCT data, in our umbrella review, observational studies found a protective effect of oestrogen monotherapy HRT on dementia risk. Results from the WHIMS RCT were also included in one of our included meta-analyses on antidepressant use and dementia [82], which found an increased risk of dementia with antidepressant use among postmenopausal women.

Our umbrella review found that RCTs suggested moderate evidence of a reduced risk of dementia with statin use. The included RCTs all examined dementia as a secondary outcome, with primary endpoints being cardiovascular outcomes, such as stroke. However, a Cochrane review of the RCT evidence for statins [94] and an RCT examining the role of statins in slowing progression of AD [95] have found no association between statin use and dementia incidence or progression.

One RCT reported on antacids and dementia risk as a secondary outcome, finding no increased risk of dementia with this medication [54].

One RCT examined dementia as a secondary outcome for anticoagulant use [92]. Four RCTs included dementia as a secondary outcome for NOACs vs warfarin. However, a Cochrane review of RCT data and anticoagulation [96] did not support their use to prevent cognitive decline.

Observational studies suggested reduced risk of dementia with NSAIDs and vitamin E, and vitamin C use. However, Cochrane reviews of RCTs of NSAIDs [97], vitamin E [98] and vitamin C [99] supplementation concluded that there was no evidence for their use in preventing dementia. In fact, RCTs [100, 101] were halted early due to harm associated with NSAID use.

Our umbrella review also found observational evidence suggesting an increased risk of dementia with ADT, benzodiazepines, Z-drugs, and anticholinergics, and decreased risk of dementia with lithium. There was little evidence for an association between dementia risk and antacids, anticonvulsants, antidepressants, and antipsychotics. We could not find available RCT evidence and only found observational studies for diabetes medications, NSAIDs, cancer treatments, most psychotropic medications apart from antidepressants, anticholinergics, and dietary supplements.

The majority of our included systematic reviews reported results from observational studies, and our reported associations should be interpreted in light of this. In particular, the risk of confounding by indication is a significant consideration as most studies did not include active comparator groups. Comparing people prescribed medications for a specific condition runs the risk of any difference observed being due to the underlying condition rather than the drug. Even when RCT evidence was available, dementia was only once included as a primary endpoint (eg. WHIMS study).

Results varied by medication subtype for some groups, such as for diabetes medications but not for others, such as anti-hypertensive medication, as discussed earlier. A Cochrane review of diabetes medications [102] and incident dementia found no evidence to support their general use for dementia prevention. Though our results were inconsistent for metformin use, the largest, most recent, and highest quality meta-analysis [61] that we included found reduced risk of dementia associated with its use. In addition, a recent study [103] examining early termination of metformin among people on type II diabetes treatments found an elevated risk of dementia among individuals stopping metformin, which was not explained by diabetes severity or insulin use (HR 1.21, 95% CI 1.12–1.30), suggesting a potential direct drug effect on dementia risk.

Recent meta-analyses have shown possible promise for two new diabetes drug classes: GLP-1 RAs and SGLT2 inhibitors. Animal models of GLP-1 RAs have shown neuroprotective effects including improved brain glucose metabolism and reduced inflammation [104]. SGLT2 inhibitors may modulate neuroprotective effects through their partial lipid solubility and the presence of SGLT2 receptors on neurons, especially in the hippocampus [105].

The meta-analyses we included for diabetes medications only presented observational data. However, initial results of a RCT of liraglutide, a GLP-1 RA, have shown reduced brain volume loss and slower decline in cognition in early AD compared to participants not taking liraglutide [106]. In addition, a study pooling data from three RCTs found lower risk of dementia among people randomised to GLP-1 RAs compared to placebo (HR 0.47, 95% CI 0.25–0.86) [11]. GLP-1 RAs are incretin-based diabetic agents, like DPP4 inhibitors, but a recent study concluded that when comparing these two drug classes the risk of dementia was still 23% lower among people taking GLP-1 RAs compared to people taking DPP4 inhibitors [107]. A systematic review published following our search found a reduction in dementia risk by 53% in three RCTs [108]. However, a recently published RCT examining GLP1-RAs in Parkinson’s showed no effect on cognition [109].

Proposed mechanisms by which systemic drugs may alter dementia risk include vascular risk factors, hormonal status, direct neurotoxic or neuroprotective effects, and neurotransmitter modulation. Treatment of hypertension appears to reduce dementia risk, and the increased risk of cardiovascular events and stroke associated with HRT use has been proposed to explain the increased dementia risk seen in the WHIMS RCTs. In contrast, other evidence suggests that lack of oestrogens may have a detrimental neurological effect [110], leading to increased levels of dementia among post-menopausal women. Studies in postmenopausal women have found higher levels of tau pathology and reduced brain glucose metabolism seen on positron emission tomograpy scanning than in premenopausal women [72]. Duration of oestrogen exposure has previously been shown to be potentially protective for dementia, with those in the prolonged duration of exposure group showing a 28% lower risk of dementia than those in the shortest exposure group [111]. In Nerattini et al. the risk of dementia with HRT varied according to the timing of administration and type of HRT, with HRT in mid-life (<10 years after last menstrual period) being protective. However, they found that administration of oestrogen and progesterone HRT in older women was associated with increased risk of dementia, such as in the WHIMS RCTs. This suggests that there may be chronological factors that determine the impact of HRT on dementia risk.

Androgens are purported to have neuroprotective effects [112], and suppression of these in mid-life may support the observed association between ADT and increased dementia risk. In particular, the association between ADT and dementia incidence was consistent at around a 20% increased risk of dementia, with similar results being found across most systematic reviews that we examined. Lipophilic statin subtypes have been proposed to have a direct neuroprotective effect due to their increased ability to cross the blood-brain barrier compared to hydrophilic statins, however there was no evidence to support this mechanistic hypothesis from available observational data. Anticholinergic medications are known to affect cognition acutely [113] and there is evidence of correlation between anticholinergic use over time and brain atrophy [114], which may be due to the direct effects of reduced cholinergic neuronal activity. The association seen with anticholinergics found with dementia risk seen in this review is consistent with the use of anticholinesterase inhibitors as symptomatic treatment for dementia [115].

There were limited data on dementia subtype, with most meta-analyses reporting on all-cause dementia. Meta-analyses reporting results for AD only found a similar association in size and direction of effect to that seen with all-cause dementia for antihypertensives, NSAIDs, and vitamin E supplementation. For antacids, the only meta-analysis [52] reporting exclusively on AD found a possible reduced incidence of dementia, in contrast to the other included meta-analyses. However, the study was small (*n* = 842) and CIs were very large and included the null (RR 0.83, 95% CI 0.39–1.78).

Confounding by indication limits our ability to isolate individual drug’s actions from the underlying condition in this review. Diabetes [4], hyperlipidaemia [116], hypertension [4], depression [4] and anxiety [117] have been proposed as risk factors for dementia, which may affect risk of dementia regardless of drug treatments, especially if treatment is not wholly successful. The definition of systemic medication exposure through using recorded prescriptions of medications in health records presents a challenge to our interpretation of these results due to a lack of active comparators. This issue has previously been raised as an important limitation in dementia pharmaco-epidemiological studies. For example, AlDawsari et al. [79] found that their effect estimate significantly changed for antidepressants when excluding one study that used an active comparator (paroxetine vs other SSRIs) [118]. All but one study in their meta-analysis of antidepressants relied on recorded prescriptions in health records, compared to people without a prescription. Comparing these two groups could lead to confounding by indication: as the comparator group would have included people without depression, leading to uncertainty as to whether the medication class or underlying condition alter dementia risk. Depression is a well-recognised risk factor for dementia [119]. In addition, the lack of specificity around indication for systemic medications is an issue with population-level data. For example, antidepressants are routinely prescribed for pain relief, anxiety, and obsessive-compulsive disorder, as well as for depression. Though many studies tried to control for indication, this was not always possible.

Severity of underlying conditions may have also influenced prescribing practices. In Ding et al. [31], the association between reduced dementia incidence and antihypertensive use was not seen among individuals with normal blood pressure readings, suggesting that any association may be due to control of a known risk factor for dementia rather than a direct drug effect. Lennon et al.’s [38] IPD meta-analysis was consistent with these results, finding elevated risk of dementia among participants with untreated hypertension compared to healthy controls, but no increased risk among participants with successfully treated hypertension.

Reverse causality could explain some of our results. In particular, protopathic bias is a possibility, where early symptoms of dementia may influence prescribing practices. For example, anxiety and sleep disturbance may be present in the prodromal phase of dementia [120], which could increase the odds of benzodiazepine or Z-drug prescription. When AlDawsari et al. [79] accounted for this possibility by only including analyses with a lag period between benzodiazepine or Z-drug prescription and diagnosis of dementia, they found no association between either drug and dementia outcome. This suggests that protopathic bias may explain the observed association.

Unmeasured or residual confounding may contribute to the observed associations. Risk factors for dementia were not consistently accounted for in included studies, and no systematic reviews that we included carried out sensitivity analyses to estimate whether inclusion of specific risk factors outlined by the Lancet Commission [4] altered effect estimates. For example, people taking vitamin supplements may be more likely to eat a healthy diet and be more physically active than those not taking supplements, which may explain the tendency towards reduced dementia risk noted with vitamin E supplementation. However, in Zhou et al. [89], only three out of 19 included studies adjusted for physical activity measures in their analyses. Other risk factors were better accounted for, such as education level, which was frequently adjusted for in analyses across drug types.

### Strengths and limitations

Our search strategy was comprehensive, using a worldwide classification of drugs to capture all systemic medications and subtypes. We searched multiple databases. Screening and quality rating of records were independently carried out. We used a validated quality assessment tool. We systematically and independently assessed the certainty of evidence using an established approach.

It was beyond the scope of this review to examine evidence for the relative effect of medication subtypes compared to each other with regards to dementia incidence (eg. CCBs compared to ACEIs). Medication classes may contain subtypes with differing mechanisms of action, limiting conclusions that can be drawn when considering medication classes as a whole. For example, studies did not separate centrally from peripherally acting CCBs.

We were unable to meta-analyse results due to the high levels of overlap found between studies. Publication bias may have influenced available results. However, only five meta-analyses reported evidence of publication bias in their assessments. In general, studies provided limited information regarding generalisability of findings. Very few meta-analyses included ethnicity data, and summary demographic data was often limited and necessitated retrieval of original individual studies.

A limitation of our study was that only 68% of our search results were screened by two authors. Our second search abstract results (dated April 2024) were screened by one author (NM) but all full-texts were independently screened by two authors (NM and CBR). There is a possibility that papers were missed in the later search when abstracts were only screened by one individual. However, we also searched reference lists of included papers, reducing the risk of missing relevant papers. Decisions to include articles where there was uncertainty were discussed and agreed with two senior authors (NM and JH).

There were a low number of RCTs included in the meta-analyses, and those that were included tended to not have dementia as a primary outcome of the RCT. Exceptions to this included the WHIMS for HRT use. This limited the power available in studies to assess dementia outcome, as follow-up was often halted following achievement of the primary outcome.

From the available evidence, we cannot conclude a causal association between dementia risk and systemic medications because the field is dominated by observational studies. As outlined above, observational studies may be subject to biases, such as immortal time, selection, surveillance and recall bias. Confounding by indication is a major limitation for many of the studies. In most included cohort and case-control studies, comparator groups were non-users of the medication of interest regardless of clinical indication. Exceptions to this included antihypertensives, ADT, lithium in bipolar disorder, anticoagulants, and medications for diabetes, where comparators were primarily people selected with the underlying condition that were not using the drug of interest. Though studies attempted to adjust for confounders and to include a lag period between medication prescription and diagnosis, this was not always possible. Several individual studies included in our presented meta-analyses attempted to address potential confounding by adjusting for underlying conditions such as depression, anxiety, and insomnia but residual confounding is always a risk.

Follow-up periods for most studies were relatively short. This limits conclusions that can be drawn regarding temporality given the lengthy preclinical and prodromal period of dementia, estimated at up to 14 years [121]. A retrospective cohort study that found multiple similar associations between dementia risk and systemic medications to our review showed that these relationships disappeared during sensitivity analysis when the authors excluded prescriptions less than 10 years prior to diagnosis of dementia [122]. There were some exceptions to this, for example Gallacher et al. [123] found an elevated risk of dementia with benzodiazepine use at 22 years follow-up.

Finally, administration of any systemic medication may be affected by timing, concomitant treatments, particular subtype of medication, as well as patient factors. For example, Nerattini et al. found that the effect of HRT on dementia risk varied according to duration of follow-up, timing of administration in the life-course, and subtype of HRT. This example illustrates the inherent challenge of interpreting meta-analysis in this field of study, as combining results from different population groups may miss the nuances of particular results.

## Conclusions

Our umbrella review used the GRADE framework to assess evidence of systemic medication use and dementia risk. In conclusion, we found moderate certainty evidence of a reduced risk of dementia associated with anti-hypertensives, statins, SGLT2 inhibitors, and GLP-1 RAs, and moderate certainty of an increased risk of dementia with anticholinergics. Based on these findings, we recommend proactive identification and treatment of hypertension as a modifiable risk factor for dementia. We recommend that clinicians identify and consider alternatives to anticholinergic medications in elderly patients, such as through using anticholinergic burden calculation tools. Assessment of anticholinergic burden should be carried out opportunistically, even if clinical presentation is unrelated to memory difficulties, and prior to prescription of new medications in later life.

There is a need for targeted RCT studies to examine the most promising systemic medications. These studies should be designed to measure dementia as a primary outcome and powered to identify causal relationships with systemic medications, ideally with long follow-up periods and recruiting participants from mid-life. We suggest that SGLT2 inhibitors and GLP-1 RAs may be promising drugs to pursue using this study design. Given the high cost, complexity, and long follow-up of such studies, high-quality observational studies with active comparator groups, appropriate lag periods, detailed assessment of confounding and risk factors for dementia, potentially using a target trial emulation framework [124], are needed. In addition, consideration of dementia subtyping through biomarkers will be essential in future to understand the potential mechanisms of action of individual drug classes on specific pathologies.

At present there is insufficient evidence of causality to support alterations in practice regarding most systemic medications and dementia, or that any of these agents have value as potential repurposed treatments. However, more work is needed to elucidate individual mechanisms of actions of new agents eg. GLP1 RAs. Though we did not find evidence to repurpose any existing medications, our findings confirmed some expected associations that will be useful for guiding clinical practice. Our findings strengthen the evidence for clinicians to avoid anticholinergics in cognitive impairment. The strongest evidence we found was for anti-hypertensive use in preventing dementia, reinforcing the need for proactive identification and treatment of hypertension as a risk factor for dementia.

## Supplementary information

1: PRIOR checklist for reporting umbrella reviews

2: Search strategy and terms

3: Full AMSTAR2 quality ratings

4: Citation matrices representing study overlap between systematic reviews
