# Supplementary material for: Systemic medications and dementia risk: a systematic umbrella review
Source: Mol Psychiatry. 2025 Jul 24;30(11):5578–99. doi: 10.1038/s41380-025-03129-3 (PMC12532590; doi:10.1038/s41380-025-03129-3)
Supplement: Supplementary file 2 — 2: Search strategy and terms [file 41380_2025_3129_MOESM2_ESM.pdf]

Supplement 2 : search strategy

1.	Stomatological*.mp. [mp=ab, hw, ti, tn, ot, dm, mf, dv, kw, fx, dq, tc, id, tm, mh, nm, kf, ox, px, rx, ui, sy]
2.	Antifungal*.mp. [mp=ab, hw, ti, tn, ot, dm, mf, dv, kw, fx, dq, tc, id, tm, mh, nm, kf, ox, px, rx, ui, sy]
3.	Antacid*.mp. [mp=ab, hw, ti, tn, ot, dm, mf, dv, kw, fx, dq, tc, id, tm, mh, nm, kf, ox, px, rx, ui, sy]
4.	Antiflatulent*.mp. [mp=ab, hw, ti, tn, ot, dm, mf, dv, kw, fx, dq, tc, id, tm, mh, nm, kf, ox, px, rx, ui, sy]
5.	CARMINATIVE*.mp. [mp=ab, hw, ti, tn, ot, dm, mf, dv, kw, fx, dq, tc, id, tm, mh, nm, kf, ox, px, rx, ui, sy]
6.	Antiulcerant*.mp. [mp=ab, hw, ti, tn, ot, dm, mf, dv, kw, fx, dq, tc, id, tm, mh, nm, kf, ox, px, rx, ui, sy]
7.	STOMACH DISORDER PREPARATION*.mp. [mp=ab, hw, ti, tn, ot, dm, mf, dv, kw, fx, dq, tc, id, tm, mh, nm, kf, ox, px, rx, ui, sy]
8.	ANTISPASMODIC*.mp. [mp=ab, hw, ti, tn, ot, dm, mf, dv, kw, fx, dq, tc, id, tm, mh, nm, kf, ox, px, rx, ui, sy]
9.	ANTICHOLINERGIC*.mp. [mp=ab, hw, ti, tn, ot, dm, mf, dv, kw, fx, dq, tc, id, tm, mh, nm, kf, ox, px, rx, ui, sy]
10.	BELLADONNA*.mp. [mp=ab, hw, ti, tn, ot, dm, mf, dv, kw, fx, dq, tc, id, tm, mh, nm, kf, ox, px, rx, ui, sy]
11.	ATARACTIC*.mp. [mp=ab, hw, ti, tn, ot, dm, mf, dv, kw, fx, dq, tc, id, tm, mh, nm, kf, ox, px, rx, ui, sy]
12.	PYCHOLEPTIC*.mp. [mp=ab, hw, ti, tn, ot, dm, mf, dv, kw, fx, dq, tc, id, tm, mh, nm, kf, ox, px, rx, ui, sy]
13.	ANALGESIC*.mp. [mp=ab, hw, ti, tn, ot, dm, mf, dv, kw, fx, dq, tc, id, tm, mh, nm, kf, ox, px, rx, ui, sy]
14.	GASTROPROKINETIC*.mp. [mp=ab, hw, ti, tn, ot, dm, mf, dv, kw, fx, dq, tc, id, tm, mh, nm, kf, ox, px, rx, ui, sy]
15.	PROPULSIVE*.mp. [mp=ab, hw, ti, tn, ot, dm, mf, dv, kw, fx, dq, tc, id, tm, mh, nm, kf, ox, px, rx, ui, sy]
16.	GASTRO*INTESTINAL SENSORIMOTOR MODULATOR*.mp. [mp=ab, hw, ti, tn, ot, dm, mf, dv, kw, fx, dq, tc, id, tm, mh, nm, kf, ox, px, rx, ui, sy]
17.	ANTIEMETIC*.mp. [mp=ab, hw, ti, tn, ot, dm, mf, dv, kw, fx, dq, tc, id, tm, mh, nm, kf, ox, px, rx, ui, sy]
18.	ANTINAUSEANT*.mp. [mp=ab, hw, ti, tn, ot, dm, mf, dv, kw, fx, dq, tc, id, tm, mh, nm, kf, ox, px, rx, ui, sy]
19.	Antihistamine*.mp. [mp=ab, hw, ti, tn, ot, dm, mf, dv, kw, fx, dq, tc, id, tm, mh, nm, kf, ox, px, rx, ui, sy]
20.	BILE THERAPY.mp. [mp=ab, hw, ti, tn, ot, dm, mf, dv, kw, fx, dq, tc, id, tm, mh, nm, kf, ox, px, rx, ui, sy]
21.	CHOLAGOGUE*.mp. [mp=ab, hw, ti, tn, ot, dm, mf, dv, kw, fx, dq, tc, id, tm, mh, nm, kf, ox, px, rx, ui, sy]
22.	HEPATIC PROTECTOR*.mp. [mp=ab, hw, ti, tn, ot, dm, mf, dv, kw, fx, dq, tc, id, tm, mh, nm, kf, ox, px, rx, ui, sy]
23.	LIPOTROPIC*.mp. [mp=ab, hw, ti, tn, ot, dm, mf, dv, kw, fx, dq, tc, id, tm, mh, nm, kf, ox, px, rx, ui, sy]
24.	LIVER THERAP*.mp. [mp=ab, hw, ti, tn, ot, dm, mf, dv, kw, fx, dq, tc, id, tm, mh, nm, kf, ox, px, rx, ui, sy]
25.	Drug* constipation.mp. [mp=ab, hw, ti, tn, ot, dm, mf, dv, kw, fx, dq, tc, id, tm, mh, nm, kf, ox, px, rx, ui, sy]
26.	BOWEL CLEANSER*.mp. [mp=ab, hw, ti, tn, ot, dm, mf, dv, kw, fx, dq, tc, id, tm, mh, nm, kf, ox, px, rx, ui, sy]
27.	INTESTINAL ANTIINFECTIVE*.mp. [mp=ab, hw, ti, tn, ot, dm, mf, dv, kw, fx, dq, tc, id, tm, mh, nm, kf, ox, px, rx, ui, sy]
28.	ANTIDIARRHOEAL*.mp. [mp=ab, hw, ti, tn, ot, dm, mf, dv, kw, fx, dq, tc, id, tm, mh, nm, kf, ox, px, rx, ui, sy]
29.	INTESTINAL ABSORBANT*.mp. [mp=ab, hw, ti, tn, ot, dm, mf, dv, kw, fx, dq, tc, id, tm, mh, nm, kf, ox, px, rx, ui, sy]
30.	ELECTROLYTE* WITH CARBOHYDRATE*.mp. [mp=ab, hw, ti, tn, ot, dm, mf, dv, kw, fx, dq, tc, id, tm, mh, nm, kf, ox, px, rx, ui, sy]

31.	ANTIPROPULSIVE*.mp. [mp=ab, hw, ti, tn, ot, dm, mf, dv, kw, fx, dq, tc, id, tm, mh, nm, kf, ox, px, rx, ui, sy]
32.	INTESTINAL ANTIINFLAMMATOR*.mp. [mp=ab, hw, ti, tn, ot, dm, mf, dv, kw, fx, dq, tc, id, tm, mh, nm, kf, ox, px, rx, ui, sy]
33.	ANTIDIARRHEAL MICROORGANISM*.mp. [mp=ab, hw, ti, tn, ot, dm, mf, dv, kw, fx, dq, tc, id, tm, mh, nm, kf, ox, px, rx, ui, sy]
34.	ORAL ELECTROLYTE*.mp. [mp=ab, hw, ti, tn, ot, dm, mf, dv, kw, fx, dq, tc, id, tm, mh, nm, kf, ox, px, rx, ui, sy]
35.	MOTILITY INHIBITOR*.mp. [mp=ab, hw, ti, tn, ot, dm, mf, dv, kw, fx, dq, tc, id, tm, mh, nm, kf, ox, px, rx, ui, sy]
36.	ANTIOBESITY PREPARATION*.mp. [mp=ab, hw, ti, tn, ot, dm, mf, dv, kw, fx, dq, tc, id, tm, mh, nm, kf, ox, px, rx, ui, sy]
37.	DIGESTIVE*.mp. [mp=ab, hw, ti, tn, ot, dm, mf, dv, kw, fx, dq, tc, id, tm, mh, nm, kf, ox, px, rx, ui, sy]
38.	DIGESTIVE ENZYME*.mp. [mp=ab, hw, ti, tn, ot, dm, mf, dv, kw, fx, dq, tc, id, tm, mh, nm, kf, ox, px, rx, ui, sy]
39.	INSULIN*.mp. [mp=ab, hw, ti, tn, ot, dm, mf, dv, kw, fx, dq, tc, id, tm, mh, nm, kf, ox, px, rx, ui, sy]
40.	ORAL BLOOD GLUCOSE LOWERING DRUG*.mp. [mp=ab, hw, ti, tn, ot, dm, mf, dv, kw, fx, dq, tc, id, tm, mh, nm, kf, ox, px, rx, ui, sy]
41.	HUMAN INSULIN*.mp. [mp=ab, hw, ti, tn, ot, dm, mf, dv, kw, fx, dq, tc, id, tm, mh, nm, kf, ox, px, rx, ui, sy]
42.	ANIMAL INSULIN*.mp. [mp=ab, hw, ti, tn, ot, dm, mf, dv, kw, fx, dq, tc, id, tm, mh, nm, kf, ox, px, rx, ui, sy]
43.	SULPHONYLUREA*.mp. [mp=ab, hw, ti, tn, ot, dm, mf, dv, kw, fx, dq, tc, id, tm, mh, nm, kf, ox, px, rx, ui, sy]
44.	BIGUANIDE*.mp. [mp=ab, hw, ti, tn, ot, dm, mf, dv, kw, fx, dq, tc, id, tm, mh, nm, kf, ox, px, rx, ui, sy]
45.	GLITAZONE*.mp. [mp=ab, hw, ti, tn, ot, dm, mf, dv, kw, fx, dq, tc, id, tm, mh, nm, kf, ox, px, rx, ui, sy]
46.	ALPHA-GLUCOSIDASE INHIBITOR*.mp. [mp=ab, hw, ti, tn, ot, dm, mf, dv, kw, fx, dq, tc, id, tm, mh, nm, kf, ox, px, rx, ui, sy]
47.	GLINIDE*.mp. [mp=ab, hw, ti, tn, ot, dm, mf, dv, kw, fx, dq, tc, id, tm, mh, nm, kf, ox, px, rx, ui, sy]
48.	DPP-IV INHIBITOR*.mp. [mp=ab, hw, ti, tn, ot, dm, mf, dv, kw, fx, dq, tc, id, tm, mh, nm, kf, ox, px, rx, ui, sy]
49.	SGLT2 INHIBITOR*.mp. [mp=ab, hw, ti, tn, ot, dm, mf, dv, kw, fx, dq, tc, id, tm, mh, nm, kf, ox, px, rx, ui, sy]
50.	GLP-1 AGONIST*.mp. [mp=ab, hw, ti, tn, ot, dm, mf, dv, kw, fx, dq, tc, id, tm, mh, nm, kf, ox, px, rx, ui, sy]
51.	VITAMIN*.mp. [mp=ab, hw, ti, tn, ot, dm, mf, dv, kw, fx, dq, tc, id, tm, mh, nm, kf, ox, px, rx, ui, sy]
52.	calcium.mp. [mp=ab, hw, ti, tn, ot, dm, mf, dv, kw, fx, dq, tc, id, tm, mh, nm, kf, ox, px, rx, ui, sy]
53.	POTASSIUM.mp. [mp=ab, hw, ti, tn, ot, dm, mf, dv, kw, fx, dq, tc, id, tm, mh, nm, kf, ox, px, rx, ui, sy]
54.	MINERAL SUPPLEMENT*.mp. [mp=ab, hw, ti, tn, ot, dm, mf, dv, kw, fx, dq, tc, id, tm, mh, nm, kf, ox, px, rx, ui, sy]
55.	TONIC*.mp. [mp=ab, hw, ti, tn, ot, dm, mf, dv, kw, fx, dq, tc, id, tm, mh, nm, kf, ox, px, rx, ui, sy]
56.	SYSTEMIC ANABOLIC HORMONE*.mp. [mp=ab, hw, ti, tn, ot, dm, mf, dv, kw, fx, dq, tc, id, tm, mh, nm, kf, ox, px, rx, ui, sy]
57.	SYSTEMIC ANABOLIC.mp. [mp=ab, hw, ti, tn, ot, dm, mf, dv, kw, fx, dq, tc, id, tm, mh, nm, kf, ox, px, rx, ui, sy]
58.	APPETITE STIMULANT*.mp. [mp=ab, hw, ti, tn, ot, dm, mf, dv, kw, fx, dq, tc, id, tm, mh, nm, kf, ox, px, rx, ui, sy]
59.	ALIMENTARY TRACT PRODUCT*.mp. [mp=ab, hw, ti, tn, ot, dm, mf, dv, kw, fx, dq, tc, id, tm, mh, nm, kf, ox, px, rx, ui, sy]
60.	METABOLISM PRODUCT*.mp. [mp=ab, hw, ti, tn, ot, dm, mf, dv, kw, fx, dq, tc, id, tm, mh, nm, kf, ox, px, rx, ui, sy]

61.	ANTITHROMBOTIC*.mp. [mp=ab, hw, ti, tn, ot, dm, mf, dv, kw, fx, dq, tc, id, tm, mh, nm, kf, ox, px, rx, ui, sy]
62.	VITAMIN K ANTAGONIST*.mp. [mp=ab, hw, ti, tn, ot, dm, mf, dv, kw, fx, dq, tc, id, tm, mh, nm, kf, ox, px, rx, ui, sy]
63.	HEPARIN*.mp. [mp=ab, hw, ti, tn, ot, dm, mf, dv, kw, fx, dq, tc, id, tm, mh, nm, kf, ox, px, rx, ui, sy]
64.	PLATETLET AGGREGATION INHIBITOR*.mp. [mp=ab, hw, ti, tn, ot, dm, mf, dv, kw, fx, dq, tc, id, tm, mh, nm, kf, ox, px, rx, ui, sy]
65.	FIBRINOLYTIC*.mp. [mp=ab, hw, ti, tn, ot, dm, mf, dv, kw, fx, dq, tc, id, tm, mh, nm, kf, ox, px, rx, ui, sy]
66.	DIRECT THROMBIN INHIBITOR*.mp. [mp=ab, hw, ti, tn, ot, dm, mf, dv, kw, fx, dq, tc, id, tm, mh, nm, kf, ox, px, rx, ui, sy]
67.	FACTOR XA INHIBITOR*.mp. [mp=ab, hw, ti, tn, ot, dm, mf, dv, kw, fx, dq, tc, id, tm, mh, nm, kf, ox, px, rx, ui, sy]
68.	ANTITHROMBOTIC*.mp. [mp=ab, hw, ti, tn, ot, dm, mf, dv, kw, fx, dq, tc, id, tm, mh, nm, kf, ox, px, rx, ui, sy]
69.	ANTIFIBRINOLYTIC*.mp. [mp=ab, hw, ti, tn, ot, dm, mf, dv, kw, fx, dq, tc, id, tm, mh, nm, kf, ox, px, rx, ui, sy]
70.	PROTEINASE INHIBITOR*.mp. [mp=ab, hw, ti, tn, ot, dm, mf, dv, kw, fx, dq, tc, id, tm, mh, nm, kf, ox, px, rx, ui, sy]
71.	VITAMIN K.mp. [mp=ab, hw, ti, tn, ot, dm, mf, dv, kw, fx, dq, tc, id, tm, mh, nm, kf, ox, px, rx, ui, sy]
72.	HEMOSTATIC*.mp. [mp=ab, hw, ti, tn, ot, dm, mf, dv, kw, fx, dq, tc, id, tm, mh, nm, kf, ox, px, rx, ui, sy]
73.	TISSUE SEALING PREPARATION*.mp. [mp=ab, hw, ti, tn, ot, dm, mf, dv, kw, fx, dq, tc, id, tm, mh, nm, kf, ox, px, rx, ui, sy]
74.	BLOOD COAGULATION MEDICATION*.mp. [mp=ab, hw, ti, tn, ot, dm, mf, dv, kw, fx, dq, tc, id, tm, mh, nm, kf, ox, px, rx, ui, sy]
75.	THROMBOPOIETIN AGONIST*.mp. [mp=ab, hw, ti, tn, ot, dm, mf, dv, kw, fx, dq, tc, id, tm, mh, nm, kf, ox, px, rx, ui, sy]
76.	SYSTEMIC HAEMOSTATIC*.mp. [mp=ab, hw, ti, tn, ot, dm, mf, dv, kw, fx, dq, tc, id, tm, mh, nm, kf, ox, px, rx, ui, sy]
77.	HAEMATINIC*.mp. [mp=ab, hw, ti, tn, ot, dm, mf, dv, kw, fx, dq, tc, id, tm, mh, nm, kf, ox, px, rx, ui, sy]
78.	IRON.mp. [mp=ab, hw, ti, tn, ot, dm, mf, dv, kw, fx, dq, tc, id, tm, mh, nm, kf, ox, px, rx, ui, sy]
79.	VITAMIN B12.mp. [mp=ab, hw, ti, tn, ot, dm, mf, dv, kw, fx, dq, tc, id, tm, mh, nm, kf, ox, px, rx, ui, sy]
80.	FOLIC ACID.mp. [mp=ab, hw, ti, tn, ot, dm, mf, dv, kw, fx, dq, tc, id, tm, mh, nm, kf, ox, px, rx, ui, sy]
81.	FOLINIC ACID.mp. [mp=ab, hw, ti, tn, ot, dm, mf, dv, kw, fx, dq, tc, id, tm, mh, nm, kf, ox, px, rx, ui, sy]
82.	ANTI-ANAEMIC MEDICATION*.mp. [mp=ab, hw, ti, tn, ot, dm, mf, dv, kw, fx, dq, tc, id, tm, mh, nm, kf, ox, px, rx, ui, sy]
83.	ERYTHROPOIETIN.mp. [mp=ab, hw, ti, tn, ot, dm, mf, dv, kw, fx, dq, tc, id, tm, mh, nm, kf, ox, px, rx, ui, sy]
84.	BLOOD PRODUCT*.mp. [mp=ab, hw, ti, tn, ot, dm, mf, dv, kw, fx, dq, tc, id, tm, mh, nm, kf, ox, px, rx, ui, sy]
85.	INTRAVENOUS SOLUTION*.mp. [mp=ab, hw, ti, tn, ot, dm, mf, dv, kw, fx, dq, tc, id, tm, mh, nm, kf, ox, px, rx, ui, sy]
86.	IRRIGATING SOLUTION*.mp. [mp=ab, hw, ti, tn, ot, dm, mf, dv, kw, fx, dq, tc, id, tm, mh, nm, kf, ox, px, rx, ui, sy]
87.	PERITONEAL DIALYTIC*.mp. [mp=ab, hw, ti, tn, ot, dm, mf, dv, kw, fx, dq, tc, id, tm, mh, nm, kf, ox, px, rx, ui, sy]
88.	HEMODIALYTIC*.mp. [mp=ab, hw, ti, tn, ot, dm, mf, dv, kw, fx, dq, tc, id, tm, mh, nm, kf, ox, px, rx, ui, sy]
89.	HEMOFILTRATE*.mp. [mp=ab, hw, ti, tn, ot, dm, mf, dv, kw, fx, dq, tc, id, tm, mh, nm, kf, ox, px, rx, ui, sy]

90. PERITONEAL DIALYSIS SOLUTION*.mp. [mp=ab, hw, ti, tn, ot, dm, mf, dv, kw, fx, dq, tc, id, tm, mh, nm, kf, ox, px, rx, ui, sy]
91. HAEMODIALYSIS SOLUTION*.mp. [mp=ab, hw, ti, tn, ot, dm, mf, dv, kw, fx, dq, tc, id, tm, mh, nm, kf, ox, px, rx, ui, sy]
92. HAEMOFILTRATION.mp. [mp=ab, hw, ti, tn, ot, dm, mf, dv, kw, fx, dq, tc, id, tm, mh, nm, kf, ox, px, rx, ui, sy]
93. HEMATOLOGICAL AGENT*.mp. [mp=ab, hw, ti, tn, ot, dm, mf, dv, kw, fx, dq, tc, id, tm, mh, nm, kf, ox, px, rx, ui, sy]
94. HYALURONIDASE.mp. [mp=ab, hw, ti, tn, ot, dm, mf, dv, kw, fx, dq, tc, id, tm, mh, nm, kf, ox, px, rx, ui, sy]
95. Trypsin.mp. [mp=ab, hw, ti, tn, ot, dm, mf, dv, kw, fx, dq, tc, id, tm, mh, nm, kf, ox, px, rx, ui, sy]
96. HEREDITARY ANGIOEDEMA MEDICATION*.mp. [mp=ab, hw, ti, tn, ot, dm, mf, dv, kw, fx, dq, tc, id, tm, mh, nm, kf, ox, px, rx, ui, sy]
97. CARDIAC GLYCOSIDE*.mp. [mp=ab, hw, ti, tn, ot, dm, mf, dv, kw, fx, dq, tc, id, tm, mh, nm, kf, ox, px, rx, ui, sy]
98. ANTI-ARRHYTHMIC*.mp. [mp=ab, hw, ti, tn, ot, dm, mf, dv, kw, fx, dq, tc, id, tm, mh, nm, kf, ox, px, rx, ui, sy]
99. CARDIAC STIMULANT*.mp. [mp=ab, hw, ti, tn, ot, dm, mf, dv, kw, fx, dq, tc, id, tm, mh, nm, kf, ox, px, rx, ui, sy]
100. POSITIVE INOTROPIC AGENT*.mp. [mp=ab, hw, ti, tn, ot, dm, mf, dv, kw, fx, dq, tc, id, tm, mh, nm, kf, ox, px, rx, ui, sy]
101. VASODILATORS IN CARDIAC DISEASES.mp. [mp=ab, hw, ti, tn, ot, dm, mf, dv, kw, fx, dq, tc, id, tm, mh, nm, kf, ox, px, rx, ui, sy]
102. NITRITE*.mp. [mp=ab, hw, ti, tn, ot, dm, mf, dv, kw, fx, dq, tc, id, tm, mh, nm, kf, ox, px, rx, ui, sy]
103. NITRATE*.mp. [mp=ab, hw, ti, tn, ot, dm, mf, dv, kw, fx, dq, tc, id, tm, mh, nm, kf, ox, px, rx, ui, sy]
104. VASODILATOR*.mp. [mp=ab, hw, ti, tn, ot, dm, mf, dv, kw, fx, dq, tc, id, tm, mh, nm, kf, ox, px, rx, ui, sy]
105. CALCIUM ANTAGONIST*.mp. [mp=ab, hw, ti, tn, ot, dm, mf, dv, kw, fx, dq, tc, id, tm, mh, nm, kf, ox, px, rx, ui, sy]
106. Trimetazidine.mp. [mp=ab, hw, ti, tn, ot, dm, mf, dv, kw, fx, dq, tc, id, tm, mh, nm, kf, ox, px, rx, ui, sy]
107. Flosequinan.mp. [mp=ab, hw, ti, tn, ot, dm, mf, dv, kw, fx, dq, tc, id, tm, mh, nm, kf, ox, px, rx, ui, sy]
108. Adenosine.mp. [mp=ab, hw, ti, tn, ot, dm, mf, dv, kw, fx, dq, tc, id, tm, mh, nm, kf, ox, px, rx, ui, sy]
109. Ivabradine.mp. [mp=ab, hw, ti, tn, ot, dm, mf, dv, kw, fx, dq, tc, id, tm, mh, nm, kf, ox, px, rx, ui, sy]
110. Levocarnitine.mp. [mp=ab, hw, ti, tn, ot, dm, mf, dv, kw, fx, dq, tc, id, tm, mh, nm, kf, ox, px, rx, ui, sy]
111. Nesiritide.mp. [mp=ab, hw, ti, tn, ot, dm, mf, dv, kw, fx, dq, tc, id, tm, mh, nm, kf, ox, px, rx, ui, sy]
112. ANTIHYPERTENSIVE*.mp. [mp=ab, hw, ti, tn, ot, dm, mf, dv, kw, fx, dq, tc, id, tm, mh, nm, kf, ox, px, rx, ui, sy]
113. DIURETIC*.mp. [mp=ab, hw, ti, tn, ot, dm, mf, dv, kw, fx, dq, tc, id, tm, mh, nm, kf, ox, px, rx, ui, sy]
114. PERIPHERAL VASODILATOR*.mp. [mp=ab, hw, ti, tn, ot, dm, mf, dv, kw, fx, dq, tc, id, tm, mh, nm, kf, ox, px, rx, ui, sy]
115. CEREBRAL VASODILATOR*.mp. [mp=ab, hw, ti, tn, ot, dm, mf, dv, kw, fx, dq, tc, id, tm, mh, nm, kf, ox, px, rx, ui, sy]
116. VARICOSE THERAP*.mp. [mp=ab, hw, ti, tn, ot, dm, mf, dv, kw, fx, dq, tc, id, tm, mh, nm, kf, ox, px, rx, ui, sy]
117. ANTIVARICOSE.mp. [mp=ab, hw, ti, tn, ot, dm, mf, dv, kw, fx, dq, tc, id, tm, mh, nm, kf, ox, px, rx, ui, sy]
118. CAPILLARY STABILIZING AGENT*.mp. [mp=ab, hw, ti, tn, ot, dm, mf, dv, kw, fx, dq, tc, id, tm, mh, nm, kf, ox, px, rx, ui, sy]
119. Ambrisentan.mp. [mp=ab, hw, ti, tn, ot, dm, mf, dv, kw, fx, dq, tc, id, tm, mh, nm, kf, ox, px, rx, ui, sy]

120. bosentan.mp. [mp=ab, hw, ti, tn, ot, dm, mf, dv, kw, fx, dq, tc, id, tm, mh, nm, kf, ox, px, rx, ui, sy]
121. macitentan.mp. [mp=ab, hw, ti, tn, ot, dm, mf, dv, kw, fx, dq, tc, id, tm, mh, nm, kf, ox, px, rx, ui, sy]
122. riociguat.mp. [mp=ab, hw, ti, tn, ot, dm, mf, dv, kw, fx, dq, tc, id, tm, mh, nm, kf, ox, px, rx, ui, sy]
123. sitaxentan.mp. [mp=ab, hw, ti, tn, ot, dm, mf, dv, kw, fx, dq, tc, id, tm, mh, nm, kf, ox, px, rx, ui, sy]
124. Iloprost.mp. [mp=ab, hw, ti, tn, ot, dm, mf, dv, kw, fx, dq, tc, id, tm, mh, nm, kf, ox, px, rx, ui, sy]
125. PDE5 inhibitor*.mp. [mp=ab, hw, ti, tn, ot, dm, mf, dv, kw, fx, dq, tc, id, tm, mh, nm, kf, ox, px, rx, ui, sy]
126. BETA BLOCKING AGENT*.mp. [mp=ab, hw, ti, tn, ot, dm, mf, dv, kw, fx, dq, tc, id, tm, mh, nm, kf, ox, px, rx, ui, sy]
127. BETA BLOCKER*.mp. [mp=ab, hw, ti, tn, ot, dm, mf, dv, kw, fx, dq, tc, id, tm, mh, nm, kf, ox, px, rx, ui, sy]
128. THIAZIDE*.mp. [mp=ab, hw, ti, tn, ot, dm, mf, dv, kw, fx, dq, tc, id, tm, mh, nm, kf, ox, px, rx, ui, sy]
129. DIURETIC*.mp. [mp=ab, hw, ti, tn, ot, dm, mf, dv, kw, fx, dq, tc, id, tm, mh, nm, kf, ox, px, rx, ui, sy]
130. VASODILATOR*.mp. [mp=ab, hw, ti, tn, ot, dm, mf, dv, kw, fx, dq, tc, id, tm, mh, nm, kf, ox, px, rx, ui, sy]
131. CALCIUM ANTAGONIST*.mp. [mp=ab, hw, ti, tn, ot, dm, mf, dv, kw, fx, dq, tc, id, tm, mh, nm, kf, ox, px, rx, ui, sy]
132. CALCIUM CHANNEL BLOCKER*.mp. [mp=ab, hw, ti, tn, ot, dm, mf, dv, kw, fx, dq, tc, id, tm, mh, nm, kf, ox, px, rx, ui, sy]
133. SELECTIVE CALCIUM CHANNEL BLOCKER*.mp. [mp=ab, hw, ti, tn, ot, dm, mf, dv, kw, fx, dq, tc, id, tm, mh, nm, kf, ox, px, rx, ui, sy]
134. NON-SELECTIVE CALCIUM CHANNEL BLOCKER*.mp. [mp=ab, hw, ti, tn, ot, dm, mf, dv, kw, fx, dq, tc, id, tm, mh, nm, kf, ox, px, rx, ui, sy]
135. ACE INHIBITOR*.mp. [mp=ab, hw, ti, tn, ot, dm, mf, dv, kw, fx, dq, tc, id, tm, mh, nm, kf, ox, px, rx, ui, sy]
136. ANGIOTENSIN II ANTAGONIST*.mp. [mp=ab, hw, ti, tn, ot, dm, mf, dv, kw, fx, dq, tc, id, tm, mh, nm, kf, ox, px, rx, ui, sy]
137. RENIN-ANGIOTENSIN SYSTEM AGENT*.mp. [mp=ab, hw, ti, tn, ot, dm, mf, dv, kw, fx, dq, tc, id, tm, mh, nm, kf, ox, px, rx, ui, sy]
138. LIPID MODIFYING AGENT*.mp. [mp=ab, hw, ti, tn, ot, dm, mf, dv, kw, fx, dq, tc, id, tm, mh, nm, kf, ox, px, rx, ui, sy]
139. LIPID LOWERING.mp. [mp=ab, hw, ti, tn, ot, dm, mf, dv, kw, fx, dq, tc, id, tm, mh, nm, kf, ox, px, rx, ui, sy]
140. CHOLESTEROL REGULATING.mp. [mp=ab, hw, ti, tn, ot, dm, mf, dv, kw, fx, dq, tc, id, tm, mh, nm, kf, ox, px, rx, ui, sy]
141. STATIN*.mp. [mp=ab, hw, ti, tn, ot, dm, mf, dv, kw, fx, dq, tc, id, tm, mh, nm, kf, ox, px, rx, ui, sy]
142. ANTI-ATHEROMA.mp. [mp=ab, hw, ti, tn, ot, dm, mf, dv, kw, fx, dq, tc, id, tm, mh, nm, kf, ox, px, rx, ui, sy]
143. LIPID REGULAT*.mp. [mp=ab, hw, ti, tn, ot, dm, mf, dv, kw, fx, dq, tc, id, tm, mh, nm, kf, ox, px, rx, ui, sy]
144. SYSTEMIC ANTIFUNGAL*.mp. [mp=ab, hw, ti, tn, ot, dm, mf, dv, kw, fx, dq, tc, id, tm, mh, nm, kf, ox, px, rx, ui, sy]
145. Betacarotene.mp. [mp=ab, hw, ti, tn, ot, dm, mf, dv, kw, fx, dq, tc, id, tm, mh, nm, kf, ox, px, rx, ui, sy]
146. Aminobenzoic acid.mp. [mp=ab, hw, ti, tn, ot, dm, mf, dv, kw, fx, dq, tc, id, tm, mh, nm, kf, ox, px, rx, ui, sy]
147. CICATRIZANT*.mp. [mp=ab, hw, ti, tn, ot, dm, mf, dv, kw, fx, dq, tc, id, tm, mh, nm, kf, ox, px, rx, ui, sy]
148. Bromelain*.mp. [mp=ab, hw, ti, tn, ot, dm, mf, dv, kw, fx, dq, tc, id, tm, mh, nm, kf, ox, px, rx, ui, sy]
149. ANTIPRURITIC*.mp. [mp=ab, hw, ti, tn, ot, dm, mf, dv, kw, fx, dq, tc, id, tm, mh, nm, kf, ox, px, rx, ui, sy]
150. ANTIHISTAMINE*.mp. [mp=ab, hw, ti, tn, ot, dm, mf, dv, kw, fx, dq, tc, id, tm, mh, nm, kf, ox, px, rx, ui, sy]
151. ANAESTETHIC*.mp. [mp=ab, hw, ti, tn, ot, dm, mf, dv, kw, fx, dq, tc, id, tm, mh, nm, kf, ox, px, rx, ui, sy]
152. ANTIPSORIA*.mp. [mp=ab, hw, ti, tn, ot, dm, mf, dv, kw, fx, dq, tc, id, tm, mh, nm, kf, ox, px, rx, ui, sy]
153. Alefacept.mp. [mp=ab, hw, ti, tn, ot, dm, mf, dv, kw, fx, dq, tc, id, tm, mh, nm, kf, ox, px, rx, ui, sy]

154. ustekinumab.mp. [mp=ab, hw, ti, tn, ot, dm, mf, dv, kw, fx, dq, tc, id, tm, mh, nm, kf, ox, px, rx, ui, sy]
155. Methotrexate.mp. [mp=ab, hw, ti, tn, ot, dm, mf, dv, kw, fx, dq, tc, id, tm, mh, nm, kf, ox, px, rx, ui, sy]
156. NONSTEROIDAL FOR INFLAMMATORY SKIN.mp. [mp=ab, hw, ti, tn, ot, dm, mf, dv, kw, fx, dq, tc, id, tm, mh, nm, kf, ox, px, rx, ui, sy]
157. Alitretinoin.mp. [mp=ab, hw, ti, tn, ot, dm, mf, dv, kw, fx, dq, tc, id, tm, mh, nm, kf, ox, px, rx, ui, sy]
158. Aminolevulinic acid.mp. [mp=ab, hw, ti, tn, ot, dm, mf, dv, kw, fx, dq, tc, id, tm, mh, nm, kf, ox, px, rx, ui, sy]
159. ANTIBIOTIC*.mp. [mp=ab, hw, ti, tn, ot, dm, mf, dv, kw, fx, dq, tc, id, tm, mh, nm, kf, ox, px, rx, ui, sy]
160. CHEMOTHERAPEUTIC*.mp. [mp=ab, hw, ti, tn, ot, dm, mf, dv, kw, fx, dq, tc, id, tm, mh, nm, kf, ox, px, rx, ui, sy]
161. ANTI-ACNE.mp. [mp=ab, hw, ti, tn, ot, dm, mf, dv, kw, fx, dq, tc, id, tm, mh, nm, kf, ox, px, rx, ui, sy]
162. SYSTEMIC ACNE.mp. [mp=ab, hw, ti, tn, ot, dm, mf, dv, kw, fx, dq, tc, id, tm, mh, nm, kf, ox, px, rx, ui, sy]
163. ORAL ACNE.mp. [mp=ab, hw, ti, tn, ot, dm, mf, dv, kw, fx, dq, tc, id, tm, mh, nm, kf, ox, px, rx, ui, sy]
164. estrogen.mp. [mp=ab, hw, ti, tn, ot, dm, mf, dv, kw, fx, dq, tc, id, tm, mh, nm, kf, ox, px, rx, ui, sy]
165. cyproterone.mp. [mp=ab, hw, ti, tn, ot, dm, mf, dv, kw, fx, dq, tc, id, tm, mh, nm, kf, ox, px, rx, ui, sy]
166. Tacrolimus.mp. [mp=ab, hw, ti, tn, ot, dm, mf, dv, kw, fx, dq, tc, id, tm, mh, nm, kf, ox, px, rx, ui, sy]
167. Alitretinoin.mp. [mp=ab, hw, ti, tn, ot, dm, mf, dv, kw, fx, dq, tc, id, tm, mh, nm, kf, ox, px, rx, ui, sy]
168. Brimonidine.mp. [mp=ab, hw, ti, tn, ot, dm, mf, dv, kw, fx, dq, tc, id, tm, mh, nm, kf, ox, px, rx, ui, sy]
169. Ivermectin.mp. [mp=ab, hw, ti, tn, ot, dm, mf, dv, kw, fx, dq, tc, id, tm, mh, nm, kf, ox, px, rx, ui, sy]
170. TRICHOMONACIDE*.mp. [mp=ab, hw, ti, tn, ot, dm, mf, dv, kw, fx, dq, tc, id, tm, mh, nm, kf, ox, px, rx, ui, sy]
171. ANTIFUNGAL*.mp. [mp=ab, hw, ti, tn, ot, dm, mf, dv, kw, fx, dq, tc, id, tm, mh, nm, kf, ox, px, rx, ui, sy]
172. ANTIBACTERIAL*.mp. [mp=ab, hw, ti, tn, ot, dm, mf, dv, kw, fx, dq, tc, id, tm, mh, nm, kf, ox, px, rx, ui, sy]
173. ANTISEPTIC*.mp. [mp=ab, hw, ti, tn, ot, dm, mf, dv, kw, fx, dq, tc, id, tm, mh, nm, kf, ox, px, rx, ui, sy]
174. CORTICOSTEROID*.mp. [mp=ab, hw, ti, tn, ot, dm, mf, dv, kw, fx, dq, tc, id, tm, mh, nm, kf, ox, px, rx, ui, sy]
175. STEROID*.mp. [mp=ab, hw, ti, tn, ot, dm, mf, dv, kw, fx, dq, tc, id, tm, mh, nm, kf, ox, px, rx, ui, sy]
176. PROLACTIN INHIBITOR*.mp. [mp=ab, hw, ti, tn, ot, dm, mf, dv, kw, fx, dq, tc, id, tm, mh, nm, kf, ox, px, rx, ui, sy]
177. Bromocriptine.mp. [mp=ab, hw, ti, tn, ot, dm, mf, dv, kw, fx, dq, tc, id, tm, mh, nm, kf, ox, px, rx, ui, sy]
178. Cabergoline.mp. [mp=ab, hw, ti, tn, ot, dm, mf, dv, kw, fx, dq, tc, id, tm, mh, nm, kf, ox, px, rx, ui, sy]
179. HORMONAL CONTRACEPTIVE*.mp. [mp=ab, hw, ti, tn, ot, dm, mf, dv, kw, fx, dq, tc, id, tm, mh, nm, kf, ox, px, rx, ui, sy]
180. progestogen*.mp. [mp=ab, hw, ti, tn, ot, dm, mf, dv, kw, fx, dq, tc, id, tm, mh, nm, kf, ox, px, rx, ui, sy]
181. Ulipristal.mp. [mp=ab, hw, ti, tn, ot, dm, mf, dv, kw, fx, dq, tc, id, tm, mh, nm, kf, ox, px, rx, ui, sy]
182. Mifepristone.mp. [mp=ab, hw, ti, tn, ot, dm, mf, dv, kw, fx, dq, tc, id, tm, mh, nm, kf, ox, px, rx, ui, sy]
183. ANDROGEN*.mp. [mp=ab, hw, ti, tn, ot, dm, mf, dv, kw, fx, dq, tc, id, tm, mh, nm, kf, ox, px, rx, ui, sy]
184. OESTROGEN*.mp. [mp=ab, hw, ti, tn, ot, dm, mf, dv, kw, fx, dq, tc, id, tm, mh, nm, kf, ox, px, rx, ui, sy]
185. PROGESTOGEN*.mp. [mp=ab, hw, ti, tn, ot, dm, mf, dv, kw, fx, dq, tc, id, tm, mh, nm, kf, ox, px, rx, ui, sy]
186. Flumedroxone.mp. [mp=ab, hw, ti, tn, ot, dm, mf, dv, kw, fx, dq, tc, id, tm, mh, nm, kf, ox, px, rx, ui, sy]
187. ANDROGEN*.mp. [mp=ab, hw, ti, tn, ot, dm, mf, dv, kw, fx, dq, tc, id, tm, mh, nm, kf, ox, px, rx, ui, sy]
188. GONADOTROPHIN*.mp. [mp=ab, hw, ti, tn, ot, dm, mf, dv, kw, fx, dq, tc, id, tm, mh, nm, kf, ox, px, rx, ui, sy]

189. ANTIANDROGEN*.mp. [mp=ab, hw, ti, tn, ot, dm, mf, dv, kw, fx, dq, tc, id, tm, mh, nm, kf, ox, px, rx, ui, sy]
190. cyproterone.mp. [mp=ab, hw, ti, tn, ot, dm, mf, dv, kw, fx, dq, tc, id, tm, mh, nm, kf, ox, px, rx, ui, sy]
191. OESTROGEN RECEPTOR MODULATOR*.mp. [mp=ab, hw, ti, tn, ot, dm, mf, dv, kw, fx, dq, tc, id, tm, mh, nm, kf, ox, px, rx, ui, sy]
192. Bazedoxifene.mp. [mp=ab, hw, ti, tn, ot, dm, mf, dv, kw, fx, dq, tc, id, tm, mh, nm, kf, ox, px, rx, ui, sy]
193. lasofoxifene.mp. [mp=ab, hw, ti, tn, ot, dm, mf, dv, kw, fx, dq, tc, id, tm, mh, nm, kf, ox, px, rx, ui, sy]
194. ospemifene.mp. [mp=ab, hw, ti, tn, ot, dm, mf, dv, kw, fx, dq, tc, id, tm, mh, nm, kf, ox, px, rx, ui, sy]
195. raloxifene.mp. [mp=ab, hw, ti, tn, ot, dm, mf, dv, kw, fx, dq, tc, id, tm, mh, nm, kf, ox, px, rx, ui, sy]
196. Tibolone.mp. [mp=ab, hw, ti, tn, ot, dm, mf, dv, kw, fx, dq, tc, id, tm, mh, nm, kf, ox, px, rx, ui, sy]
197. URINARY ANTI-INFECTIVE*.mp. [mp=ab, hw, ti, tn, ot, dm, mf, dv, kw, fx, dq, tc, id, tm, mh, nm, kf, ox, px, rx, ui, sy]
198. quinolone*.mp. [mp=ab, hw, ti, tn, ot, dm, mf, dv, kw, fx, dq, tc, id, tm, mh, nm, kf, ox, px, rx, ui, sy]
199. Erectile dysfunction medication*.mp. [mp=ab, hw, ti, tn, ot, dm, mf, dv, kw, fx, dq, tc, id, tm, mh, nm, kf, ox, px, rx, ui, sy]
200. Urinary incontinence medication*.mp. [mp=ab, hw, ti, tn, ot, dm, mf, dv, kw, fx, dq, tc, id, tm, mh, nm, kf, ox, px, rx, ui, sy]
201. antispasmodic*.mp. [mp=ab, hw, ti, tn, ot, dm, mf, dv, kw, fx, dq, tc, id, tm, mh, nm, kf, ox, px, rx, ui, sy]
202. BENIGN PROSTATIC HYPERTROPHY medication*.mp. [mp=ab, hw, ti, tn, ot, dm, mf, dv, kw, fx, dq, tc, id, tm, mh, nm, kf, ox, px, rx, ui, sy]
203. Indoramin.mp. [mp=ab, hw, ti, tn, ot, dm, mf, dv, kw, fx, dq, tc, id, tm, mh, nm, kf, ox, px, rx, ui, sy]
204. URINARY INCONTINENCE PRODUCT*.mp. [mp=ab, hw, ti, tn, ot, dm, mf, dv, kw, fx, dq, tc, id, tm, mh, nm, kf, ox, px, rx, ui, sy]
205. URINARY INCONTINENCE TREATMENT*.mp. [mp=ab, hw, ti, tn, ot, dm, mf, dv, kw, fx, dq, tc, id, tm, mh, nm, kf, ox, px, rx, ui, sy]
206. URINARY INCONTINENCE THERAP*.mp. [mp=ab, hw, ti, tn, ot, dm, mf, dv, kw, fx, dq, tc, id, tm, mh, nm, kf, ox, px, rx, ui, sy]
207. Phentolamine.mp. [mp=ab, hw, ti, tn, ot, dm, mf, dv, kw, fx, dq, tc, id, tm, mh, nm, kf, ox, px, rx, ui, sy]
208. THYROID PREPARATION*.mp. [mp=ab, hw, ti, tn, ot, dm, mf, dv, kw, fx, dq, tc, id, tm, mh, nm, kf, ox, px, rx, ui, sy]
209. THYROID Medication*.mp. [mp=ab, hw, ti, tn, ot, dm, mf, dv, kw, fx, dq, tc, id, tm, mh, nm, kf, ox, px, rx, ui, sy]
210. ANTERIOR PITUITARY HORMONE*.mp. [mp=ab, hw, ti, tn, ot, dm, mf, dv, kw, fx, dq, tc, id, tm, mh, nm, kf, ox, px, rx, ui, sy]
211. ANTERIOR PITUITARY HORMONES ANALOGUE*.mp. [mp=ab, hw, ti, tn, ot, dm, mf, dv, kw, fx, dq, tc, id, tm, mh, nm, kf, ox, px, rx, ui, sy]
212. GROWTH HORMONE*.mp. [mp=ab, hw, ti, tn, ot, dm, mf, dv, kw, fx, dq, tc, id, tm, mh, nm, kf, ox, px, rx, ui, sy]
213. POSTERIOR PITUITARY HORMONE*.mp. [mp=ab, hw, ti, tn, ot, dm, mf, dv, kw, fx, dq, tc, id, tm, mh, nm, kf, ox, px, rx, ui, sy]
214. ANTIDIURETIC HORMONE*.mp. [mp=ab, hw, ti, tn, ot, dm, mf, dv, kw, fx, dq, tc, id, tm, mh, nm, kf, ox, px, rx, ui, sy]
215. LABOUR INDUCER*.mp. [mp=ab, hw, ti, tn, ot, dm, mf, dv, kw, fx, dq, tc, id, tm, mh, nm, kf, ox, px, rx, ui, sy]
216. HYPOTHALAMIC HORMONE*.mp. [mp=ab, hw, ti, tn, ot, dm, mf, dv, kw, fx, dq, tc, id, tm, mh, nm, kf, ox, px, rx, ui, sy]
217. CORTICOSTEROID*.mp. [mp=ab, hw, ti, tn, ot, dm, mf, dv, kw, fx, dq, tc, id, tm, mh, nm, kf, ox, px, rx, ui, sy]

218. ANTIADRENAL PREPARATION*.mp. [mp=ab, hw, ti, tn, ot, dm, mf, dv, kw, fx, dq, tc, id, tm, mh, nm, kf, ox, px, rx, ui, sy]
219. Trilostane.mp. [mp=ab, hw, ti, tn, ot, dm, mf, dv, kw, fx, dq, tc, id, tm, mh, nm, kf, ox, px, rx, ui, sy]
220. THYROID PREPARATION*.mp. [mp=ab, hw, ti, tn, ot, dm, mf, dv, kw, fx, dq, tc, id, tm, mh, nm, kf, ox, px, rx, ui, sy]
221. THYROID MEDICATION*.mp. [mp=ab, hw, ti, tn, ot, dm, mf, dv, kw, fx, dq, tc, id, tm, mh, nm, kf, ox, px, rx, ui, sy]
222. Thyrotrophin.mp. [mp=ab, hw, ti, tn, ot, dm, mf, dv, kw, fx, dq, tc, id, tm, mh, nm, kf, ox, px, rx, ui, sy]
223. ANTITHYROID.mp. [mp=ab, hw, ti, tn, ot, dm, mf, dv, kw, fx, dq, tc, id, tm, mh, nm, kf, ox, px, rx, ui, sy]
224. ANTI-THYROID.mp. [mp=ab, hw, ti, tn, ot, dm, mf, dv, kw, fx, dq, tc, id, tm, mh, nm, kf, ox, px, rx, ui, sy]
225. IODINE.mp. [mp=ab, hw, ti, tn, ot, dm, mf, dv, kw, fx, dq, tc, id, tm, mh, nm, kf, ox, px, rx, ui, sy]
226. GLYCOGENOLYTIC HORMONE*.mp. [mp=ab, hw, ti, tn, ot, dm, mf, dv, kw, fx, dq, tc, id, tm, mh, nm, kf, ox, px, rx, ui, sy]
227. GLUCAGON.mp. [mp=ab, hw, ti, tn, ot, dm, mf, dv, kw, fx, dq, tc, id, tm, mh, nm, kf, ox, px, rx, ui, sy]
228. PARATHYROID HORMONE*.mp. [mp=ab, hw, ti, tn, ot, dm, mf, dv, kw, fx, dq, tc, id, tm, mh, nm, kf, ox, px, rx, ui, sy]
229. PARATHYROID ANALOGUE*.mp. [mp=ab, hw, ti, tn, ot, dm, mf, dv, kw, fx, dq, tc, id, tm, mh, nm, kf, ox, px, rx, ui, sy]
230. ANTI-PARATHYROID.mp. [mp=ab, hw, ti, tn, ot, dm, mf, dv, kw, fx, dq, tc, id, tm, mh, nm, kf, ox, px, rx, ui, sy]
231. ANTIPARATHYROID.mp. [mp=ab, hw, ti, tn, ot, dm, mf, dv, kw, fx, dq, tc, id, tm, mh, nm, kf, ox, px, rx, ui, sy]
232. CALCITONIN*.mp. [mp=ab, hw, ti, tn, ot, dm, mf, dv, kw, fx, dq, tc, id, tm, mh, nm, kf, ox, px, rx, ui, sy]
233. TETRACYCLINE*.mp. [mp=ab, hw, ti, tn, ot, dm, mf, dv, kw, fx, dq, tc, id, tm, mh, nm, kf, ox, px, rx, ui, sy]
234. AMPHENICOL*.mp. [mp=ab, hw, ti, tn, ot, dm, mf, dv, kw, fx, dq, tc, id, tm, mh, nm, kf, ox, px, rx, ui, sy]
235. CHLORAMPHENICOL*.mp. [mp=ab, hw, ti, tn, ot, dm, mf, dv, kw, fx, dq, tc, id, tm, mh, nm, kf, ox, px, rx, ui, sy]
236. BETA-LACTAM ANTIBACTERIAL*.mp. [mp=ab, hw, ti, tn, ot, dm, mf, dv, kw, fx, dq, tc, id, tm, mh, nm, kf, ox, px, rx, ui, sy]
237. BETALACTAM ANTIBACTERIAL*.mp. [mp=ab, hw, ti, tn, ot, dm, mf, dv, kw, fx, dq, tc, id, tm, mh, nm, kf, ox, px, rx, ui, sy]
238. PENICILLIN*.mp. [mp=ab, hw, ti, tn, ot, dm, mf, dv, kw, fx, dq, tc, id, tm, mh, nm, kf, ox, px, rx, ui, sy]
239. CARBENICILLIN.mp. [mp=ab, hw, ti, tn, ot, dm, mf, dv, kw, fx, dq, tc, id, tm, mh, nm, kf, ox, px, rx, ui, sy]
240. CEPHALOSPORIN*.mp. [mp=ab, hw, ti, tn, ot, dm, mf, dv, kw, fx, dq, tc, id, tm, mh, nm, kf, ox, px, rx, ui, sy]
241. SULFONAMIDE*.mp. [mp=ab, hw, ti, tn, ot, dm, mf, dv, kw, fx, dq, tc, id, tm, mh, nm, kf, ox, px, rx, ui, sy]
242. TRIMETHOPRIM.mp. [mp=ab, hw, ti, tn, ot, dm, mf, dv, kw, fx, dq, tc, id, tm, mh, nm, kf, ox, px, rx, ui, sy]
243. MACROLIDE*.mp. [mp=ab, hw, ti, tn, ot, dm, mf, dv, kw, fx, dq, tc, id, tm, mh, nm, kf, ox, px, rx, ui, sy]
244. LINCOSAMIDE*.mp. [mp=ab, hw, ti, tn, ot, dm, mf, dv, kw, fx, dq, tc, id, tm, mh, nm, kf, ox, px, rx, ui, sy]
245. STREPTOGRAMIN*.mp. [mp=ab, hw, ti, tn, ot, dm, mf, dv, kw, fx, dq, tc, id, tm, mh, nm, kf, ox, px, rx, ui, sy]
246. AMINOGLYCOSIDE*.mp. [mp=ab, hw, ti, tn, ot, dm, mf, dv, kw, fx, dq, tc, id, tm, mh, nm, kf, ox, px, rx, ui, sy]
247. Streptomycin.mp. [mp=ab, hw, ti, tn, ot, dm, mf, dv, kw, fx, dq, tc, id, tm, mh, nm, kf, ox, px, rx, ui, sy]
248. QUINOLONE*.mp. [mp=ab, hw, ti, tn, ot, dm, mf, dv, kw, fx, dq, tc, id, tm, mh, nm, kf, ox, px, rx, ui, sy]

249. FLUOROQUINOLONE*.mp. [mp=ab, hw, ti, tn, ot, dm, mf, dv, kw, fx, dq, tc, id, tm, mh, nm, kf, ox, px, rx, ui, sy]
250. QUINOLONE*.mp. [mp=ab, hw, ti, tn, ot, dm, mf, dv, kw, fx, dq, tc, id, tm, mh, nm, kf, ox, px, rx, ui, sy]
251. CEPHALOSPORIN*.mp. [mp=ab, hw, ti, tn, ot, dm, mf, dv, kw, fx, dq, tc, id, tm, mh, nm, kf, ox, px, rx, ui, sy]
252. Monobactam*.mp. [mp=ab, hw, ti, tn, ot, dm, mf, dv, kw, fx, dq, tc, id, tm, mh, nm, kf, ox, px, rx, ui, sy]
253. carbapenem*.mp. [mp=ab, hw, ti, tn, ot, dm, mf, dv, kw, fx, dq, tc, id, tm, mh, nm, kf, ox, px, rx, ui, sy]
254. carbcephem*.mp. [mp=ab, hw, ti, tn, ot, dm, mf, dv, kw, fx, dq, tc, id, tm, mh, nm, kf, ox, px, rx, ui, sy]
255. imidazole.mp. [mp=ab, hw, ti, tn, ot, dm, mf, dv, kw, fx, dq, tc, id, tm, mh, nm, kf, ox, px, rx, ui, sy]
256. Pristinamycin.mp. [mp=ab, hw, ti, tn, ot, dm, mf, dv, kw, fx, dq, tc, id, tm, mh, nm, kf, ox, px, rx, ui, sy]
257. tigecyclin.mp. [mp=ab, hw, ti, tn, ot, dm, mf, dv, kw, fx, dq, tc, id, tm, mh, nm, kf, ox, px, rx, ui, sy]
258. ANTIMYCOTIC*.mp. [mp=ab, hw, ti, tn, ot, dm, mf, dv, kw, fx, dq, tc, id, tm, mh, nm, kf, ox, px, rx, ui, sy]
259. Terbinafine.mp. [mp=ab, hw, ti, tn, ot, dm, mf, dv, kw, fx, dq, tc, id, tm, mh, nm, kf, ox, px, rx, ui, sy]
260. Griseofulvin.mp. [mp=ab, hw, ti, tn, ot, dm, mf, dv, kw, fx, dq, tc, id, tm, mh, nm, kf, ox, px, rx, ui, sy]
261. ANTITUBERCULAR.mp. [mp=ab, hw, ti, tn, ot, dm, mf, dv, kw, fx, dq, tc, id, tm, mh, nm, kf, ox, px, rx, ui, sy]
262. Rifampicin.mp. [mp=ab, hw, ti, tn, ot, dm, mf, dv, kw, fx, dq, tc, id, tm, mh, nm, kf, ox, px, rx, ui, sy]
263. rifamycin.mp. [mp=ab, hw, ti, tn, ot, dm, mf, dv, kw, fx, dq, tc, id, tm, mh, nm, kf, ox, px, rx, ui, sy]
264. LEPRA TREATMENT*.mp. [mp=ab, hw, ti, tn, ot, dm, mf, dv, kw, fx, dq, tc, id, tm, mh, nm, kf, ox, px, rx, ui, sy]
265. LEPRA MEDICATION*.mp. [mp=ab, hw, ti, tn, ot, dm, mf, dv, kw, fx, dq, tc, id, tm, mh, nm, kf, ox, px, rx, ui, sy]
266. Thalidomide.mp. [mp=ab, hw, ti, tn, ot, dm, mf, dv, kw, fx, dq, tc, id, tm, mh, nm, kf, ox, px, rx, ui, sy]
267. ANTIVIRAL*.mp. [mp=ab, hw, ti, tn, ot, dm, mf, dv, kw, fx, dq, tc, id, tm, mh, nm, kf, ox, px, rx, ui, sy]
268. ribavirin.mp. [mp=ab, hw, ti, tn, ot, dm, mf, dv, kw, fx, dq, tc, id, tm, mh, nm, kf, ox, px, rx, ui, sy]
269. peginterferon.mp. [mp=ab, hw, ti, tn, ot, dm, mf, dv, kw, fx, dq, tc, id, tm, mh, nm, kf, ox, px, rx, ui, sy]
270. HIV ANTIVIRAL*.mp. [mp=ab, hw, ti, tn, ot, dm, mf, dv, kw, fx, dq, tc, id, tm, mh, nm, kf, ox, px, rx, ui, sy]
271. IMMUNE SERA.mp. [mp=ab, hw, ti, tn, ot, dm, mf, dv, kw, fx, dq, tc, id, tm, mh, nm, kf, ox, px, rx, ui, sy]
272. ANTITOXIC SERA.mp. [mp=ab, hw, ti, tn, ot, dm, mf, dv, kw, fx, dq, tc, id, tm, mh, nm, kf, ox, px, rx, ui, sy]
273. IMMUNOGLOBULIN*.mp. [mp=ab, hw, ti, tn, ot, dm, mf, dv, kw, fx, dq, tc, id, tm, mh, nm, kf, ox, px, rx, ui, sy]
274. POLYVALENT IMMUNOGLOBULIN*.mp. [mp=ab, hw, ti, tn, ot, dm, mf, dv, kw, fx, dq, tc, id, tm, mh, nm, kf, ox, px, rx, ui, sy]
275. ANTIBACTERIAL IMMUNOGLOBULIN*.mp. [mp=ab, hw, ti, tn, ot, dm, mf, dv, kw, fx, dq, tc, id, tm, mh, nm, kf, ox, px, rx, ui, sy]
276. ANTIVIRAL IMMUNOGLOBULIN*.mp. [mp=ab, hw, ti, tn, ot, dm, mf, dv, kw, fx, dq, tc, id, tm, mh, nm, kf, ox, px, rx, ui, sy]
277. BACTERIAL VACCINE*.mp. [mp=ab, hw, ti, tn, ot, dm, mf, dv, kw, fx, dq, tc, id, tm, mh, nm, kf, ox, px, rx, ui, sy]
278. VIRAL VACCINE*.mp. [mp=ab, hw, ti, tn, ot, dm, mf, dv, kw, fx, dq, tc, id, tm, mh, nm, kf, ox, px, rx, ui, sy]
279. VACCINE*.mp. [mp=ab, hw, ti, tn, ot, dm, mf, dv, kw, fx, dq, tc, id, tm, mh, nm, kf, ox, px, rx, ui, sy]
280. ANTINEOPLASTIC.mp. [mp=ab, hw, ti, tn, ot, dm, mf, dv, kw, fx, dq, tc, id, tm, mh, nm, kf, ox, px, rx, ui, sy]
281. CYTOSTATIC*.mp. [mp=ab, hw, ti, tn, ot, dm, mf, dv, kw, fx, dq, tc, id, tm, mh, nm, kf, ox, px, rx, ui, sy]

282. ALKYLATING AGENT*.mp. [mp=ab, hw, ti, tn, ot, dm, mf, dv, kw, fx, dq, tc, id, tm, mh, nm, kf, ox, px, rx, ui, sy]
283. ALKYLATING.mp. [mp=ab, hw, ti, tn, ot, dm, mf, dv, kw, fx, dq, tc, id, tm, mh, nm, kf, ox, px, rx, ui, sy]
284. Altretamine.mp. [mp=ab, hw, ti, tn, ot, dm, mf, dv, kw, fx, dq, tc, id, tm, mh, nm, kf, ox, px, rx, ui, sy]
285. ANTIMETABOLITE*.mp. [mp=ab, hw, ti, tn, ot, dm, mf, dv, kw, fx, dq, tc, id, tm, mh, nm, kf, ox, px, rx, ui, sy]
286. Methotrexate*.mp. [mp=ab, hw, ti, tn, ot, dm, mf, dv, kw, fx, dq, tc, id, tm, mh, nm, kf, ox, px, rx, ui, sy]
287. PLANT ALKALOID*.mp. [mp=ab, hw, ti, tn, ot, dm, mf, dv, kw, fx, dq, tc, id, tm, mh, nm, kf, ox, px, rx, ui, sy]
288. Irinotecan.mp. [mp=ab, hw, ti, tn, ot, dm, mf, dv, kw, fx, dq, tc, id, tm, mh, nm, kf, ox, px, rx, ui, sy]
289. topotecan.mp. [mp=ab, hw, ti, tn, ot, dm, mf, dv, kw, fx, dq, tc, id, tm, mh, nm, kf, ox, px, rx, ui, sy]
290. omacetaxine mepisuccinate.mp. [mp=ab, hw, ti, tn, ot, dm, mf, dv, kw, fx, dq, tc, id, tm, mh, nm, kf, ox, px, rx, ui, sy]
291. CYTOTOXIC ANTIBIOTIC*.mp. [mp=ab, hw, ti, tn, ot, dm, mf, dv, kw, fx, dq, tc, id, tm, mh, nm, kf, ox, px, rx, ui, sy]
292. CYTOSTATIC ANTINEOPLASTIC*.mp. [mp=ab, hw, ti, tn, ot, dm, mf, dv, kw, fx, dq, tc, id, tm, mh, nm, kf, ox, px, rx, ui, sy]
293. Gemtuzumab ozogamicin.mp. [mp=ab, hw, ti, tn, ot, dm, mf, dv, kw, fx, dq, tc, id, tm, mh, nm, kf, ox, px, rx, ui, sy]
294. Altretamine.mp. [mp=ab, hw, ti, tn, ot, dm, mf, dv, kw, fx, dq, tc, id, tm, mh, nm, kf, ox, px, rx, ui, sy]
295. Irinotecan.mp. [mp=ab, hw, ti, tn, ot, dm, mf, dv, kw, fx, dq, tc, id, tm, mh, nm, kf, ox, px, rx, ui, sy]
296. topotecan.mp. [mp=ab, hw, ti, tn, ot, dm, mf, dv, kw, fx, dq, tc, id, tm, mh, nm, kf, ox, px, rx, ui, sy]
297. Ibritumomab tiuxetan.mp. [mp=ab, hw, ti, tn, ot, dm, mf, dv, kw, fx, dq, tc, id, tm, mh, nm, kf, ox, px, rx, ui, sy]
298. tositumomab.mp. [mp=ab, hw, ti, tn, ot, dm, mf, dv, kw, fx, dq, tc, id, tm, mh, nm, kf, ox, px, rx, ui, sy]
299. PLATINUM ANTINEOPLASTIC*.mp. [mp=ab, hw, ti, tn, ot, dm, mf, dv, kw, fx, dq, tc, id, tm, mh, nm, kf, ox, px, rx, ui, sy]
300. MONOCLONAL ANTIBOD*.mp. [mp=ab, hw, ti, tn, ot, dm, mf, dv, kw, fx, dq, tc, id, tm, mh, nm, kf, ox, px, rx, ui, sy]
301. PROTEIN KINASE INHIBITOR*.mp. [mp=ab, hw, ti, tn, ot, dm, mf, dv, kw, fx, dq, tc, id, tm, mh, nm, kf, ox, px, rx, ui, sy]
302. CYTOSTATIC HORMONE*.mp. [mp=ab, hw, ti, tn, ot, dm, mf, dv, kw, fx, dq, tc, id, tm, mh, nm, kf, ox, px, rx, ui, sy]
303. CYTOSTATIC HORMONE ANTAGONIST*.mp. [mp=ab, hw, ti, tn, ot, dm, mf, dv, kw, fx, dq, tc, id, tm, mh, nm, kf, ox, px, rx, ui, sy]
304. IMMUNOSTIMULATING.mp. [mp=ab, hw, ti, tn, ot, dm, mf, dv, kw, fx, dq, tc, id, tm, mh, nm, kf, ox, px, rx, ui, sy]
305. MMUNOSTIMULANT*.mp. [mp=ab, hw, ti, tn, ot, dm, mf, dv, kw, fx, dq, tc, id, tm, mh, nm, kf, ox, px, rx, ui, sy]
306. Levamizole.mp. [mp=ab, hw, ti, tn, ot, dm, mf, dv, kw, fx, dq, tc, id, tm, mh, nm, kf, ox, px, rx, ui, sy]
307. INTERFERON*.mp. [mp=ab, hw, ti, tn, ot, dm, mf, dv, kw, fx, dq, tc, id, tm, mh, nm, kf, ox, px, rx, ui, sy]
308. IMMUNOSUPPRESSANT*.mp. [mp=ab, hw, ti, tn, ot, dm, mf, dv, kw, fx, dq, tc, id, tm, mh, nm, kf, ox, px, rx, ui, sy]
309. ANTI-TNF.mp. [mp=ab, hw, ti, tn, ot, dm, mf, dv, kw, fx, dq, tc, id, tm, mh, nm, kf, ox, px, rx, ui, sy]
310. INTERLEUKIN INHIBITOR*.mp. [mp=ab, hw, ti, tn, ot, dm, mf, dv, kw, fx, dq, tc, id, tm, mh, nm, kf, ox, px, rx, ui, sy]
311. Calcineurin inhibitor*.mp. [mp=ab, hw, ti, tn, ot, dm, mf, dv, kw, fx, dq, tc, id, tm, mh, nm, kf, ox, px, rx, ui, sy]

312. Thalidomide.mp. [mp=ab, hw, ti, tn, ot, dm, mf, dv, kw, fx, dq, tc, id, tm, mh, nm, kf, ox, px, rx, ui, sy]
313. ANTI RHEUMATIC.mp. [mp=ab, hw, ti, tn, ot, dm, mf, dv, kw, fx, dq, tc, id, tm, mh, nm, kf, ox, px, rx, ui, sy]
314. ANTIINFLAMMATORY.mp. [mp=ab, hw, ti, tn, ot, dm, mf, dv, kw, fx, dq, tc, id, tm, mh, nm, kf, ox, px, rx, ui, sy]
315. ANTI INFLAMMATORY.mp. [mp=ab, hw, ti, tn, ot, dm, mf, dv, kw, fx, dq, tc, id, tm, mh, nm, kf, ox, px, rx, ui, sy]
316. analgesic*.mp. [mp=ab, hw, ti, tn, ot, dm, mf, dv, kw, fx, dq, tc, id, tm, mh, nm, kf, ox, px, rx, ui, sy]
317. Chloroquine.mp. [mp=ab, hw, ti, tn, ot, dm, mf, dv, kw, fx, dq, tc, id, tm, mh, nm, kf, ox, px, rx, ui, sy]
318. Anakinra.mp. [mp=ab, hw, ti, tn, ot, dm, mf, dv, kw, fx, dq, tc, id, tm, mh, nm, kf, ox, px, rx, ui, sy]
319. abatacept.mp. [mp=ab, hw, ti, tn, ot, dm, mf, dv, kw, fx, dq, tc, id, tm, mh, nm, kf, ox, px, rx, ui, sy]
320. apremilast.mp. [mp=ab, hw, ti, tn, ot, dm, mf, dv, kw, fx, dq, tc, id, tm, mh, nm, kf, ox, px, rx, ui, sy]
321. leflunomide.mp. [mp=ab, hw, ti, tn, ot, dm, mf, dv, kw, fx, dq, tc, id, tm, mh, nm, kf, ox, px, rx, ui, sy]
322. tocilizumab.mp. [mp=ab, hw, ti, tn, ot, dm, mf, dv, kw, fx, dq, tc, id, tm, mh, nm, kf, ox, px, rx, ui, sy]
323. MUSCLE RELAXANT*.mp. [mp=ab, hw, ti, tn, ot, dm, mf, dv, kw, fx, dq, tc, id, tm, mh, nm, kf, ox, px, rx, ui, sy]
324. Dantrolene.mp. [mp=ab, hw, ti, tn, ot, dm, mf, dv, kw, fx, dq, tc, id, tm, mh, nm, kf, ox, px, rx, ui, sy]
325. Tolperisone.mp. [mp=ab, hw, ti, tn, ot, dm, mf, dv, kw, fx, dq, tc, id, tm, mh, nm, kf, ox, px, rx, ui, sy]
326. ANTI GOUT.mp. [mp=ab, hw, ti, tn, ot, dm, mf, dv, kw, fx, dq, tc, id, tm, mh, nm, kf, ox, px, rx, ui, sy]
327. ANTIGOUT.mp. [mp=ab, hw, ti, tn, ot, dm, mf, dv, kw, fx, dq, tc, id, tm, mh, nm, kf, ox, px, rx, ui, sy]
328. GOUT MEDICATION*.mp. [mp=ab, hw, ti, tn, ot, dm, mf, dv, kw, fx, dq, tc, id, tm, mh, nm, kf, ox, px, rx, ui, sy]
329. BONE CALCIUM REGULATOR*.mp. [mp=ab, hw, ti, tn, ot, dm, mf, dv, kw, fx, dq, tc, id, tm, mh, nm, kf, ox, px, rx, ui, sy]
330. Dibotermin.mp. [mp=ab, hw, ti, tn, ot, dm, mf, dv, kw, fx, dq, tc, id, tm, mh, nm, kf, ox, px, rx, ui, sy]
331. Eptotermin.mp. [mp=ab, hw, ti, tn, ot, dm, mf, dv, kw, fx, dq, tc, id, tm, mh, nm, kf, ox, px, rx, ui, sy]
332. Quinine.mp. [mp=ab, hw, ti, tn, ot, dm, mf, dv, kw, fx, dq, tc, id, tm, mh, nm, kf, ox, px, rx, ui, sy]
333. ANTI INFLAMMATORY ENZYME*.mp. [mp=ab, hw, ti, tn, ot, dm, mf, dv, kw, fx, dq, tc, id, tm, mh, nm, kf, ox, px, rx, ui, sy]
334. Bromelain*.mp. [mp=ab, hw, ti, tn, ot, dm, mf, dv, kw, fx, dq, tc, id, tm, mh, nm, kf, ox, px, rx, ui, sy]
335. Chymotrypsin.mp. [mp=ab, hw, ti, tn, ot, dm, mf, dv, kw, fx, dq, tc, id, tm, mh, nm, kf, ox, px, rx, ui, sy]
336. GENERAL ANAESTHETIC*.mp. [mp=ab, hw, ti, tn, ot, dm, mf, dv, kw, fx, dq, tc, id, tm, mh, nm, kf, ox, px, rx, ui, sy]
337. Phenoperidine.mp. [mp=ab, hw, ti, tn, ot, dm, mf, dv, kw, fx, dq, tc, id, tm, mh, nm, kf, ox, px, rx, ui, sy]
338. anileridine.mp. [mp=ab, hw, ti, tn, ot, dm, mf, dv, kw, fx, dq, tc, id, tm, mh, nm, kf, ox, px, rx, ui, sy]
339. Droperidol.mp. [mp=ab, hw, ti, tn, ot, dm, mf, dv, kw, fx, dq, tc, id, tm, mh, nm, kf, ox, px, rx, ui, sy]
340. OPIOID*.mp. [mp=ab, hw, ti, tn, ot, dm, mf, dv, kw, fx, dq, tc, id, tm, mh, nm, kf, ox, px, rx, ui, sy]
341. NARCOTIC*.mp. [mp=ab, hw, ti, tn, ot, dm, mf, dv, kw, fx, dq, tc, id, tm, mh, nm, kf, ox, px, rx, ui, sy]
342. Dihydrocodeine.mp. [mp=ab, hw, ti, tn, ot, dm, mf, dv, kw, fx, dq, tc, id, tm, mh, nm, kf, ox, px, rx, ui, sy]
343. Droperidol.mp. [mp=ab, hw, ti, tn, ot, dm, mf, dv, kw, fx, dq, tc, id, tm, mh, nm, kf, ox, px, rx, ui, sy]
344. ANALGESIC*.mp. [mp=ab, hw, ti, tn, ot, dm, mf, dv, kw, fx, dq, tc, id, tm, mh, nm, kf, ox, px, rx, ui, sy]
345. ANTI-PYRETIC*.mp. [mp=ab, hw, ti, tn, ot, dm, mf, dv, kw, fx, dq, tc, id, tm, mh, nm, kf, ox, px, rx, ui, sy]
346. Cannabinoid*.mp. [mp=ab, hw, ti, tn, ot, dm, mf, dv, kw, fx, dq, tc, id, tm, mh, nm, kf, ox, px, rx, ui, sy]

347. ANTIMIGRAINE.mp. [mp=ab, hw, ti, tn, ot, dm, mf, dv, kw, fx, dq, tc, id, tm, mh, nm, kf, ox, px, rx, ui, sy]
348. ANTI MIGRAINE.mp. [mp=ab, hw, ti, tn, ot, dm, mf, dv, kw, fx, dq, tc, id, tm, mh, nm, kf, ox, px, rx, ui, sy]
349. Dihydroergotamine.mp. [mp=ab, hw, ti, tn, ot, dm, mf, dv, kw, fx, dq, tc, id, tm, mh, nm, kf, ox, px, rx, ui, sy]
350. Pizotifen.mp. [mp=ab, hw, ti, tn, ot, dm, mf, dv, kw, fx, dq, tc, id, tm, mh, nm, kf, ox, px, rx, ui, sy]
351. ANTI-EPILEPTIC*.mp. [mp=ab, hw, ti, tn, ot, dm, mf, dv, kw, fx, dq, tc, id, tm, mh, nm, kf, ox, px, rx, ui, sy]
352. ANTIEPILEPTIC*.mp. [mp=ab, hw, ti, tn, ot, dm, mf, dv, kw, fx, dq, tc, id, tm, mh, nm, kf, ox, px, rx, ui, sy]
353. ANTICHOLINERGIC.mp. [mp=ab, hw, ti, tn, ot, dm, mf, dv, kw, fx, dq, tc, id, tm, mh, nm, kf, ox, px, rx, ui, sy]
354. DOPAMINERGIC.mp. [mp=ab, hw, ti, tn, ot, dm, mf, dv, kw, fx, dq, tc, id, tm, mh, nm, kf, ox, px, rx, ui, sy]
355. Antipsychotic*.mp. [mp=ab, hw, ti, tn, ot, dm, mf, dv, kw, fx, dq, tc, id, tm, mh, nm, kf, ox, px, rx, ui, sy]
356. Mebutamate.mp. [mp=ab, hw, ti, tn, ot, dm, mf, dv, kw, fx, dq, tc, id, tm, mh, nm, kf, ox, px, rx, ui, sy]
357. Mefenoxalone.mp. [mp=ab, hw, ti, tn, ot, dm, mf, dv, kw, fx, dq, tc, id, tm, mh, nm, kf, ox, px, rx, ui, sy]
358. ANXIOLYTIC*.mp. [mp=ab, hw, ti, tn, ot, dm, mf, dv, kw, fx, dq, tc, id, tm, mh, nm, kf, ox, px, rx, ui, sy]
359. HYPNOTIC*.mp. [mp=ab, hw, ti, tn, ot, dm, mf, dv, kw, fx, dq, tc, id, tm, mh, nm, kf, ox, px, rx, ui, sy]
360. SEDATIVE*.mp. [mp=ab, hw, ti, tn, ot, dm, mf, dv, kw, fx, dq, tc, id, tm, mh, nm, kf, ox, px, rx, ui, sy]
361. TRANQUILLISER*.mp. [mp=ab, hw, ti, tn, ot, dm, mf, dv, kw, fx, dq, tc, id, tm, mh, nm, kf, ox, px, rx, ui, sy]
362. Mebicar.mp. [mp=ab, hw, ti, tn, ot, dm, mf, dv, kw, fx, dq, tc, id, tm, mh, nm, kf, ox, px, rx, ui, sy]
363. Melatonin.mp. [mp=ab, hw, ti, tn, ot, dm, mf, dv, kw, fx, dq, tc, id, tm, mh, nm, kf, ox, px, rx, ui, sy]
364. ANTI-DEPRESSANT*.mp. [mp=ab, hw, ti, tn, ot, dm, mf, dv, kw, fx, dq, tc, id, tm, mh, nm, kf, ox, px, rx, ui, sy]
365. ANTIDEPRESSANT*.mp. [mp=ab, hw, ti, tn, ot, dm, mf, dv, kw, fx, dq, tc, id, tm, mh, nm, kf, ox, px, rx, ui, sy]
366. MOOD STABILISER*.mp. [mp=ab, hw, ti, tn, ot, dm, mf, dv, kw, fx, dq, tc, id, tm, mh, nm, kf, ox, px, rx, ui, sy]
367. Lithium.mp. [mp=ab, hw, ti, tn, ot, dm, mf, dv, kw, fx, dq, tc, id, tm, mh, nm, kf, ox, px, rx, ui, sy]
368. Ademetionine.mp. [mp=ab, hw, ti, tn, ot, dm, mf, dv, kw, fx, dq, tc, id, tm, mh, nm, kf, ox, px, rx, ui, sy]
369. Bupropion.mp. [mp=ab, hw, ti, tn, ot, dm, mf, dv, kw, fx, dq, tc, id, tm, mh, nm, kf, ox, px, rx, ui, sy]
370. PSYCHOSTIMULANT*.mp. [mp=ab, hw, ti, tn, ot, dm, mf, dv, kw, fx, dq, tc, id, tm, mh, nm, kf, ox, px, rx, ui, sy]
371. Dexamfetamine.mp. [mp=ab, hw, ti, tn, ot, dm, mf, dv, kw, fx, dq, tc, id, tm, mh, nm, kf, ox, px, rx, ui, sy]
372. metamfetamine.mp. [mp=ab, hw, ti, tn, ot, dm, mf, dv, kw, fx, dq, tc, id, tm, mh, nm, kf, ox, px, rx, ui, sy]
373. caffeine.mp. [mp=ab, hw, ti, tn, ot, dm, mf, dv, kw, fx, dq, tc, id, tm, mh, nm, kf, ox, px, rx, ui, sy]
374. Citicoline.mp. [mp=ab, hw, ti, tn, ot, dm, mf, dv, kw, fx, dq, tc, id, tm, mh, nm, kf, ox, px, rx, ui, sy]
375. Atomoxetine.mp. [mp=ab, hw, ti, tn, ot, dm, mf, dv, kw, fx, dq, tc, id, tm, mh, nm, kf, ox, px, rx, ui, sy]
376. OPIOID*.mp. [mp=ab, hw, ti, tn, ot, dm, mf, dv, kw, fx, dq, tc, id, tm, mh, nm, kf, ox, px, rx, ui, sy]
377. NARCOTIC*.mp. [mp=ab, hw, ti, tn, ot, dm, mf, dv, kw, fx, dq, tc, id, tm, mh, nm, kf, ox, px, rx, ui, sy]
378. Dihydrocodeine.mp. [mp=ab, hw, ti, tn, ot, dm, mf, dv, kw, fx, dq, tc, id, tm, mh, nm, kf, ox, px, rx, ui, sy]
379. Droperidol.mp. [mp=ab, hw, ti, tn, ot, dm, mf, dv, kw, fx, dq, tc, id, tm, mh, nm, kf, ox, px, rx, ui, sy]
380. ANALGESICS.mp. [mp=ab, hw, ti, tn, ot, dm, mf, dv, kw, fx, dq, tc, id, tm, mh, nm, kf, ox, px, rx, ui, sy]
381. ANALGESIC*.mp. [mp=ab, hw, ti, tn, ot, dm, mf, dv, kw, fx, dq, tc, id, tm, mh, nm, kf, ox, px, rx, ui, sy]
382. ANTI-PYRETIC*.mp. [mp=ab, hw, ti, tn, ot, dm, mf, dv, kw, fx, dq, tc, id, tm, mh, nm, kf, ox, px, rx, ui, sy]
383. Cannabinoid*.mp. [mp=ab, hw, ti, tn, ot, dm, mf, dv, kw, fx, dq, tc, id, tm, mh, nm, kf, ox, px, rx, ui, sy]

384. ANTIMIGRAINE.mp. [mp=ab, hw, ti, tn, ot, dm, mf, dv, kw, fx, dq, tc, id, tm, mh, nm, kf, ox, px, rx, ui, sy]
385. ANTI MIGRAINE.mp. [mp=ab, hw, ti, tn, ot, dm, mf, dv, kw, fx, dq, tc, id, tm, mh, nm, kf, ox, px, rx, ui, sy]
386. Dihydroergotamine.mp. [mp=ab, hw, ti, tn, ot, dm, mf, dv, kw, fx, dq, tc, id, tm, mh, nm, kf, ox, px, rx, ui, sy]
387. Pizotifen.mp. [mp=ab, hw, ti, tn, ot, dm, mf, dv, kw, fx, dq, tc, id, tm, mh, nm, kf, ox, px, rx, ui, sy]
388. ANTI-EPILEPTIC*.mp. [mp=ab, hw, ti, tn, ot, dm, mf, dv, kw, fx, dq, tc, id, tm, mh, nm, kf, ox, px, rx, ui, sy]
389. ANTIEPILEPTIC*.mp. [mp=ab, hw, ti, tn, ot, dm, mf, dv, kw, fx, dq, tc, id, tm, mh, nm, kf, ox, px, rx, ui, sy]
390. ANTICHOLINERGIC.mp. [mp=ab, hw, ti, tn, ot, dm, mf, dv, kw, fx, dq, tc, id, tm, mh, nm, kf, ox, px, rx, ui, sy]
391. DOPAMINERGIC.mp. [mp=ab, hw, ti, tn, ot, dm, mf, dv, kw, fx, dq, tc, id, tm, mh, nm, kf, ox, px, rx, ui, sy]
392. Antipsychotic*.mp. [mp=ab, hw, ti, tn, ot, dm, mf, dv, kw, fx, dq, tc, id, tm, mh, nm, kf, ox, px, rx, ui, sy]
393. Hypnotic*.mp. [mp=ab, hw, ti, tn, ot, dm, mf, dv, kw, fx, dq, tc, id, tm, mh, nm, kf, ox, px, rx, ui, sy]
394. Sedative*.mp. [mp=ab, hw, ti, tn, ot, dm, mf, dv, kw, fx, dq, tc, id, tm, mh, nm, kf, ox, px, rx, ui, sy]
395. Anxiolytic*.mp. [mp=ab, hw, ti, tn, ot, dm, mf, dv, kw, fx, dq, tc, id, tm, mh, nm, kf, ox, px, rx, ui, sy]
396. Tranquilizer*.mp. [mp=ab, hw, ti, tn, ot, dm, mf, dv, kw, fx, dq, tc, id, tm, mh, nm, kf, ox, px, rx, ui, sy]
397. Lithium.mp. [mp=ab, hw, ti, tn, ot, dm, mf, dv, kw, fx, dq, tc, id, tm, mh, nm, kf, ox, px, rx, ui, sy]
398. Mebutamate.mp. [mp=ab, hw, ti, tn, ot, dm, mf, dv, kw, fx, dq, tc, id, tm, mh, nm, kf, ox, px, rx, ui, sy]
399. Mefenoxalone.mp. [mp=ab, hw, ti, tn, ot, dm, mf, dv, kw, fx, dq, tc, id, tm, mh, nm, kf, ox, px, rx, ui, sy]
400. ANXIOLYTIC*.mp. [mp=ab, hw, ti, tn, ot, dm, mf, dv, kw, fx, dq, tc, id, tm, mh, nm, kf, ox, px, rx, ui, sy]
401. HYPNOTIC*.mp. [mp=ab, hw, ti, tn, ot, dm, mf, dv, kw, fx, dq, tc, id, tm, mh, nm, kf, ox, px, rx, ui, sy]
402. SEDATIVE*.mp. [mp=ab, hw, ti, tn, ot, dm, mf, dv, kw, fx, dq, tc, id, tm, mh, nm, kf, ox, px, rx, ui, sy]
403. TRANQUILLISER*.mp. [mp=ab, hw, ti, tn, ot, dm, mf, dv, kw, fx, dq, tc, id, tm, mh, nm, kf, ox, px, rx, ui, sy]
404. Mebicar.mp. [mp=ab, hw, ti, tn, ot, dm, mf, dv, kw, fx, dq, tc, id, tm, mh, nm, kf, ox, px, rx, ui, sy]
405. Melatonin.mp. [mp=ab, hw, ti, tn, ot, dm, mf, dv, kw, fx, dq, tc, id, tm, mh, nm, kf, ox, px, rx, ui, sy]
406. ANTI-DEPRESSANT*.mp. [mp=ab, hw, ti, tn, ot, dm, mf, dv, kw, fx, dq, tc, id, tm, mh, nm, kf, ox, px, rx, ui, sy]
407. ANTI-DEPRESSANT*.mp. [mp=ab, hw, ti, tn, ot, dm, mf, dv, kw, fx, dq, tc, id, tm, mh, nm, kf, ox, px, rx, ui, sy]
408. ANTIDEPRESSANT*.mp. [mp=ab, hw, ti, tn, ot, dm, mf, dv, kw, fx, dq, tc, id, tm, mh, nm, kf, ox, px, rx, ui, sy]
409. MOOD STABILISER*.mp. [mp=ab, hw, ti, tn, ot, dm, mf, dv, kw, fx, dq, tc, id, tm, mh, nm, kf, ox, px, rx, ui, sy]
410. Lithium.mp. [mp=ab, hw, ti, tn, ot, dm, mf, dv, kw, fx, dq, tc, id, tm, mh, nm, kf, ox, px, rx, ui, sy]
411. Ademetionine.mp. [mp=ab, hw, ti, tn, ot, dm, mf, dv, kw, fx, dq, tc, id, tm, mh, nm, kf, ox, px, rx, ui, sy]
412. Bupropion.mp. [mp=ab, hw, ti, tn, ot, dm, mf, dv, kw, fx, dq, tc, id, tm, mh, nm, kf, ox, px, rx, ui, sy]
413. PSYCHOSTIMULANT*.mp. [mp=ab, hw, ti, tn, ot, dm, mf, dv, kw, fx, dq, tc, id, tm, mh, nm, kf, ox, px, rx, ui, sy]
414. Dexamfetamine.mp. [mp=ab, hw, ti, tn, ot, dm, mf, dv, kw, fx, dq, tc, id, tm, mh, nm, kf, ox, px, rx, ui, sy]
415. metamfetamine.mp. [mp=ab, hw, ti, tn, ot, dm, mf, dv, kw, fx, dq, tc, id, tm, mh, nm, kf, ox, px, rx, ui, sy]
416. caffeine.mp. [mp=ab, hw, ti, tn, ot, dm, mf, dv, kw, fx, dq, tc, id, tm, mh, nm, kf, ox, px, rx, ui, sy]
417. Citicoline.mp. [mp=ab, hw, ti, tn, ot, dm, mf, dv, kw, fx, dq, tc, id, tm, mh, nm, kf, ox, px, rx, ui, sy]
418. Atomoxetine.mp. [mp=ab, hw, ti, tn, ot, dm, mf, dv, kw, fx, dq, tc, id, tm, mh, nm, kf, ox, px, rx, ui, sy]
419. NOOTROPIC*.mp. [mp=ab, hw, ti, tn, ot, dm, mf, dv, kw, fx, dq, tc, id, tm, mh, nm, kf, ox, px, rx, ui, sy]
420. NEUROTONIC*.mp. [mp=ab, hw, ti, tn, ot, dm, mf, dv, kw, fx, dq, tc, id, tm, mh, nm, kf, ox, px, rx, ui, sy]

421. PSYCHOLEPTIC.mp. [mp=ab, hw, ti, tn, ot, dm, mf, dv, kw, fx, dq, tc, id, tm, mh, nm, kf, ox, px, rx, ui, sy]
422. PSYCHOANALEPTIC.mp. [mp=ab, hw, ti, tn, ot, dm, mf, dv, kw, fx, dq, tc, id, tm, mh, nm, kf, ox, px, rx, ui, sy]
423. ANTI-DEMENTIA.mp. [mp=ab, hw, ti, tn, ot, dm, mf, dv, kw, fx, dq, tc, id, tm, mh, nm, kf, ox, px, rx, ui, sy]
424. ANTI-ALZHEIMER.mp. [mp=ab, hw, ti, tn, ot, dm, mf, dv, kw, fx, dq, tc, id, tm, mh, nm, kf, ox, px, rx, ui, sy]
425. ANTIALZHEIMER.mp. [mp=ab, hw, ti, tn, ot, dm, mf, dv, kw, fx, dq, tc, id, tm, mh, nm, kf, ox, px, rx, ui, sy]
426. Ipidacrine.mp. [mp=ab, hw, ti, tn, ot, dm, mf, dv, kw, fx, dq, tc, id, tm, mh, nm, kf, ox, px, rx, ui, sy]
427. ANTIDEMENTIA.mp. [mp=ab, hw, ti, tn, ot, dm, mf, dv, kw, fx, dq, tc, id, tm, mh, nm, kf, ox, px, rx, ui, sy]
428. PARASYMPATHOMIMETIC*.mp. [mp=ab, hw, ti, tn, ot, dm, mf, dv, kw, fx, dq, tc, id, tm, mh, nm, kf, ox, px, rx, ui, sy]
429. Cevimeline.mp. [mp=ab, hw, ti, tn, ot, dm, mf, dv, kw, fx, dq, tc, id, tm, mh, nm, kf, ox, px, rx, ui, sy]
430. Nalorphine.mp. [mp=ab, hw, ti, tn, ot, dm, mf, dv, kw, fx, dq, tc, id, tm, mh, nm, kf, ox, px, rx, ui, sy]
431. naltrexone.mp. [mp=ab, hw, ti, tn, ot, dm, mf, dv, kw, fx, dq, tc, id, tm, mh, nm, kf, ox, px, rx, ui, sy]
432. naloxone.mp. [mp=ab, hw, ti, tn, ot, dm, mf, dv, kw, fx, dq, tc, id, tm, mh, nm, kf, ox, px, rx, ui, sy]
433. flumazenil.mp. [mp=ab, hw, ti, tn, ot, dm, mf, dv, kw, fx, dq, tc, id, tm, mh, nm, kf, ox, px, rx, ui, sy]
434. ipidacrine.mp. [mp=ab, hw, ti, tn, ot, dm, mf, dv, kw, fx, dq, tc, id, tm, mh, nm, kf, ox, px, rx, ui, sy]
435. Alemtuzumab.mp. [mp=ab, hw, ti, tn, ot, dm, mf, dv, kw, fx, dq, tc, id, tm, mh, nm, kf, ox, px, rx, ui, sy]
436. DRUG* FOR ADDICTIVE DISORDER*.mp. [mp=ab, hw, ti, tn, ot, dm, mf, dv, kw, fx, dq, tc, id, tm, mh, nm, kf, ox, px, rx, ui, sy]
437. ANTISMOKING.mp. [mp=ab, hw, ti, tn, ot, dm, mf, dv, kw, fx, dq, tc, id, tm, mh, nm, kf, ox, px, rx, ui, sy]
438. Bupropion.mp. [mp=ab, hw, ti, tn, ot, dm, mf, dv, kw, fx, dq, tc, id, tm, mh, nm, kf, ox, px, rx, ui, sy]
439. DRUG* IN ALCOHOL DEPENDENCE.mp. [mp=ab, hw, ti, tn, ot, dm, mf, dv, kw, fx, dq, tc, id, tm, mh, nm, kf, ox, px, rx, ui, sy]
440. DRUG* FOR ALCOHOL DEPENDENCE.mp. [mp=ab, hw, ti, tn, ot, dm, mf, dv, kw, fx, dq, tc, id, tm, mh, nm, kf, ox, px, rx, ui, sy]
441. Nalmefene.mp. [mp=ab, hw, ti, tn, ot, dm, mf, dv, kw, fx, dq, tc, id, tm, mh, nm, kf, ox, px, rx, ui, sy]
442. DRUG* USED IN OPIOID DEPENDENCE.mp. [mp=ab, hw, ti, tn, ot, dm, mf, dv, kw, fx, dq, tc, id, tm, mh, nm, kf, ox, px, rx, ui, sy]
443. DRUG* FOR OPIOID DEPENDENCE.mp. [mp=ab, hw, ti, tn, ot, dm, mf, dv, kw, fx, dq, tc, id, tm, mh, nm, kf, ox, px, rx, ui, sy]
444. ANTIVERTIGO.mp. [mp=ab, hw, ti, tn, ot, dm, mf, dv, kw, fx, dq, tc, id, tm, mh, nm, kf, ox, px, rx, ui, sy]
445. ANTI VERTIGO.mp. [mp=ab, hw, ti, tn, ot, dm, mf, dv, kw, fx, dq, tc, id, tm, mh, nm, kf, ox, px, rx, ui, sy]
446. Sugammadex.mp. [mp=ab, hw, ti, tn, ot, dm, mf, dv, kw, fx, dq, tc, id, tm, mh, nm, kf, ox, px, rx, ui, sy]
447. Cannabinoid*.mp. [mp=ab, hw, ti, tn, ot, dm, mf, dv, kw, fx, dq, tc, id, tm, mh, nm, kf, ox, px, rx, ui, sy]
448. Alemtuzumab.mp. [mp=ab, hw, ti, tn, ot, dm, mf, dv, kw, fx, dq, tc, id, tm, mh, nm, kf, ox, px, rx, ui, sy]
449. fingolimod.mp. [mp=ab, hw, ti, tn, ot, dm, mf, dv, kw, fx, dq, tc, id, tm, mh, nm, kf, ox, px, rx, ui, sy]
450. terflunomide.mp. [mp=ab, hw, ti, tn, ot, dm, mf, dv, kw, fx, dq, tc, id, tm, mh, nm, kf, ox, px, rx, ui, sy]
451. AMOEBICIDE*.mp. [mp=ab, hw, ti, tn, ot, dm, mf, dv, kw, fx, dq, tc, id, tm, mh, nm, kf, ox, px, rx, ui, sy]
452. metronidazole.mp. [mp=ab, hw, ti, tn, ot, dm, mf, dv, kw, fx, dq, tc, id, tm, mh, nm, kf, ox, px, rx, ui, sy]
453. Clioquinol.mp. [mp=ab, hw, ti, tn, ot, dm, mf, dv, kw, fx, dq, tc, id, tm, mh, nm, kf, ox, px, rx, ui, sy]
454. chlorquinaldol.mp. [mp=ab, hw, ti, tn, ot, dm, mf, dv, kw, fx, dq, tc, id, tm, mh, nm, kf, ox, px, rx, ui, sy]
455. tilbroquinol.mp. [mp=ab, hw, ti, tn, ot, dm, mf, dv, kw, fx, dq, tc, id, tm, mh, nm, kf, ox, px, rx, ui, sy]
456. glycobiarsol.mp. [mp=ab, hw, ti, tn, ot, dm, mf, dv, kw, fx, dq, tc, id, tm, mh, nm, kf, ox, px, rx, ui, sy]

457. ANTI-MALARIAL*.mp. [mp=ab, hw, ti, tn, ot, dm, mf, dv, kw, fx, dq, tc, id, tm, mh, nm, kf, ox, px, rx, ui, sy]
458. ANTIMALARIAL*.mp. [mp=ab, hw, ti, tn, ot, dm, mf, dv, kw, fx, dq, tc, id, tm, mh, nm, kf, ox, px, rx, ui, sy]
459. Chloroquine.mp. [mp=ab, hw, ti, tn, ot, dm, mf, dv, kw, fx, dq, tc, id, tm, mh, nm, kf, ox, px, rx, ui, sy]
460. hydroxychloroquine.mp. [mp=ab, hw, ti, tn, ot, dm, mf, dv, kw, fx, dq, tc, id, tm, mh, nm, kf, ox, px, rx, ui, sy]
461. SCHISTOSOMICIDE*.mp. [mp=ab, hw, ti, tn, ot, dm, mf, dv, kw, fx, dq, tc, id, tm, mh, nm, kf, ox, px, rx, ui, sy]
462. ANTHELMINTIC*.mp. [mp=ab, hw, ti, tn, ot, dm, mf, dv, kw, fx, dq, tc, id, tm, mh, nm, kf, ox, px, rx, ui, sy]
463. Levamisole.mp. [mp=ab, hw, ti, tn, ot, dm, mf, dv, kw, fx, dq, tc, id, tm, mh, nm, kf, ox, px, rx, ui, sy]
464. Ivermectin.mp. [mp=ab, hw, ti, tn, ot, dm, mf, dv, kw, fx, dq, tc, id, tm, mh, nm, kf, ox, px, rx, ui, sy]
465. SYSTEMIC NASAL DECONGESTANT*.mp. [mp=ab, hw, ti, tn, ot, dm, mf, dv, kw, fx, dq, tc, id, tm, mh, nm, kf, ox, px, rx, ui, sy]
466. NASAL DECONGESTANT*.mp. [mp=ab, hw, ti, tn, ot, dm, mf, dv, kw, fx, dq, tc, id, tm, mh, nm, kf, ox, px, rx, ui, sy]
467. THROAT PREPARATION*.mp. [mp=ab, hw, ti, tn, ot, dm, mf, dv, kw, fx, dq, tc, id, tm, mh, nm, kf, ox, px, rx, ui, sy]
468. THROAT MEDICATION*.mp. [mp=ab, hw, ti, tn, ot, dm, mf, dv, kw, fx, dq, tc, id, tm, mh, nm, kf, ox, px, rx, ui, sy]
469. Nimesulide.mp. [mp=ab, hw, ti, tn, ot, dm, mf, dv, kw, fx, dq, tc, id, tm, mh, nm, kf, ox, px, rx, ui, sy]
470. ANTI-ASTHMA.mp. [mp=ab, hw, ti, tn, ot, dm, mf, dv, kw, fx, dq, tc, id, tm, mh, nm, kf, ox, px, rx, ui, sy]
471. ANTIASTHMA.mp. [mp=ab, hw, ti, tn, ot, dm, mf, dv, kw, fx, dq, tc, id, tm, mh, nm, kf, ox, px, rx, ui, sy]
472. DRUG* FOR OBSTRUCTIVE AIRWAY DISEASE*.mp. [mp=ab, hw, ti, tn, ot, dm, mf, dv, kw, fx, dq, tc, id, tm, mh, nm, kf, ox, px, rx, ui, sy]
473. OBSTRUCTIVE AIRWAY DISEASE* Medication.mp. [mp=ab, hw, ti, tn, ot, dm, mf, dv, kw, fx, dq, tc, id, tm, mh, nm, kf, ox, px, rx, ui, sy]
474. Alpha-1-proteinase inhibitor*.mp. [mp=ab, hw, ti, tn, ot, dm, mf, dv, kw, fx, dq, tc, id, tm, mh, nm, kf, ox, px, rx, ui, sy]
475. alfa 1 antitrypsin.mp. [mp=ab, hw, ti, tn, ot, dm, mf, dv, kw, fx, dq, tc, id, tm, mh, nm, kf, ox, px, rx, ui, sy]
476. Azelastine.mp. [mp=ab, hw, ti, tn, ot, dm, mf, dv, kw, fx, dq, tc, id, tm, mh, nm, kf, ox, px, rx, ui, sy]
477. Ketotifen.mp. [mp=ab, hw, ti, tn, ot, dm, mf, dv, kw, fx, dq, tc, id, tm, mh, nm, kf, ox, px, rx, ui, sy]
478. Terfenadine.mp. [mp=ab, hw, ti, tn, ot, dm, mf, dv, kw, fx, dq, tc, id, tm, mh, nm, kf, ox, px, rx, ui, sy]
479. (COUGH and COLD PREPARATION*).mp. [mp=ab, hw, ti, tn, ot, dm, mf, dv, kw, fx, dq, tc, id, tm, mh, nm, kf, ox, px, rx, ui, sy]
480. COUGH DRUG*.mp. [mp=ab, hw, ti, tn, ot, dm, mf, dv, kw, fx, dq, tc, id, tm, mh, nm, kf, ox, px, rx, ui, sy]
481. COUGH MEDICATION*.mp. [mp=ab, hw, ti, tn, ot, dm, mf, dv, kw, fx, dq, tc, id, tm, mh, nm, kf, ox, px, rx, ui, sy]
482. EXPECTORANT*.mp. [mp=ab, hw, ti, tn, ot, dm, mf, dv, kw, fx, dq, tc, id, tm, mh, nm, kf, ox, px, rx, ui, sy]
483. COUGH SUPPRESSANT*.mp. [mp=ab, hw, ti, tn, ot, dm, mf, dv, kw, fx, dq, tc, id, tm, mh, nm, kf, ox, px, rx, ui, sy]
484. ANTITUSSIVE*.mp. [mp=ab, hw, ti, tn, ot, dm, mf, dv, kw, fx, dq, tc, id, tm, mh, nm, kf, ox, px, rx, ui, sy]
485. ANTIHISTAMINE*.mp. [mp=ab, hw, ti, tn, ot, dm, mf, dv, kw, fx, dq, tc, id, tm, mh, nm, kf, ox, px, rx, ui, sy]
486. Cyproheptadine.mp. [mp=ab, hw, ti, tn, ot, dm, mf, dv, kw, fx, dq, tc, id, tm, mh, nm, kf, ox, px, rx, ui, sy]
487. Thiethylperazine.mp. [mp=ab, hw, ti, tn, ot, dm, mf, dv, kw, fx, dq, tc, id, tm, mh, nm, kf, ox, px, rx, ui, sy]
488. exp Alzheimers disease/ or exp Dementia/ or dementia.mp.
489. exp Cognition disorders/ or exp Dementia/ or cognitive impairment.mp.

490.	exp HIV associated dementia/ or exp semantic dementia/ or exp senile dementia/ or dementia.mp. or exp multiinfarct dementia/ or exp frontal variant frontotemporal dementia/ or exp dementia/ or exp presenile dementia/ or exp frontotemporal dementia/
491.	cognitive impairment.mp. or exp cognitive defect/
492.	exp Vascular Dementia/ or exp Dementia/ or dementia.mp. or exp Dementia with Lewy Bodies/
493.	cognitive impairment.mp. or exp Cognitive Impairment/
494.	dementia.mp. or exp Dementia/ or exp Frontotemporal Dementia/ or exp Dementia, Vascular/ or exp Dementia, Multi-Infarct/
495.	cognitive impairment.mp. or exp Cognitive Dysfunction/
496.	1 or 2 or 3 or 4 or 5 or 6 or 7 or 8 or 9 or 10 or 11 or 12 or 13 or 14 or 15 or 16 or 17 or 18 or 19 or 20 or 21 or 22 or 23 or 24 or 25 or 26 or 27 or 28 or 29 or 30 or 31 or 32 or 33 or 34 or 35 or 36 or 37 or 38 or 39 or 40 or 41 or 42 or 43 or 44 or 45 or 46 or 47 or 48 or 49 or 50 or 51 or 52 or 53 or 54 or 55 or 56 or 57 or 58 or 59 or 60 or 61 or 62 or 63 or 64 or 65 or 66 or 67 or 68 or 69 or 70 or 71 or 72 or 73 or 74 or 75 or 76 or 77 or 78 or 79 or 80 or 81 or 82 or 83 or 84 or 85 or 86 or 87 or 88 or 89 or 90 or 91 or 92 or 93 or 94 or 95 or 96 or 97 or 98 or 99 or 100 or 101 or 102 or 103 or 104 or 105 or 106 or 107 or 108 or 109 or 110 or 111 or 112 or 113 or 114 or 115 or 116 or 117 or 118 or 119 or 120 or 121 or 122 or 123 or 124 or 125 or 126 or 127 or 128 or 129 or 130 or 131 or 132 or 133 or 134 or 135 or 136 or 137 or 138 or 139 or 140 or 141 or 142 or 143 or 144 or 145 or 146 or 147 or 148 or 149 or 150 or 151 or 152 or 153 or 154 or 155 or 156 or 157 or 158 or 159 or 160 or 161 or 162 or 163 or 164 or 165 or 166 or 167 or 168 or 169 or 170 or 171 or 172 or 173 or 174 or 175 or 176 or 177 or 178 or 179 or 180 or 181 or 182 or 183 or 184 or 185 or 186 or 187 or 188 or 189 or 190 or 191 or 192 or 193 or 194 or 195 or 196 or 197 or 198 or 199 or 200 or 201 or 202 or 203 or 204 or 205 or 206 or 207 or 208 or 209 or 210 or 211 or 212 or 213 or 214 or 215 or 216 or 217 or 218 or 219 or 220 or 221 or 222 or 223 or 224 or 225 or 226 or 227 or 228 or 229 or 230 or 231 or 232 or 233 or 234 or 235 or 236 or 237 or 238 or 239 or 240 or 241 or 242 or 243 or 244 or 245 or 246 or 247 or 248 or 249 or 250 or 251 or 252 or 253 or 254 or 255 or 256 or 257 or 258 or 259 or 260 or 261 or 262 or 263 or 264 or 265 or 266 or 267 or 268 or 269 or 270 or 271 or 272 or 273 or 274 or 275 or 276 or 277 or 278 or 279 or 280 or 281 or 282 or 283 or 284 or 285 or 286 or 287 or 288 or 289 or 290 or 291 or 292 or 293 or 294 or 295 or 296 or 297 or 298 or 299 or 300 or 301 or 302 or 303 or 304 or 305 or 306 or 307 or 308 or 309 or 310 or 311 or 312 or 313 or 314 or 315 or 316 or 317 or 318 or 319 or 320 or 321 or 322 or 323 or 324 or 325 or 326 or 327 or 328 or 329 or 330 or 331 or 332 or 333 or 334 or 335 or 336 or 337 or 338 or 339 or 340 or 341 or 342 or 343 or 344 or 345 or 346 or 347 or 348 or 349 or 350 or 351 or 352 or 353 or 354 or 355 or 356 or 357 or 358 or 359 or 360 or 361 or 362 or 363 or 364 or 365 or 366 or 367 or 368 or 369 or 370 or 371 or 372 or 373 or 374 or 375 or 376 or 377 or 378 or 379 or 380 or 381 or 382 or 383 or 384 or 385 or 386 or 387 or 388 or 389 or 390 or 391 or 392 or 393 or 394 or 395 or 396 or 397 or 398 or 399 or 400 or 401 or 402 or 403 or 404 or 405 or 406 or 407 or 408 or 409 or 410 or 411 or 412 or 413 or 414 or 415 or 416 or 417 or 418 or 419 or 420 or 421 or 422 or 423 or 424 or 425 or 426 or 427 or 428 or 429 or 430 or 431 or 432 or 433 or 434 or 435 or 436 or 437 or 438 or 439 or 440 or 441 or 442 or 443 or 444 or 445 or 446 or 447 or 448 or 449 or 450 or 451 or 452 or 453 or 454 or 455 or 456 or 457 or 458 or 459 or 460 or 461 or 462 or 463 or 464 or 465 or 466 or 467 or 468 or 469 or 470 or 471 or 472 or 473 or 474 or 475 or 476 or 477 or 478 or 479 or 480 or 481 or 482 or 483 or 484 or 485 or 486 or 487
497.	488 or 489 or 490 or 491 or 492 or 493 or 494 or 495
498.	496 and 497
499.	limit 498 to "systematic review"
