# Supplementary material for: Systemic medications and dementia risk: a systematic umbrella review
Source: Mol Psychiatry. 2025 Jul 24;30(11):5578–99. doi: 10.1038/s41380-025-03129-3 (PMC12532590; doi:10.1038/s41380-025-03129-3)
Supplement: Supplementary file 3 — 3: Full AMSTAR2 quality ratings [file 41380_2025_3129_MOESM3_ESM.docx]

**Supplement 3 : AMSTAR-2 COMPONENTS AND QUALITY RATINGS**

Table: AMSTAR-2 quality ratings. GREEN= critical components. BLUE= non-critical components. 1= Yes or Partial yes. 0= No.

*Detailed description of components:*

**Critical components:**

B. The review contains an explicit statement that the review methods were established prior to the conduct of the review, the review is registered, and did the report justify any significant deviations from the protocol?

D. Review authors use a comprehensive literature search strategy?

G. Review authors provide a list of excluded studies and justify the exclusions?

I. Review authors use a satisfactory technique for assessing the risk of bias in individual studies that were included in the review?

K. If meta-analysis was performed did the review authors use appropriate methods for statistical combination of results?

M. Review authors account for RoB in individual studies when interpreting/ discussing the results of the review?

O. If they performed quantitative synthesis did the review authors carry out an adequate investigation of publication bias (small study bias) and discuss its likely impact on the results of the review?

**Non-critical components:**

A. Research questions and inclusion criteria for the review include the components of PICO?

C. Authors explain their selection of the study designs for inclusion in the review?

E. Review authors perform study selection in duplicate?

F. Review authors perform data extraction in duplicate?

H. Review authors describe the included studies in adequate detail?

J. Review authors report on the sources of funding for the studies included in the review?

L. If meta-analysis was performed, did the review authors assess the potential impact of RoB in individual studies on the results of the meta-analysis or other evidence synthesis?

N. Review authors provide a satisfactory explanation for, and discussion of, any heterogeneity observed in the results of the review?

P. Review authors report any potential sources of conflict of interest, including any funding they received
