# Supplementary material for: Systemic medications and dementia risk: a systematic umbrella review
Source: Mol Psychiatry. 2025 Jul 24;30(11):5578–99. doi: 10.1038/s41380-025-03129-3 (PMC12532590; doi:10.1038/s41380-025-03129-3)
Supplement: Supplementary file 4 — 4: Citation matrices representing study overlap between systematic reviews [file 41380_2025_3129_MOESM4_ESM.docx]

**Supplement 4 : citation matrices**

Table 1: Antihypertensives

|  | Adesuyan et al, 2022 | Peters et al 2022 | Hughes et al 2020 | Ou et al 2020 | Xu et al 2017 | Cunningham et al 2021 | Ye et al 2015 | Hussain et al 2018 | Peters et al 2020 | Chang-Quan et al 2011 | Ding et al 2020 | Parsons et al, 2016 | Levi-Marpillat et al, 2013 | Peters et al, 2014 | McGuinness et al, 2009 | Zhuang et al, 2016 | Lennon et al 2023 |
| --- | --- | --- | --- | --- | --- | --- | --- | --- | --- | --- | --- | --- | --- | --- | --- | --- | --- |
| Applegate WB, Pressel S, Wittes J, et al. Impact of the treatment of isolated systolic hypertension on behavioral variables: results from the systolic hypertension in the elderly program (SHEP). Arch Intern Med. 1994;154(19):2154-2160. |  |  | x |  |  |  |  |  |  |  |  |  |  |  |  |  |  |
| Anstey KJ, Christensen H, Butterworth P, et al. Cohort profile: the PATH through life project. Int J Epidemiol. 2012;41(4):951-960. doi:10.1093/ije/dyr025 |  |  |  |  |  |  |  |  |  |  |  |  |  |  |  |  | x |
| Australian Longitudinal Study of Aging (ALSA) |  |  |  |  |  |  |  |  | x |  |  |  |  |  |  |  |  |
| C. Qiu, E. von Strauss, B. Winblad, and L. Fratiglioni, “Decline in Blood PressureOver Time and Risk of Dementia: A Longitudinal Study From the Kungsholmen Project,” Stroke, vol. 35, no. 8, pp. 1810–1815, 2004. |  |  |  | x | x |  |  |  |  | x |  |  |  |  |  |  |  |
| Canadian Study of Health and Ageing (CSHA). Lindsay J, Laurin D, Verreault R, Hebert R, Helliwell B, Hill GB, McDowell I. Risk factors for Alzheimer’s disease: a prospective analysis from the Canadian Study of Health and Aging. Am J Epidemiol 2002; 156:445–453. |  |  |  |  |  |  |  |  | x |  |  |  | x |  |  |  |  |
| Cognitive Function and Ageing Study I (CFAS I) |  |  |  |  |  |  |  |  | x |  |  |  |  |  |  |  |  |
| Cognitive Function and Ageing Study II (CFAS II) |  |  |  |  |  |  |  |  | x |  |  |  |  |  |  |  |  |
| Counts | 1 | 1 | 2 | 2 | 2 | 1 | 1 | 1 | 5 | 2 | 1 | 1 | 2 | 1 | 1 | 1 |  |
| D. G. Bruce, W. A. Davis, G. P. Casey et al., “Predictors of cognitive impairment and dementia in older people with diabetes,” Diabetologia, vol. 51, no. 2, pp. 241–248, 2008. (freemantle diabetes study) |  |  |  |  | x |  |  |  |  | x |  |  |  |  |  |  |  |
| Dardiotis E, Kosmidis MH, Yannakoulia M, Hadjigeorgiou GM, Scarmeas N. The Hellenic Longitudinal Investigation of Aging and Diet (HELIAD): rationale, study design, and cohort description. Neuroepidemiology. 2014;43(1):9-14. doi:10.1159/000362723 |  |  |  |  |  |  |  |  |  |  |  |  |  |  |  |  | x |
| Davies NM, Kehoe PG, Ben-Shlomo Y, Martin RM. Associations of anti-hypertensive treatments with Alzheimer's disease, vascular dementia, and other dementias. J Alzheimers Dis. 2011;26(4):699-708. doi: 10.3233/JAD-2011-110347. PMID: 21709373. (uk GPRD) |  |  |  |  |  |  | x |  |  |  |  |  |  |  |  | x |  |
| De Galan BE, Zoungas S, Chalmers J, et al; ADVANCE Collaborative Group. Cognitive function and risks of cardiovascular disease and hypoglycaemia in patients with type 2 diabetes: the Action in Diabetes and Vascular Disease: Preterax and Diamicron Modified Release Controlled Evaluation (ADVANCE) trial. Diabetologia. 2009;52 (11):2328-2336. doi:10.1007/s00125-009-1484-7 |  |  | x |  |  |  |  |  |  |  |  |  |  |  |  |  |  |
| Feldman L, Vinker S, Efrati S, Beberashvili I, Gorelik O, Wasser W, Shani M. Amlodipine treatment of hypertension associates with a decreased dementia risk. Clin Exp Hypertens. 2016;38(6):545-9. doi: 10.3109/10641963.2016.1174249. Epub 2016 Jul 8. PMID: 27392121. |  |  |  |  |  |  |  | x |  |  |  |  |  |  |  |  |  |
| Feng L, Gwee X, Kua EH, Ng TP. Cognitive function and tea consumption in community dwelling older Chinese in Singapore. J Nutr Health Aging. 2010;14(6):433-438. doi:10.1007/s12603-010-0095-9 |  |  |  |  |  |  |  |  |  |  |  |  |  |  |  |  | x |
| Forette F, Seux M, Staessen J, et al; Systolic Hypertension in Europe Investigators. Syst-eur The prevention of dementia with antihypertensive treatment. Arch Intern Med 2002; 162:2046–2052. |  | x | x | x |  |  |  | x | x |  |  |  |  | x |  |  |  |
| Forette F, Seux ML, Staessen JA et al. Prevention of dementia in randomised double-blind placebo-controlled Systolic Hypertension in Europe (Syst-Eur) trial. Lancet 352(9137), 1347–1351 (1998). |  | x |  |  |  | x |  |  |  | x |  | x | x |  | x |  |  |
| G. Wagner, A. Icks, H.-H. Abholz, D. Schröder-Bernhardi, W. Rathmann, K.Kostev, Antihypertensive treatment and risk of dementia: a retrospectivedatabase study, Int. J. Clin. Pharmacol. Ther. 50 (2012) 195–201. |  |  |  |  |  |  |  |  |  |  |  |  |  |  |  | x |  |
| Gottesman RF, Albert MS, Alonso A, et al. Associations between midlife vascular risk factors and 25-year incident dementia in the atherosclerosis risk in communities (ARIC) cohort. JAMA Neurol 2017; 74: 1246–54. |  |  |  |  |  |  |  |  |  |  | x |  |  |  |  |  |  |
| Group CS. Vascular factors and risk of dementia: design of the Three-City Study and baseline characteristics of the study population. Neuroepidemiology 2003; 22: 316–25. |  |  |  |  |  |  |  |  |  |  | x |  |  |  |  |  |  |
| GuerchetM, Mbelesso P, Ndamba-Bandzouzi B, et al; EPIDEMCA group. Epidemiology of dementia in Central Africa (EPIDEMCA): protocol for a multicentre population-based study in rural and urban areas of the Central African Republic and the Republic of Congo. Springerplus. 2014;3(1):338. doi:10.1186/2193-1801-3-338 |  |  |  |  |  |  |  |  |  |  |  |  |  |  |  |  | x |
| Guo Z, Fratiglioni L, Zhu L, Fastbom J, Winblad B, Viitanen M. Occurrence and progression of dementia in a community population aged 75 years and older: relationship of antihypertensive medication use. Arch Neurol 1999; 56:991–996. Kungsholmen cohort |  |  |  |  |  |  |  |  |  |  |  |  | x |  |  |  |  |
| Haag MDM, Hofman A, Koudstaal PJ, Breteler MMB, Stricker BHC. Duration of antihypertensive drug use and risk of dementia A prospective cohort study. Neurology. 2009;72(20):1727-34. doi: 10.1212/01.wnl.0000345062.86148.3f (the rotterdam study) | x |  |  | x |  |  |  | x |  |  |  |  |  |  |  |  |  |
| Haan MN, Mungas DM, Gonzalez HM, Ortiz TA, Acharya A, JagustWJ. Prevalence of dementia in older latinos: the influence of type 2 diabetes mellitus, stroke and genetic factors. J AmGeriatr Soc. 2003;51(2):169-177. doi:10. 1046/j.1532-5415.2003.51054.x |  |  |  |  |  |  |  |  |  |  |  |  |  |  |  |  | x |
| Hall K, Gureje O, Gao S, et al. Risk factors and Alzheimer’s disease: a comparative study of two communities. Aust N Z J Psychiatry. 1998;32(5):698-706. doi:10.3109/00048679809113126 |  |  |  |  |  |  |  |  |  |  |  |  |  |  |  |  | x |
| Han JW, Kim TH, Kwak KP, et al. Overview of the Korean longitudinal study on cognitive aging and dementia. Psychiatry Investig. 2018;15(8):767-774. doi:10.30773/pi.2018.06.02 |  |  |  |  |  |  |  |  |  |  |  |  |  |  |  |  | x |
| Haring B, Wu C, Coker LH, Seth A, Snetselaar L, Manson JE, Rossouw JE, Wassertheil-Smoller S. Hypertension, Dietary Sodium, and Cognitive Decline: Results From the Women's Health Initiative Memory Study. Am J Hypertens. 2016 Feb;29(2):202-16. doi: 10.1093/ajh/hpv081. Epub 2015 Jul 1. PMID: 26137952; PMCID: PMC4723668. (WHIMS) |  |  |  | x | x |  |  |  |  |  |  |  |  |  |  |  |  |
| Harris TB, Launer LJ, Eiriksdottir G, et al. Age, gene/environment susceptibility-Reykjavik study: multidisciplinary applied phenomics. Am J Epidemiol 2007; 165: 1076–87. |  |  |  |  |  |  |  |  |  |  | x |  |  |  |  |  |  |
| Hendrie HC, Ogunniyi A, Hall KS, et al. Incidence of dementia and Alzheimer disease in 2 communities: Yoruba residing in Ibadan, Nigeria, and African Americans residing in Indianapolis, Indiana. JAMA. 2001;285(6):739-747. doi:10.1001/jama.285.6.739 |  |  |  |  |  |  |  |  |  |  |  |  |  |  |  |  | x |
| Hsu CY, Huang CC, Chan WL et al. Angiotensinreceptor blockers and risk of Alzheimer’s disease in hypertension population – a nationwide cohort study. Circ J 2013; 77: 405–10. (taiwan) |  |  |  |  |  |  | x |  |  |  |  |  |  |  |  |  |  |
| Hwang D, Kim S, Choi H, Oh IH, Kim BS, Choi HR, Kim SY, Won CW. Calcium-Channel Blockers and Dementia Risk in Older Adults　- National Health Insurance Service - Senior Cohort (2002-2013). Circ J. 2016 Oct 25;80(11):2336-2342. doi: 10.1253/circj.CJ-16-0692. Epub 2016 Sep 21. PMID: 27666598. Korean National Health Insurance Service |  |  |  |  |  |  |  | x |  |  |  |  |  |  |  |  |  |
| Ikram MA, Brusselle GGO, Murad SD, et al. The Rotterdam Study: 2018 update on objectives, design and main results. Eur J Epidemiol 2017; 32: 807–50 |  |  |  |  |  |  |  |  |  |  | x |  |  |  |  |  |  |
| in’t Veld BA, Ruitenberg A, Hofman A, Stricker BHC, Breteler MMB. Antihypertensive drugs and incidence of dementia: the Rotterdam Study. Neurobiol Aging. 2001;22(3):407-12. doi: 10.1016/s0197-4580(00)00241-4 (rotterdam study) | x |  |  | x | x |  |  |  |  | x |  |  | x |  |  |  |  |
| Khachaturian AS, Zandi PP, Lyketsos CG et al. Antihypertensive medication use and incident Alzheimer disease: the Cache County Study. Arch Neurol 2006; 63: 686–92. |  |  |  |  |  |  | x | x |  |  |  |  | x | x |  |  |  |
| Li NC, Lee A, Whitmer RA, Kivipelto M, Lawler E, Kazis LE, Wolozin B. Use of angiotensin receptor blockers and risk of dementia in a predominantly male population: prospective cohort analysis. BMJ. 2010 Jan 12;340:b5465. doi: 10.1136/bmj.b5465. PMID: 20068258; PMCID: PMC2806632. US VA |  |  |  |  |  |  | x |  |  |  |  |  | x |  |  | x |  |
| Lithell H, Hansson L, Skoog I et al. The Study on Cognition and Prognosis in the Elderly (SCOPE): principal results of a randomized double-blind intervention trial. J. Hypertens. 21(5), 875–886 (2003). |  |  | x |  |  | x |  |  |  |  |  | x | x |  | x | x |  |
| Lobo A, Saz P, Marcos G, et al. The ZARADEMP Project on the incidence, prevalence and risk factors of dementia (and depression) in the elderly community: I: the context and the objectives. Eur J Psychiatry. 2005;19 (1):31-39. doi:10.4321/S0213-61632005000100003 |  |  |  |  |  |  |  |  |  |  |  |  |  |  |  |  | x |
| Louis ED, Benito-León J, Bermejo-Pareja F; Neurological Disorders in Central Spain (NEDICES) Study Group. Antihypertensive agents and risk of Parkinson's disease, essential tremor and dementia: a population-based prospective study (NEDICES). Neuroepidemiology. 2009;33(3):286-92. doi: 10.1159/000235641. Epub 2009 Aug 20. PMID: 19696520; PMCID: PMC2826448. |  |  |  | x |  |  |  |  |  |  |  |  |  |  |  |  |  |
| M.L. Johnson, N. Parikh, M.E. Kunik, P.E. Schulz, J.G. Patel, H. Chen, R.R.Aparasu, R.O. Morgan, Antihypertensive drug use and the risk of dementia inpatients with diabetes mellitus, Alzheimer’s Dement. 8 (2012) 437–444. |  |  |  |  |  |  |  |  |  |  |  |  |  |  |  | x |  |
| M.L. Johnson, N. Parikh, M.E. Kunik, P.E. Schulz, J.G. Patel, H. Chen, R.R.Aparasu, R.O. Morgan, Antihypertensive drug use and the risk of dementia inpatients with diabetes mellitus, Alzheimer’s Dement. 8 (2012) 437–444. US VA |  |  |  |  |  |  |  |  |  |  |  |  |  |  |  | x |  |
| Murray MD, Hendrie HC, Lane KA, Zheng M, Ambuehl R, Li S, Unverzagt FW, Callahan CM, Gao S. Antihypertensive Medication and Dementia Risk in Older Adult African Americans with Hypertension: A Prospective Cohort Study. J Gen Intern Med. 2018 Apr;33(4):455-462. doi: 10.1007/s11606-017-4281-x. Epub 2018 Jan 12. PMID: 29330643; PMCID: PMC5880772. |  |  |  | x |  |  |  |  |  |  |  |  |  |  |  |  |  |
| Ohrui T, Matsui T, Yamaya M et al. Angiotensinconverting enzyme inhibitors and incidence of Alzheimer’s disease in Japan. J Am Geriatr Soc 2004; 52: 649–50. |  |  |  |  |  |  | x |  |  |  |  |  |  |  |  |  |  |
| Patel A, MacMahon S, Chalmers J, Neal B, Woodward M, Billot L, et al. Effects of a fixed combination of perindopril and indapamide on macrovascular and microvascular outcomes in patients with type 2 diabetes mellitus (the ADVANCE trial): a randomised controlled trial. Lancet 2007;370:829–840. |  | x |  |  |  |  |  |  |  |  |  |  |  |  |  |  |  |
| Peila R, White LR, Masaki K, Petrovitch H, Launer LJ. Reducing the risk of dementia: efficacy of long-term treatment of hypertension. Stroke. 2006 May;37(5):1165-70. doi: 10.1161/01.STR.0000217653.01615.93. Epub 2006 Apr 6. PMID: 16601212. (HAAS) |  |  |  | x |  |  |  |  |  |  |  |  |  |  |  |  |  |
| Peters R, Beckett N, Forette F et al. Incident dementia and blood pressure lowering in the Hypertension in the Very Elderly Trial cognitive function assessment (HYVETCOG): a double-blind, placebo controlled trial. Lancet Neurol. 7(8), 683–689 (2008). |  | x | x |  |  | x |  |  | x | x |  | x | x |  |  |  |  |
| Prevention of Dementia by Intensive Vascular Care (PreDIVA). Moll van Charante EP, Richard E, Eurelings LS, et al. Effectiveness of a 6-year multidomain vascular care intervention to prevent dementia (preDIVA): a clusterrandomised controlled trial. Lancet 2016;388:797–805. |  |  |  |  |  |  |  |  | x |  |  |  |  |  |  |  |  |
| Prevention of stroke by antihypertensive drug treatment in older persons with isolated systolic hypertension. Final results of the Systolic Hypertension in the Elderly Program (SHEP). SHEP Cooperative Research Group. JAMA 265(24), 3255–3264 (1991). |  | x |  |  |  | x |  |  | x |  |  | x | x |  | x |  |  |
| profess trial Effects of aspirin plus extended-release dipyridamole versus clopidogrel andtelmisartan on disability and cognitive function after recurrent stroke inpatients with ischaemic stroke in the Prevention Regimen for EffectivelyAvoiding Second Strokes (PRoFESS) trial: a double-blind, active andplacebo-controlled study, Lancet Neurol. 7 (2008) 875–884.l. |  |  |  |  |  |  |  |  |  |  |  |  |  |  |  | x |  |
| Qiu WQ, Mwamburi M, Besser LM et al. Angiotensin converting enzyme inhibitors and the reduced risk of Alzheimer’s disease in the absence of apolipoprotein E4 allele. J Alzheimers Dis 2013; 37: 421–8. National Alzheimer’s Disease Coordinating Center NACC |  |  |  |  |  |  | x |  |  |  |  |  |  |  |  |  |  |
| Riedel-Heller SG, Busse A, Aurich C, Matschinger H, Angermeyer MC. Incidence of dementia according to DSM-III-R and ICD-10: results of the Leipzig Longitudinal Study of the Aged (LEILA75+), Part 2. Br J Psychiatry. 2001;179(3):255-260. doi:10.1192/bjp.179.3.255 |  |  |  |  |  |  |  |  |  |  |  |  |  |  |  |  | x |
| Ritchie K, Carrière I, Ritchie CW, Berr C, Artero S, Ancelin M-L. Designing prevention programmes to reduce incidence of dementia: prospective cohort study of modifiable risk factors. BMJ. 2010;341:c3885. doi:10.1136/ bmj.c3885 |  |  |  |  |  |  |  |  |  |  |  |  |  |  |  |  | x |
| Scazufca M, Menezes PR, Araya R, et al; Sao Paulo Ageing & Health Study. Risk factors across the life course and dementia in a Brazilian population: results from the Sao Paulo Ageing & Health Study (SPAH). Int J Epidemiol. 2008;37(4):879-890. doi:10.1093/ije/dyn125 |  |  |  |  |  |  |  |  |  |  |  |  |  |  |  |  | x |
| Seshadri S, Wolf PA, Beiser A, et al. Lifetime risk of dementia and Alzheimer’s disease. The impact of mortality on risk estimates in the Framingham Study. Neurology 1997; 49: 1498–504. |  |  |  |  |  |  |  |  |  |  | x |  |  |  |  |  |  |
| Sink KM, Leng X, Williamson J, Kritchevsky SB, Yaffe K, Kuller L, Yasar S, Atkinson H, Robbins M, Psaty B, Goff DC Jr. Angiotensin-converting enzyme inhibitors and cognitive decline in older adults with hypertension: results from the Cardiovascular Health Study. Arch Intern Med. 2009 Jul 13;169(13):1195-202. doi: 10.1001/archinternmed.2009.175. PMID: 19597068; PMCID: PMC2881686. |  |  |  | x |  |  |  |  |  |  |  |  | x |  |  | x |  |
| Stewart R, Xue QL, Masaki K, Petrovitch H, Ross GW, White LR, Launer LJ. Change in blood pressure and incident dementia: a 32-year prospective study. Hypertension. 2009 Aug;54(2):233-40. doi: 10.1161/HYPERTENSIONAHA.109.128744. Epub 2009 Jun 29. PMID: 19564551; PMCID: PMC3136040. (HAAS - Honolulu Heart Program/Honolulu-Asia Aging Study) |  |  |  | x | x |  |  |  |  | x |  |  |  |  |  |  |  |
| Sydney Memory and Ageing Study (MAS). Sachdev PS, Brodaty H, Reppermund S, et al; Memory and Ageing Study Team. The Sydney Memory and Ageing Study (MAS): methodology and baseline medical and neuropsychiatric characteristics of an elderly epidemiological non-demented cohort of Australians aged 70-90 years. Int Psychogeriatr. 2010;22(8):1248-1264. doi:10.1017/S1041610210001067 |  |  |  |  |  |  |  |  | x |  |  |  |  |  |  |  | x |
| T. van Middelaar, L.A. van Vught, E.P.M. van Charante, L.S. Eurelings, S.A. Ligthart, J.W. van Dalen, et al., Lower dementia risk with different classes of antihypertensive medication in older patients, J. Hypertens. 35 (10) (2017) 2095–2101. PREDIVA |  |  |  |  |  |  |  | x |  |  |  |  |  |  |  |  |  |
| Tan ECK, Qiu C, Liang Y, Wang R, Bell JS, Fastbom J, Fratiglioni L, Johnell K. Antihypertensive Medication Regimen Intensity and Incident Dementia in an Older Population. J Am Med Dir Assoc. 2018 Jul;19(7):577-583. doi: 10.1016/j.jamda.2017.11.017. Epub 2018 Jan 3. PMID: 29306604. (SNAC-K) |  |  |  | x |  |  |  |  |  |  |  |  |  |  |  |  |  |
| The 3 Cities study. |  |  |  |  |  |  |  |  | x |  |  |  |  |  |  |  |  |
| The 3 Cities study. Ref from adesuyan: Tully PJ, Dartigues JF, Debette S, et al. Dementia risk with antihypertensive use and blood pressure variability. Neurology. 2016;87(6):601-8. doi: 10.1212/ WNL.0000000000002946 | x |  |  | x | x |  |  | x |  |  |  |  |  |  |  |  |  |
| The 90+ study (california) |  |  |  |  |  |  |  |  | x |  |  |  |  |  |  |  |  |
| The Einstein Aging study (EAS). Katz MJ, Lipton RB, Hall CB, et al. Age-specific and sex-specific prevalence and incidence of mild cognitive impairment, dementia, and Alzheimer dementia in blacks and whites: a report from the Einstein Aging Study. Alzheimer Dis Assoc Disord. 2012;26(4):335-343. doi:10.1097/WAD.0b013e31823dbcfc |  |  |  |  |  |  |  |  | x |  |  |  |  |  |  |  | x |
| The Ginkgo Evaluation and Memory trial (GEM). "Yasar S, Xia J, Yao W et al. Antihypertensive drugs decrease risk of Alzheimer disease: Ginkgo Evaluation of Memory Study. Neurology 2013; 81: 896– 903." |  |  |  |  |  |  | x | x | x |  |  |  |  | x |  |  |  |
| The Gothenburg H70 Birth Cohort Studies 1930 cohort |  |  |  |  |  |  |  |  | x |  |  |  |  |  |  |  |  |
| The Gothenburg H70 Birth Cohort Studies Prospective studies of women (PPSW) 1922 cohort |  |  |  |  |  |  |  |  | x |  |  |  |  |  |  |  |  |
| Rydberg Sterner T, Ahlner F, Blennow K, et al. The Gothenburg H70 Birth cohort study 2014-16: design, methods and study population. Eur J Epidemiol. 2019;34(2):191-209. doi:10.1007/s10654-018-0459-8 |  |  |  |  |  |  |  |  |  |  |  |  |  |  |  |  | x |
| The Invecchiamento Cerebrale in Abbiategrasso study (InveCe.Ab). Guaita A, Colombo M, Vaccaro R, et al. Brain aging and dementia during the transition from late adulthood to old age: design and methodology of the “Invece.Ab” population-based study. BMC Geriatr. 2013;13(1):98. doi:10. 1186/1471-2318-13-98 |  |  |  |  |  |  |  |  | x |  |  |  |  |  |  |  | x |
| The Irish Longitudinal Study on Ageing (TILDA) |  |  |  |  |  |  |  |  | x |  |  |  |  |  |  |  |  |
| *Counts* | 4 | 7 | 8 | 13 | 7 | 5 | 8 | 11 | 28 | 8 | 7 | 5 | 12 | 5 | 4 | 10 | 17 |
|  |  | **CCA= n-r/(rxc)-r** |  |  |  | **5%** |  |  |  |  |  |  |  |  |  |  |  |
|  |  | N of study apperances (N) | N of rows r | N of reviews c |  |  |  |  |  |  |  |  |  |  |  |  |  |
|  |  | 135 | 68 | 17 |  |  |  |  |  |  |  |  |  |  |  |  |  |

Table 2: Statins

|  | Olmastroni et al., 2022 | Poly et al 2020 | Chu et al 2018 | Zhang et al 2018 | Song et al 2013 | Wong et al 2013 | Zhou et al 2007 |  |
| --- | --- | --- | --- | --- | --- | --- | --- | --- |
| Ancelin M, Carrière I, Barberger-Gateau P, et al. Lipid lowering agents, cognitive decline, and dementia: the three-city study. J Alzheimers Dis 2012;30:629–37. | x | x | x | x |  |  |  | 4 |
| Arvanitakis Z, Schneider J, Wilson R, et al. Statins, incident Alzheimer disease, change in cognitive function, and neuropathology. Neurology 2008;70(19 pt 2):1795–802. |  | x |  | x |  |  |  | 2 |
| Benito-León J, Louis E, Vega S, et al. Statins and cognitive functioning in the elderly: a population-based study. J Alzheimers Dis 2010;21:95–102. |  |  |  | x |  |  |  | 1 |
| Bettermann K, Arnold A, Williamson J, et al. Statins, risk of dementia, and cognitive function: secondary analysis of the ginkgo evaluation of memory study. J Stroke Cerebrovasc Dis 2012;21:436–44. | x | x | x | x |  | x |  | 5 |
| Beydoun M, Beason-Held L, Kitner-Triolo M, et al. Statins and serum cholesterol’s associations with incident dementia and mild cognitive impairment. J Epidemiol Community Health 2011;65:949–57. | x | x | x | x |  | x |  | 5 |
| Chao T-F, Liu C-J, Chen S-J, Wang K-L, Lin Y-J, Chang S-L, Lo L-W, Hu Y-F, Tuan T-C, Chen T-J, Lip GYH, Chiang C-E, Chen S-A. Statins and the risk of dementia in patients with atrial fibrillation: a nationwide population-based cohort study. Int J Cardiol 2015;196:91–97. | x | x |  |  |  |  |  | 2 |
| Chen PY, Liu SK, Chen CL, Wu CS. Long-term statin use and dementia risk in Taiwan. J Geriatr Psychiatry Neurol 2014;27:165–171. | x | x |  |  |  |  |  | 2 |
| Chen JM, Chang CW, Chang TH, Hsu CC, Horng JT, Sheu WH. Effects of statins on incident dementia in patients with type 2 DM: a population-based retrospective cohort study in Taiwan. PLoS One 2014;9:e88434. | x | x | x | x |  |  |  | 4 |
| Chitnis AS, Aparasu RR, Chen H, Kunik ME, Schulz PE, Johnson ML. Use of statins and risk of dementia in heart failure: a retrospective cohort study. Drugs Aging 2015;32:743–754. | x | x | x |  |  |  |  | 3 |
| Chou C, Chou Y, Chou Y, et al. Statin use and incident dementia: a nationwide cohort study of Taiwan. Int J Cardiol 2014;173:305–10. | x | x |  | x |  |  |  | 3 |
| Chuang CS, Lin CL, Lin MC, Sung FC, Kao CH. Decreased prevalence of dementia associated with statins: a national population-based study. Eur J Neurol 2015; 22:912–918. | x | x |  |  |  |  |  | 2 |
| Corrao G, Ibrahim B, Nicotra F, et al. Long-term use of statins reduces the risk of hospitalization for dementia. Atherosclerosis 2013;230: 171–6. |  |  |  | x |  |  |  | 1 |
| Cramer C, Haan M, Galea S, et al. Use of statins and incidence of dementia and cognitive impairment without dementia in a cohort study. Neurology 2008;71:344–50. | x | x | x | x | x | x |  | 6 |
| Glasser S, Wadley V, Judd S, et al. The association of statin use and statin type and cognitive performance: analysis of the reasons for geographic and racial differences in stroke (REGARDS) study. Clin Cardiol 2010;33:280–8. |  |  |  | x |  |  |  | 1 |
| Green R, McNagny S, Jayakumar P, et al. Statin use and the risk of Alzheimer’s disease: the MIRAGE study. Alzheimers Dement 2006;2: 96–103. |  | x |  | x |  |  |  | 2 |
| Haag M, Hofman A, Koudstaal P, et al. Statins are associated with a reduced risk of Alzheimer disease regardless of lipophilicity. The Rotterdam Study. J Neurol Neurosurg Psychiatry 2009;80:13–7. |  | x |  | x | x |  |  | 3 |
| Hajjar I, Schumpert J, Hirth V, et al. The impact of the use of statins on the prevalence of dementia and the progression of cognitive impairment. J Gerontol A Biol Sci Med Sci 2002;57:M414–418. |  |  |  | x |  |  |  | 1 |
| Heart Protection Study Collaborative Group. MRC/BHF heart protection study of cholesterol lowering with simvastatin in 20,536 high-risk individuals:A randomised placebo-controlled trial. Lancet 2002; 360(9326): 7–22. DOI: 10.1016/S0140–6736 (02)09327–3 |  |  |  |  |  | x |  | 1 |
| Hendrie H, Hake A, Lane K, et al. Statin use, incident dementia and Alzheimer disease in elderly African Americans. Ethn Dis 2015;25: 345–54 | x | x | x | x |  |  |  | 4 |
| Hippisley-Cox J, Coupland C. Unintended effects of statins in men and women in England and Wales: population based cohort study using the QResearch database. BMJ 2010;340:c2197 | x | x | x | x |  | x |  | 5 |
| Jick H, Zornberg G, Jick S, et al. Statins and the risk of dementia. Lancet 2000;356:1627–31. | x | x |  | x | x | x | x | 6 |
| Li G, Higdon R, Kukull W, et al. Statin therapy and risk of dementia in the elderly: a community-based prospective cohort study. Neurology 2004;63:1624–8. | x | x | x | x | x | x | x | 7 |
| Li G, Larson EB, Sonnen JA, Shofer JB, Petrie EC, Schantz A, et al. Statin therapy is associated with reduced neuropathologic changes of Alzheimer disease. Neurology. 2007 Aug; 69(9): 878–85. |  | x |  |  | x |  |  | 2 |
| Li G, Shofer JB, Rhew IC, Kukull WA, Peskind ER, McCormick W, Bowen JD, Schellenberg GD, Crane PK, Breitner JCS, Larson EB. Age-varying association between statin use and incident Alzheimer’s disease. J Am Geriatr Soc 2010;58: 1311–1317. |  | x |  | x |  |  |  | 2 |
| Lin F, Chuang Y, Hsieh H, et al. Early statin use and the progression of Alzheimer disease: a total population-based case–control study. Medicine (Baltimore) 2015;94:e2143. |  | x |  | x |  |  |  | 2 |
| Mandas A, Mereu R, Catte O, et al. Cognitive impairment and agerelated vision disorders: their possible relationship and the evaluation of the use of aspirin and statins in a 65 years-and-over Sardinian population. Front Aging Neurosci 2014;6:309 |  |  |  | x |  |  |  | 1 |
| Masse I, Bordet R, Deplanque D, et al. Lipid lowering agents are associated with a slower cognitive decline in Alzheimer’s disease. J Neurol Neurosurg Psychiatry 2005;76:1624–9. |  |  |  | x |  |  |  | 1 |
| Parikh N, Morgan R, Kunik M, et al. Risk factors for dementia in patients over 65 with diabetes. Int J Geriatr Psychiatry 2011;26:749–57. | x | x | x | x |  |  |  | 4 |
| Rea TD, Breitner JC, Psaty BM, Fitzpatrick AL, Lopez OL, Newman AB, Hazzard WR, Zandi PP, Burke GL, Lyketsos CG, Bernick C, Kuller LH. Statin use and the risk of incident dementia: the Cardiovascular Health Study. Arch Neurol 2005;62: 1047–1051. | x | x | x | x | x | x | x | 7 |
| Reitz C, Tang MX, Luchsinger J, Mayeux R. Relation of plasma lipids to Alzheimer disease and vascular dementia. Arch Neurol 2004;61:705–714. |  | x |  |  |  |  |  | 1 |
| Rockwood K, Kirkland S, Hogan DB, MacKnight C, Merry H, Verreault R, Wolfson C, McDowell I. Use of lipid-lowering agents, indication bias, and the risk of dementia in community-dwelling elderly people. Arch Neurol 2002;59:223–227. | x | x |  |  |  |  | x | 3 |
| Rodriguez EG, Dodge HH, Birzescu MA, et al: Use of lipid-lowering drugs in older adults with and without dementia: a communitybased epidemiological study. J Am Geriatr Soc 2002; 50: 1852–1856. |  |  |  |  |  |  | x | 1 |
| Smeeth L, Douglas I, Hall A, et al. Effect of statins on a wide range of health outcomes: a cohort study validated by comparison with randomized trials. Br J Clin Pharmacol 2009;67:99–109. | x | x |  | x | x | x |  | 5 |
| Solomon, A., Soininen, H., Laatikainen, T., Tuomilehto, J. & Kivipelto, M. Statins and dementia prevention: A population-based study (FINRISK). Alzheimer’s & Dementia 5, 292 (2009). |  |  | x |  |  |  |  | 1 |
| Sparks D, Kryscio R, Sabbagh M, et al. Reduced risk of incident AD with elective statin use in a clinical trial cohort. Curr Alzheimer Res 2008;5:416–21. |  | x |  | x |  |  |  | 2 |
| Szwast S, Hendrie H, Lane K, et al. Association of statin use with cognitive decline in elderly African Americans. Neurology 2007;69:1873–80. | x | x | x | x |  | x |  | 5 |
| Williams P. Lower risk of Alzheimer’s disease mortality with exercise, statin, and fruit intake. J Alzheimers Dis 2015;44:1121–9. |  |  |  | x |  |  |  | 1 |
| Wolozin B, Wang S, Li N, et al. Simvastatin is associated with a reduced incidence of dementia and Parkinson’s disease. BMC Med 2007;5:20. | x | x | x | x |  | x |  | 5 |
| Zamrini E, McGwin G, Roseman J. Association between statin use and Alzheimer’s disease. Neuroepidemiology 2004;23:94–8. |  | x |  | x |  |  |  | 2 |
| Zandi P, Sparks D, Khachaturian A, et al. Do statins reduce risk of incident dementia and Alzheimer disease? The Cache County Study. Arch Gen Psychiatry 2005;62:217–24. | x | x | x | x | x | x | x | 7 |
| Zigman, W. B. et al. Cholesterol level, statin use and Alzheimer’s disease in adults with Down syndrome. Neurosci Lett 416, 279–284, https://doi.org/10.1016/j.neulet.2007.02.023 (2007). |  |  | x |  |  |  |  | 1 |
| Zissimopoulos J, Barthold D, Brinton R, et al. Sex and race differences in the association between statin use and the incidence of Alzheimer disease. JAMA Neurol 2017;74:225–32. |  |  |  | x |  |  |  | 1 |
|  | 21 | 30 | 16 | 31 | 8 | 12 | 6 | 124 |
|  | **CCA= n-r/(rxc)-r** |  | 0.27304965 | 27% overlap aka very high according to pieper |  |  |  |  |
|  | N of study apperances (N) | N of rows r | N of reviews c |  |  |  |  |  |
|  | 124 | 47 | 7 |  |  |  |  |  |
|  |  |  |  |  |  |  |  |  |
|  |  |  |  |  |  |  |  |  |

Table 3: Antacids

|  | Virk et al. 2015 | Desai et al. 2020 | Yoon et al 2020 | Song et al. 2019 | Zhang et al 2020 | Hussain et al 2020 | Li et al. 2019 |  |
| --- | --- | --- | --- | --- | --- | --- | --- | --- |
| de Souto Barreto P, Lapeyre-Mestre M, Mathieu C, Piau C, Bouget C, Cayla F, et al. Prevalence and associations of the use of proton-pump inhibitors in nursing homes: a cross-sectional study. J Am Med Dir Assoc. 2013; 14(4):265–9. https://doi.org/10.1016/j.jamda.2012.10.018 PMID: 23211534 |  |  |  | x |  |  |  | 1 |
| Booker A, Jacob LE, Rapp M, Bohlken J, Kostev K. Risk factors for dementia diagnosis in German primary care practices. Int Psychogeriatr. 2016; 28(7):1059–65. https://doi.org/10.1017/ S1041610215002082 PMID: 26744954. |  |  |  | x |  | x |  | 2 |
| Gomm W, von Holt K, Thome F, Broich K, Maier W, Fink A, et al. Association of proton pump inhibitors with risk of dementia: A pharmacoepidemiological claims data analysis. JAMA Neurol. 2016; 73 (4):410–6. https://doi.org/10.1001/jamaneurol.2015.4791 PMID: 26882076. |  | x | x | x | x | x | x | 5 |
| Goldstein FC, Steenland K, Zhao L, Wharton W, Levey AI, Hajjar I. Proton pump inhibitors and risk of mild cognitive impairment and dementia. J Am Geriatr Soc. 2017; 65(9):1969–74. https://doi.org/10. 1111/jgs.14956 PMID: 28590010. |  | x | x | x |  | x | x | 4 |
| Gray SL, Walker RL, Dublin S, Yu O, Aiello Bowles EJ, Anderson ML, et al. Proton pump inhibitor use and dementia risk: Prospective population-based study. J Am Geriatr Soc. 2018; 66(2):247–53. https:// doi.org/10.1111/jgs.15073 PMID: 29134629. |  | x | x | x | x | x | x | 5 |
| Haenisch B, von Holt K, Wiese B, Prokein J, Lange C, Ernst A, et al. Risk of dementia in elderly patients with the use of proton pump inhibitors. Eur Arch Psychiatry Clin Neurosci. 2015; 265(5):419–28. https:// doi.org/10.1007/s00406-014-0554-0 PMID: 25341874. |  | x | x | x | x | x | x | 5 |
| Herghelegiu AM, Prada GI, Nacu R. Prolonged use of proton pomp inhibitors and cognitive function in older adults. Farmacia 2016; 64: 262–7. |  |  |  |  | x | x | x | 2 |
| Huang ST, Tseng LY, Chen LK, Peng LN, Hsiao FY. Does long-term proton pump inhibitor use increase risk of dementia? Not really! Results of the group-based trajectory analysis. Clin. Pharmacol. Ther. 2019. https://doi.org/10.1002/cpt.1430 |  |  |  |  |  | x |  | 1 |
| Hwang IC, Chang J, Park SM. A nationwide population-based cohort study of dementia risk among acid suppressant users. Am J Geriatr Psychiatry. 2018; 26(11):1175–83. https://doi.org/10.1016/j.jagp. 2018.06.002 PMID: 30072307. |  | x |  | x | x | x |  | 4 |
| Imfeld P, Bodmer M, Jick SS, Meier CR. Proton pump inhibitor use and risk of developing alzheimer’s disease or vascular dementia: A case-control analysis. Drug Saf. 2018; 41(12):1387–96. https://doi.org/ 10.1007/s40264-018-0704-9 PMID: 30146658 |  |  |  | x |  | x |  | 2 |
| Moayyedi P, Eikelboom J W, Bosch J. Safety of proton pump inhibitors based on a large, multi-year, randomized trial of patients receiving rivaroxaban or aspirin. Gastroenterology 2019; 157: 682–91. |  | x |  |  |  |  |  | 1 |
| Liao KF, Chuang HY, Lai SW. Association between proton pump inhibitor use and Alzheimer’s disease in older adults. J. Am. Geriatr. Soc. 2018; 66: 1848–50. |  |  |  |  |  | x |  | 1 |
| Räihä I, Kaprio J, Koskenvuo M, Rajala T, Sourander L. Environmental differences in twin pairs discordant for Alzheimer’s disease. J Neurol Neurosurg Psychiatry. 1998;65:785–787. | x |  |  |  |  |  |  | 1 |
| Tai SY, Chien CY, Wu DC, Lin KD, Ho BL, Chang YH, et al. Risk of dementia from proton pump inhibitor use in Asian population: A nationwide cohort study in Taiwan. PLoS One. 2017; 12(2):e0171006. https://doi.org/10.1371/journal.pone.0171006 PMID: 28199356. |  |  | x | x | x | x | x | 4 |
| Taipale H, Tolppanen AM, Tiihonen M, Tanskanen A, Tiihonen J, Hartikainen S. No association between proton pump inhibitor use and risk of alzheimer’s disease. Am J Gastroenterol. 2017; 112 (12):1802–8. https://doi.org/10.1038/ajg.2017.196 PMID: 28695906. |  |  |  | x |  | x |  | 2 |
| Tyas SL, Manfreda J, Strain LA, Montgomery PR. Risk factors for Alzheimer’s disease: a population-based, longitudinal study in Manitoba, Canada. Int J Epidemiol. 2001;30:590–597. | x |  |  |  |  |  |  | 1 |
|  | 2 | 6 | 5 | 10 | 6 | 12 | 6 |  |
|  |  | **CCA= n-r/(rxc)-r** |  | **32%** |  |  |  |  |
|  |  | N of study apperances (N) |  | N of reviews c |  |  |  |  |
|  |  | 47 |  | 7 |  |  |  |  |

Table 4: Non-steroidal anti-inflammatory drugs

|  | Zhang et al 2018 | Etminan et al 2003 | Szekely et al 2004 | Ji et al2019 | deCraen et al 2005 | Veronese et al., 2017 |  |
| --- | --- | --- | --- | --- | --- | --- | --- |
| Ancelin, M. L., Carrière, I., Helmer, C., Rouaud, O., Pasquier, F., Berr, C., et al. (2012). Steroid and nonsteroidal anti-inflammatory drugs, cognitive decline, and dementia. Neurobiol. Aging 33, 2082–2090. doi: 10.1016/j.neurobiolaging.2011.09.038 | x |  |  | x |  |  |  |
| Arvanitakis, Z., Grodstein, F., Bienias, J. L., Schneider, J. A., Wilson, R. S., Kelly, J. F., et al. (2008). Relation of NSAIDs to incident AD, change in cognitive function, and AD pathology. Neurology 70, 2219–2225. doi: 10.1212/01.wnl.0000313813.48505.86 | x |  |  |  |  |  |  |
| Beard CM, Waring SC, O’Brien PC, et al. Nonsteroidal antiinflammatory drug use and Alzheimer’s disease. A case-control study in Rochester, Minnesota, 1980 through 1984. Mayo Clin Proc 1998;73:951–5. |  | x | x |  | x |  |  |
| Breitner, J. C. S., Haneuse, S. J. P. A., Walker, R., Dublin, S., Crane, P. K., Gray, S. L., et al. (2009). Risk of dementia and AD with prior exposure to NSAIDs in an elderly community-based cohort. Neurology 72, 1899–1905. doi: 10.1212/WNL.0b013e3181a18691 | x |  |  | x |  |  |  |
| Breitner, J. C., Welsh, K. A., Helms, M. J., Gaskell, P. C., Gau, B. A., Roses, A. D., et al. (1995). Delayed onset of Alzheimer’s disease with nonsteroidal antiinflammatory and histamine H2 blocking drugs. Neurobiol. Aging 16, 523–530. doi: 10.1016/0197-4580(95)00049-K | x | x | x |  |  |  |  |
| Anti-inflammatory drugs protect against Alzheimer disease at low doses Broe, G Anthony; Grayson, David A; Creasey, Helen M; Waite, Louise M; et al.  Archives of Neurology; Chicago Vol. 57, Iss. 11, (Nov 2000): 1586-91 |  |  | x |  |  |  |  |
| Chang, C.W., Horng, J. T., Hsu, C. C., and Chen, J.M. (2016a).Mean daily dosage of aspirin and the risk of incident Alzheimer’s Dementia in patients with type 2 diabetes mellitus: a nationwide retrospective cohort study in Taiwan. J. Diabetes Res. 2016, 8. doi: 10.1155/2016/9027484 | x |  |  |  |  |  |  |
| Chang, K. H., Hsu, Y. C., Hsu, C. C., Lin, C. L., Hsu, C. Y., Lee, C. Y., et al. (2016b). Prolong exposure of NSAID in patients with RA will decrease the risk of dementia: a nationwide population-based cohort study. Medicine 95:e3056. doi: 10.1097/MD.0000000000003056 | x |  |  | x |  |  |  |
| Cornelius, C., Fastbom, J.,Winblad, B., and Viitanen,M. (2004). Aspirin, NSAIDs, risk of dementia, and influence of the apolipoprotein E epsilon 4 allele in an elderly population. Neuroepidemiology 23, 135–143. doi: 10.1159/000075957 | x |  |  | x | x |  |  |
| Côté, S., Carmichael, P. H., Verreault, R., Lindsay, J., Lefebvre, J., and Laurin, D. (2012). Nonsteroidal anti-inflammatory drug use and the risk of cognitive impairment and Alzheimer’s disease. Alzheimer’s Dement. 8, 219–226. doi: 10.1016/j.jalz.2011.03.012 | x |  |  | x |  |  |  |
| Fischer, P., Zehetmayer, S., Jungwirth, S., Weissgram, S., Krampla, W., Hinterberger, M., et al. (2008). Risk factors for Alzheimer dementia in a community-based birth cohort at the age of 75 years. Dement. Geriatr. Cogn. Disord. 25, 501–507. doi: 10.1159/000128577 | x |  |  |  |  |  |  |
| Fourrier A, Letenneur L, Bégaud B, et al. Nonsteroidal antiinflammatory drug use and cognitive function in the elderly: inconclusive results from a population-based cohort study. J Clin Epidemiol 1996;49:1201. |  | x |  | x | x |  |  |
| Henderson AS, Jorm AF, Christensen H, et al. Aspirin, antiinflammatory drugs and risk of dementia. Int J Geriat Psychiatry 1997;12:926–30. |  | x |  | x | x |  |  |
| in t’ Veld, B. A., Ruitenberg, A., Hofman, A., Launer, L. J., van Duijn, C. M., Stijnen, T., et al. (2001). Nonsteroidal antiinflammatory drugs and the risk of Alzheimer’s disease. N. Engl. J. Med. 345, 1515–1521. doi: 10.1056/NEJMoa010178 | x | x | x |  | x |  |  |
| Jonker C, Comijs HC, Smit JH. Does aspirin or other NSAIDs reduce the risk of cognitive decline in elderly persons? Results from a populationbased study. Neurobiol Aging 2003;24:583–588. |  |  |  |  |  | x |  |
| Kern S, Skoog I, € Ostling S et al. Does low-dose acetylsalicylic acid prevent cognitive decline in women with high cardiovascular risk? A 5-year followup of a non-demented population-based cohort of Swedish elderly women BMJ Open 2012;3;2(5). |  |  |  |  |  | x |  |
| Kelley BJ, McClure LA, Unverzagt FW, et al. Regular aspirin use does not reduce risk of cognitive decline. J Am Geriatr Soc. 2015;63:390–392. |  |  |  |  |  | x |  |
| Kukull WA, Larson EB, Stergachis A, et al. Non-steroidal antiinflammatory drug use and risk of Alzheimer’s disease. (Abstract). Neurology 1994;44(suppl 2):A237. |  |  |  |  | x |  |  |
| Landi, F., Cesari, M., Onder, G., Russo, A., Torre, S., and Bernabei, R. (2003). Non-steroidal anti-inflammatory drug (NSAID) use and Alzheimer disease in community-dwelling elderly patients. Am. J. Geriatr. Psychiatry 11, 179–185. doi: 10.1097/00019442-200303000-00008 | x |  | x |  |  |  |  |
| Lindsay J, Laurin D, Verreault R, et al. Risk factors for Alzheimer’s disease: a prospective analysis from the Canadian Study of Health and Aging. Am J Epidemiol 2002;156:445–53. |  | x |  |  | x |  |  |
| Nilsson, S. E., Johansson, B., Takkinen, S., Berg, S., Zarit, S., McClearn, G., et al. (2003). Does aspirin protect against Alzheimer’s dementia? a study in a Swedish population-based sample aged ≥80 years. Eur. J. Clin. Pharmacol. 59, 313–319. doi: 10.1007/s00228-003-0618-y | x |  |  |  |  | x |  |
| Stewart,W. F., Kawas, C., Corrada,M., andMetter, E. J. (1997). Risk of Alzheimer’s disease and duration of NSAID use. Neurology 48, 626–32. | x | x |  |  | x |  |  |
| Szekely, C. A., Breitner, J. C., Fitzpatrick, A. L., Rea, T. D., Psaty, B. M., Kuller, L. H., et al. (2008). NSAID use and dementia risk in the Cardiovascular | x |  |  | x |  |  |  |
| Wichmann, M. A., Cruickshanks, K. J., Carlsson, C. M., Chappell, R., Fischer, M. E., Klein, B. E. K., et al. (2016). NSAID use and incident cognitive impairment in a population-based cohort. Alzheimer Dis. Assoc. Disord. 30, 105–112. doi: 10.1097/WAD.00000000000 00098 | x |  |  |  |  |  |  |
| Williams-Gray C, Mason S, Foltinye T et al. Williams Gray.pdf 2014;24: S553. |  |  |  |  |  | x |  |
| Wolfson C, Perrault A, Moride A, et al. A case-control analysis of nonsteroidal anti-inflammatory drugs and Alzheimer’s disease: are they protective? Neuroepidemiology 2002;21:81–6. |  |  |  |  | x |  |  |
| The Canadian Study of Health and Aging: Risk factors for Alzheimer's disease in Canada Canadian Study of Health & Aging.  Neurology Vol. 44, Iss. 11, (Nov 1994): 2073-2080. |  | x | x |  |  |  |  |
| Zandi, P. P., Anthony, J. C., Hayden, K. M., Mehta, K., Mayer, L., and Breitner, J. C. S. (2002). Reduced incidence of AD with NSAID but not H2 receptor antagonists: the cache county study. Neurology 59, 880–886. doi: 10.1212/WNL.59.6.880 | x | x | x |  | x |  |  |
|  | 16 | 9 | 7 | 8 | 10 | 5 | 55 |
|  | **CCA= n-r/(rxc)-r** | **19%** |  |  |  |  |  |
|  | N of study apperances (N) | N of rows r | N of reviews c |  |  |  |  |
|  | 55 | 28 | 6 |  |  |  |  |
|  |  |  |  |  |  |  |  |

Table 5: Medications for diabetes

|  | Campbell et al, 2018 | Ye et al, 2016 | McMillan et al, 2018 | Zhou et al, 2020 | Kuate Defo et al 2024 | Tang et al 2023 | Tian et al 2023 |
| --- | --- | --- | --- | --- | --- | --- | --- |
| Akimoto H, Negishi A, Oshima S, et al. Antidiabetic drugs for the risk of Alzheimer disease in patients with type 2 DM using FAERS. Am J Alzheimers Dis Other Demen. 2020;35:1533317519899546. |  |  |  |  | x |  | x |
| Bohlken J, Jacob L, Kostev K. Association between the use of antihyperglycemic drugs and dementia risk: a case-control study. J Alzheimers Dis. 2018;66(2):725-732. |  |  |  |  |  | x | x |
| Chen KC, Chung CH, Lu CH, et al. Association between the use of dipeptidyl peptidase 4 inhibitors and the risk of dementia among patients with type 2 diabetes in Taiwan. J Clin Med. 2020;9 (3):660. |  |  |  |  | x | x | x |
| Cheng C, Lin CH, Tsai YW, et al. Type 2 diabetes and antidiabetic medications in relation to dementia diagnosis. J Gerontol A Biol Sci Med Sci 2014;69:1299–305. | x | x | x | x | x |  | x |
| Chou PS, Ho BL, Yang YH. Effects of pioglitazone on the incidence of dementia in patients with diabetes. J Diabetes Complications 2017;31:1053–7. |  |  | x | x | x |  | x |
| Fei M, Yan Ping Z, Ru Juan M, et al. Risk factors for dementia with type 2 diabetes mellitus among elderly people in China. Age Ageing 2013;42:398–400. |  |  | x |  |  |  |  |
| Ha J, Choi DW, Kim KJ, et al. Association of metformin use with Alzheimer's disease in patients with newly diagnosed type 2 diabetes: a population-based nested case-control study. Sci Rep. 2021;11:24069. |  |  |  |  | x |  | x |
| Heneka MT, Fink A, Doblhammer G. Effect of pioglitazone medication on the incidence of dementia. Ann Neurol 2015;78:284–94. | x | x | x |  |  |  |  |
| Hsu CC, Wahlqvist ML, Lee MS, et al. Incidence of dementia is increased in type 2 diabetes and reduced by the use of sulfonylureas and metformin. J Alzheimers Dis 2011;24:485–93. | x | x | x | x | x |  | x |
| Huang CC, Chung CM, Leu HB, et al. Diabetes mellitus and the risk of Alzheimer's disease: a nationwide population-based study. PLoS One 2014;9:e87095. | x | x | x | x |  |  |  |
| Imfeld P, Bodmer M, Jick SS, Meier CR: Metformin, other antidiabetic drugs, and risk of Alzheimer’s disease: a population-based case-control study. J Am Geriatr Soc 2012; 60: 916–921. |  | x |  |  |  |  |  |
| KimYG, Jeon JY, KimHJ, et al. Risk of dementia in older patientswith type 2 diabetes on dipeptidyl-peptidase IV inhibitors versus sulfonylureas: a real-world populationbased cohort study. J Clin Med. 2018:8(1). |  |  |  | x |  | x |  |
| Kim JY, Ku YS, Kim HJ, et al. Oral diabetes medication and risk of dementia in elderly patients with type 2 diabetes. Diabetes Res Clin Pract. 2019;154:116–23. |  |  |  | x |  | x |  |
| Kim Y, Kim HS, Lee JW, et al. Metformin use in elderly population with diabetes reduced the risk of dementia in a dose-dependent manner, based on the Korean NHIS-HEALS cohort. Diabetes Res Clin Pract. 2020;170:108496. |  |  |  |  | x |  |  |
| Kim WJ, Noh JH, Han K, Park CY. The Association between Second-Line Oral Antihyperglycemic Medication on Types of Dementia in Type 2 Diabetes: A Nationwide Real-World Longitudinal Study. Journal of Alzheimer's Disease. 2021;81(3):1263-1272. |  |  |  |  |  |  | x |
| Kuan YC, Huang KW, Lin CL, et al. Effects of metformin exposure on neurodegenerative diseases in elderly patients with type 2 diabetes mellitus. Prog Neuropsychopharmacol Biol Psychiatry 2017;79:77–83. |  |  | x | x | x |  | x |
| Kuo SC, Lai SW, Hung HC, et al. Association between comorbidities and dementia in diabetes mellitus patients: populationbased retrospective cohort study. J Diabetes Complications 2015;29:1071–6. |  |  | x |  |  |  |  |
| Lu CH, Yang CY, Li CY, et al. Lower risk of dementia with pioglitazone, compared with other second-line treatments, in metformin-based dual therapy: a population- based longitudinal study. Diabetologia. 2018;61(3):562–73. |  |  |  | x | x |  | x |
| Ma F, Wu T, Miao R, et al. Conversion of mild cognitive impairment to dementia among subjects with diabetes: a population-based study of incidence and risk factors with five years of follow-up. J Alzheimers Dis 2015;43:1441–9. |  |  | x |  | x |  |  |
| Mui JV, Zhou J, Lee S, et al. Sodium-glucose cotransporter 2 (SGLT2) inhibitors vs. dipeptidyl peptidase-4 (DPP4) inhibitors for new-onset dementia: a propensity score-matched populationbased study with competing risk analysis. Front Cardiovasc Med. 2021;8:747620. |  |  |  |  | x | x |  |
| Ng TP, Feng L, Yap KB, Lee TS, Tan CH, Winblad B: Long-term metformin usage and cognitive function among older adults with diabetes. J Alzheimers Dis 2014; 41: 61–68. | x | x |  |  | x |  | x |
| Nørgaard CH, Friedrich S, Hansen CT, et al. Treatment with glucagon-like peptide-1 receptor agonists and incidence of dementia: data from pooled double-blind randomized controlled trials and nationwide disease and prescription registers. Alzheimers Dement. 2022;8:e12268. |  |  |  |  | x | x |  |
| Orkaby AR, Cho K, Cormack J, et al. Metformin vs sulfonylurea use and risk of dementia in US veterans aged N/=65 years with diabetes. Neurology. 2017;89(18): 1877–85 |  |  |  | x | x |  | x |
| Ott A, Stolk RP, van Harskamp F, et al. Diabetes mellitus and the risk of dementia: the Rotterdam study. Neurology 1999;53:1937 |  |  | x |  |  |  |  |
| Parikh NM, Morgan RO, Kunik ME, et al. Risk factors for dementia in patients over 65 with diabetes. Int J Geriatr Psychiatry 2011;26:749–57. |  |  | x | x | x |  | x |
| Samaras K, Makkar S, Crawford JD, et al. Metformin use is associated with slowed cognitive decline and reduced incident dementia in older adults with type 2 diabetes: the Sydney memory and ageing study. Diabetes Care. 2020;43:2691-2701. |  |  |  |  | x |  | x |
| Sanke H, Mita T, Yoshii H, et al. Olfactory dysfunction predicts the development of dementia in older patients with type 2 diabetes. Diabetes Research and Clinical Practice. 2021;174. |  |  |  |  |  |  | x |
| Scherrer JF,Morley JE, Salas J, et al. Association betweenmetformin initiation and incident dementia among African American and white veterans health administration patients. Ann Fam Med. 2019;17(4):352–62. |  |  |  | x |  |  |  |
| Scherrer JF, Salas J, Floyd JS, et al. Metformin and sulfonylurea use and risk of incident dementia. Mayo Clin Proc. 2019;94(8):1444–56. |  |  |  | x | x |  |  |
| Shi Q, Liu S, Fonseca VA, et al. Effect of metformin on neurodegenerative disease among elderly adult US veterans with type 2 diabetes mellitus. BMJ Open. 2019;9 (7):e024954. |  |  |  | x | x |  | x |
| Siao WZ, Lin TK, Huang JY, Tsai CF, Jong GP. The association between sodium-glucose cotransporter 2 inhibitors and incident dementia: a nationwide population-based longitudinal cohort study. Diab Vasc Dis Res. 2022;19:14791641221098168. |  |  |  |  | x |  | x |
| Sluggett JK, KoponenM, Bell JS, et al. Metformin and risk of Alzheimer's disease among community-dwelling people with diabetes: a National Case-Control Study. J Clin Endocrinol Metab. 2020;105:e963-e972. |  |  |  |  | x |  | x |
| Tang X, Brinton RD, Chen Z, et al. Use of oral diabetes medications and the risk of incident dementia in US veterans aged ≥60 years with type 2 diabetes. BMJ Open Diabetes Res Care. 2022;10(5): e002894. |  |  |  |  | x |  |  |
| Torrandell-Haro G, Branigan GL, Brinton RD, Rodgers KE. Association between specific type 2 diabetes therapies and risk of Alzheimer's disease and related dementias in propensity-score matched type 2 diabetic patients. Front Aging Neurosci. 2022;14:878304. |  |  |  |  | x |  |  |
| Tseng 2017 - can't find ref |  |  |  | x |  |  |  |
| Tseng CH. Pioglitazone reduces dementia risk in patients with type 2 diabetes mellitus: a retrospective cohort analysis. J Clin Med. 2018;7(10). |  |  |  | x | x |  | x |
| Tseng CH. Rosiglitazone has a neutral effect on the risk of dementia in type 2 diabetes patients. Aging (Albany NY). 2019;11(9):2724–34. |  |  |  | x | x |  | x |
| Tseng C-H. Metformin and the risk of dementia in type 2 diabetes patients. Aging Dis. 2019;10:37-48. |  |  |  |  | x |  | x |
| Tseng CH. Dementia risk in type 2 diabetes patients: acarbose use and its joint effects with metformin and pioglitazone. Aging Dis. 2020;11(3):658‐667. https://doi.org/10.14336/ad.2019.0621 |  |  |  |  |  |  | x |
| Tseng CH. Vildagliptin has a neutral association with dementia risk in type 2 diabetes patients. Front Endocrinol. 2021;12:637392. |  |  |  |  | x | x |  |
| Wang CP, Lorenzo C, Habib SL, Jo B, Espinoza SE (2017) Differential effects of metformin on age related comorbidities in older men with type 2 diabetes. J Diabetes Complications 31, 679-686. | x |  |  |  |  |  |  |
| Weinstein G, Davis-Plourde KL, Conner S, et al. Association of metformin, sulfonylurea and insulin use with brain structure and function and risk of dementia and Alzheimer’s disease: pooled analysis from 5 cohorts. PLoS One. 2019;14(2): e0212293. |  |  |  | x |  |  |  |
| Wium-Andersen IK, Osler M, Jørgensen MB, Rungby J, Wium- Andersen MK. Antidiabetic medication and risk of dementia in patients with type 2 diabetes: a nested case-control study. Eur J Endocrinol. 2019;181:499-507. |  |  |  |  | x | x | x |
| Zhou B, Zissimopoulos J, Nadeem H, Crane MA, Goldman D, Romley JA. Association between exenatide use and incidence of Alzheimer's disease. Alzheimer Dement Translation Res Clinic Intervent. 2021;7(1):e12139. |  |  |  |  |  | x |  |
|  | 6 | 6 | 11 | 17 | 27 | 9 | 23 |
|  | **CCA= n-r/(rxc)-r** |  |  |  | **20%** |  |  |
|  | N of study apperances (N) | N of rows r | N of reviews c |  |  |  |  |
|  | 40 | 25 | 4 |  |  |  |  |

Table 6: Anticoagulants

|  | Mongkhon et al 2019 | Moffitt et al, 2016 |
| --- | --- | --- |
| Barber, M., Tait, R.C., Scott, J., Rumley, A., Lowe, G.D., Stott, D.J., 2004. Dementia in subjects with atrial fibrillation: hemostatic function and the role of anticoagulation. J. Thromb. Haemost. 2 (11), 1873–1878. https://doi.org/10.1111/j.1538-7836. 2004.00993.x. | x | x |
| Douiri, A., McKevitt, C., Emmett, E.S., Rudd, A.G., Wolfe, C.D., 2013. Long-term effects of secondary prevention on cognitive function in stroke patients. Circulation 128 (12), 1341–1348. https://doi.org/10.1161/CIRCULATIONAHA.113.002236. | x |  |
| Friberg, L., Rosenqvist, M., 2018. Less dementia with oral anticoagulation in atrial fibrillation. Eur. Heart J. 39 (6), 453–460. https://doi.org/10.1093/eurheartj/ehx579 | x |  |
| Liao M, Lin L, Lin J. Did warfarin and antiplatelet reduce the incidence of dementia in patients with atrial fibrillation: a nationwide cohort study. Europace 2013; 15: 923. |  | x |
| Madhavan, M., Hu, T.Y., Gersh, B.J., Roger, V.L., Killian, J., Weston, S.A., Graff-Radford, J., Asirvatham, S.J., Chamberlain, A.M., 2018. Efficacy of warfarin anticoagulation and incident dementia in a community-based cohort of atrial fibrillation. Mayo Clin. Proc. 93 (2), 145–154. https://doi.org/10.1016/j.mayocp.2017.09.021. | x |  |
| Mavaddat, N., Roalfe, A., Fletcher, K., Lip, G.Y., Hobbs, F.D., Fitzmaurice, D., Mant, J., 2014. Warfarin versus aspirin for prevention of cognitive decline in atrial fibrillation: randomized controlled trial (Birmingham Atrial Fibrillation Treatment of the Aged Study). Stroke 45 (5), 1381–1386. https://doi.org/10.1161/STROKEAHA.113. 004009. | x | x |
| Meranus D, Kukull W. Antithrombotic medication use and dementia incidence among people with mild cognitive impairment and atrial fibrillation. Alzheimers Dement 2013; 9: 612. |  | x |
|  | 5 | 4 |
|  | **CCA= n-r/(rxc)-r** | **29%** |
| N of study appearances (N) | N of rows r | N of reviews c |
| 9 | 7 | 2 |

Table 7: Hormone replacement therapy

|  | O'Brien et al 2014 | Wu et al 2020 | LeBlanc et al 2001 | Yaffe et al 1998 | Nerattini et al 2023 |
| --- | --- | --- | --- | --- | --- |
| Amaducci, L. A., Fratiglioni, L., Rocca, W. A., Fieschi, C., Livrea, P., Pedone, D., et al. (1986). Risk factors for clinically diagnosed Alzheimer's disease: a case-control study of an Italian population. Neurology 36, 922–931. doi: 10.1212/WNL.36.7.922 |  |  | x | x | x |
| Baldereschi, M., Di Carlo, A., Lepore, V., Bracco, L., Maggi, S., Grigoletto, F., et al. (1998). Estrogen-replacement therapy and Alzheimer's disease in the Italian longitudinal study on aging. Neurology 50, 996–1002. doi: 10.1212/WNL.50.4.996 |  |  |  |  | x |
| Barnes, L. L., Wilson, R. S., Schneider, J. A., Bienias, J. L., Evans, D. A., and Bennett, D. A. (2003). Gender, cognitive decline, and risk of AD in older persons. Neurology 60, 1777–1781. doi: 10.1212/01.WNL.0000065892.67099.2A |  |  |  |  | x |
| Bove, R., Secor, E., Chibnik, L. B., Barnes, L. L., Schneider, J. A., Bennett, D. A., et al. (2014). Age at surgical menopause influences cognitive decline and Alzheimer pathology in older women. Neurology 82, 222–229. doi: 10.1212/WNL.0000000000000033 |  |  |  |  | x |
| Brenner | x |  | x | x |  |
| Broe, G. A., Henderson, A. S., Creasey, H., Mccusker, E., Korten, A. E., Jorm, A. F., et al. (1990). A case-control study of Alzheimer's disease in Australia. Neurology 40, 1698–1707. doi: 10.1212/WNL.40.11.1698 |  |  | x | x | x |
| Colucci, M., Cammarata, S., Assini, A., Croce, R., Clerici, F., Novello, C., et al. (2006). The number of pregnancies is a risk factor for Alzheimer's disease. Eur. J. Neurol. 13, 1374–1377. doi: 10.1111/j.1468-1331.2006.01520.x |  |  |  |  | x |
| D.B. Petitti, V.C. Crooks, V. Chiu, J.G. Buckwalter, H.C. Chui, Incidence of dementia in long-term hormone users, Am. J. Epidemiol. 167 (6) (2008) 692–700. |  | x |  |  |  |
| Graves, A. B., White, E., Koepsell, T. D., Reifler, B. V., Van Belle, G., Larson, E. B., et al. (1990). A case-control study of Alzheimer's disease. Ann. Neurol. 28, 766–774. doi: 10.1002/ana.410280607 |  |  | x | x | x |
| Harwood, D. G., Barker, W. W., Loewenstein, D. A., Ownby, R. L., St. George-Hyslop, P., Mullan, M., et al. (1999). A cross-ethnic analysis of risk factors for AD in white Hispanics and white non-Hispanics. Neurology 52:551. doi: 10.1212/WNL.52.3.551 |  |  | x |  | x |
| Henderson, V. W., Ma, E., Pa, H., Sr, R., Stefanick, M. L., Wactawski-Wende, J., et al. (2007). Prior use of hormone therapy and incident Alzheimer’s disease in the Women’s health Initiative memory study. Neurology 68:A205 |  |  | x | x | x |
| Heyman, A., Wilkinson, W. E., Stafford, J. A., Helms, M. J., Sigmon, A. H., and Weinberg, T. (1984). Alzheimer's disease: a study of epidemiological aspects. Ann. Neurol. 15, 335–341. doi: 10.1002/ana.410150406 |  |  | x | x | x |
| Imtiaz, B., Taipale, H., Tanskanen, A., Tiihonen, M., Kivipelto, M., Heikkinen, A. M., et al. (2017a). Risk of Alzheimer's disease among users of postmenopausal hormone therapy: a nationwide case-control study. Maturitas 98, 7–13. doi: 10.1016/j.maturitas.2017.01.002 |  |  |  |  | x |
| Imtiaz, B., Tuppurainen, M., Rikkonen, T., Kivipelto, M., Soininen, H., Kröger, H., et al. (2017b). Postmenopausal hormone therapy and Alzheimer disease: a prospective cohort study. Neurology 88, 1062–1068. doi: 10.1212/WNL.0000000000003696 |  |  |  |  | x |
| J. Ryan, I. Carriere, J. Scali, J.F. Dartigues, C. Tzourio, M. Poncet, et al., Characteristics of hormone therapy, cognitive function, and dementia: the prospective 3C Study, Neurology 73 (21) (2009) 1729–1737. | x | x |  |  |  |
| J.E. Manson, R.T. Chlebowski, M.L. Stefanick, A.K. Aragaki, J.E. Rossouw, R.L. Prentice, et al., Menopausal hormone therapy and health outcomes during the intervention and extended poststopping phases of the Women’s Health Initiative randomized trials, JAMA 310 (13) (2013) 1353–1368. |  | x |  |  |  |
| Kawas | x |  | x | x |  |
| Kim, Y. J., Soto, M., Branigan, G. L., Rodgers, K., and Brinton, R. D. (2021). Association between menopausal hormone therapy and risk of neurodegenerative diseases: implications for precision hormone therapy. Alzheimers Dement (N Y) 7:e12174. doi: 10.1002/trc2.12174 |  |  |  |  | x |
| Lau, D. T., Mercaldo, N. D., Harris, A. T., Trittschuh, E., Shega, J., and Weintraub, S. (2010). Polypharmacy and potentially inappropriate medication use among community-dwelling elders with dementia. Alzheimer Dis. Assoc. Disord. 24, 56–63. doi: 10.1097/WAD.0b013e31819d6ec9 |  |  |  |  | x |
| Lindsay J, Laurin D, Verreault R, et al. Risk factors for Alzheimer’s disease: a prospective analysis from the Canadian Study of Health and Aging. Am J Epidemiol. 2002;156(5): 445–453. | x |  |  |  | x |
| Lerner, A., Cole, R., and Debanne, S. (1995). Immunological and endocrine conditions in an. Alzheimer's disease case-control study. Neuroepidemiology 14:307. |  |  |  |  | x |
| Levine, A. J., and Hewett, L. (2003). Estrogen replacement therapy and frontotemporal dementia. Maturitas 45, 83–88. doi: 10.1016/S0378-5122(03)00142-7 |  |  |  |  | x |
| Løkkegaard, L. E., Thinggaard, M., Nygaard, M., Hallas, J., Osler, M., and Christensen, K. (2022). Systemic hormone therapy and dementia: a nested case-control and co-twin control study. Maturitas 165, 113–119. doi: 10.1016/j.maturitas.2022.04.007 |  |  |  |  | x |
| M.A. Espeland, R.D. Brinton, C. Hugenschmidt, J.E. Manson, S. Craft, K. Yaffe, et al., Impact of type 2 diabetes and postmenopausal hormone therapy on incidence of cognitive impairment in older women, Diabetes Care 38 (12) (2015) 2316–2324 |  | x |  |  |  |
| Mortel, K. F., and Meyer, J. S. (1995). Lack of postmenopausal estrogen replacement therapy and the risk of dementia. J. Neuropsychiatry Clin. Neurosci. 7, 334–337. doi: 10.1176/jnp.7.3.334 |  |  | x | x | x |
| Paganini-Hill, A., and Henderson, V. W. (1994). Estrogen deficiency and risk of Alzheimer's disease in women. Am. J. Epidemiol. 140, 256–261. doi: 10.1093/oxfordjournals.aje.a117244 |  |  | x | x | x |
| Paganini-Hill, A., Corrada, M. M., and Kawas, C. H. (2020). Prior endogenous and exogenous estrogen and incident dementia in the 10th decade of life: the 90+ study. Climacteric 23, 311–315. doi: 10.1080/13697137.2020.1727876 |  |  |  |  | x |
| R.A. Whitmer, C.P. Quesenberry, J. Zhou, K. Yaffe, Timing of hormone therapy and dementia: the critical window theory revisited, Ann. Neurol. 69 (1) (2011) 163–169. |  | x |  |  |  |
| Rippon, G. A., Tang, M. X., Lee, J. H., Lantigua, R., Medrano, M., and Mayeux, R. (2006). Familial Alzheimer disease in Latinos: interaction between APOE, stroke, and estrogen replacement. Neurology 66, 35–40. doi: 10.1212/01.wnl.0000191300.38571.3e |  |  |  |  | x |
| Roberts RO, Cha RH, Knopman DS, et al. Postmenopausal estrogen therapy and Alzheimer disease: overall negative findings. Alzheimer Dis Assoc Disord. 2006;20(3):141–146. | x |  |  |  |  |
| Ryan, J., Carrière, I., Scali, J., Ritchie, K., and Ancelin, M. L. (2009b). Life-time estrogen exposure and cognitive functioning in later life. Psychoneuroendocrinology 34, 287–298. doi: 10.1016/j.psyneuen.2008.09.008 |  |  |  |  | x |
| Seshadri S, Zornberg GL, Derby LE, et al. Postmenopausal estrogen replacement therapy and the risk of Alzheimer disease. Arch Neurol. 2001;58(3):435–440. | x |  |  |  | x |
| Shao H, Breitner JC, Whitmer RA, et al. Hormone therapy and Alzheimer disease dementia: new findings from the Cache County Study. Neurology. 2012;79(18):1846–1852. | x |  |  |  | x |
| Shumaker SA, Legault C, Kuller L, et al. Conjugated equine estrogens and incidence of probable dementia and mild cognitive impairment in postmenopausal women:Women’s Health Initiative Memory Study. JAMA. 2004;291(24): 2947–2958. | x |  |  |  |  |
| Song, X., Wu, J., Zhou, Y., Feng, L., Yuan, J. M., Pan, A., et al. (2020). Reproductive and hormonal factors and risk of cognitive impairment among Singapore Chinese women. Am. J. Obstet. Gynecol. 223:e1-410.e23. doi: 10.1016/j.ajog.2020.02.032 |  |  |  |  | x |
| Tang | x |  | x | x |  |
| Waring, S. C., Rocca, W. A., Petersen, R. C., O’brien, P. C., Tangalos, E. G., and Kokmen, E. (1999). Postmenopausal estrogen replacement therapy and risk of AD: a population-based study. Neurology 52, 965–970. doi: 10.1212/WNL.52.5.965 | x |  | x |  | x |
| Whitmer, R. A., Quesenberry, C. P., Zhou, J., and Yaffe, K. (2011). Timing of hormone therapy and dementia: the critical window theory revisited. Ann. Neurol. 69, 163–169. doi: 10.1002/ana.22239 |  |  |  |  | x |
| Yoo, J., Shin, D., Han, K., Kim, D., Won, H. S., Lee, J., et al. (2020). Female reproductive factors and the risk of dementia: a nationwide cohort study. Eur. J. Neurol. 27, 1448–1458. doi: 10.1111/ene.14315 |  |  |  |  | x |
| Zandi, P. P., Carlson, M. C., Plassman, B. L., Welsh-Bohmer, K. A., Mayer, L. S., Steffens, D. C., et al. (2002). Hormone replacement therapy and incidence of Alzheimer disease in older women: the Cache County study. JAMA 288, 2123–2129. doi: 10.1001/jama.288.17.2123 |  |  |  |  | x |
| Zucchella, C., Sinforiani, E., Citterio, A., Giarracca, V., Bono, G., and Mauri, M. (2012). Reproductive life events and Alzheimer's disease in Italian women: a retrospective study. Neuropsychiatr. Dis. Treat. 8, 555–560. doi: 10.2147/NDT.S36622 |  |  |  |  | x |
|  | 10 | 5 | 12 | 10 | 31 |
|  | **CCA= n-r/(rxc)-r** | **16%** |  |  |  |
|  | N of study apperances (N) | N of rows r | N of reviews c |  |  |
|  | 37 | 21 | 4 |  |  |

Table 8: Vitamins

|  | Wang et al 2021 | Zhao et al 2022 | Zhou et al 2023 |
| --- | --- | --- | --- |
| Basambombo, L. L., Carmichael, P. H., Côt,é, S., and Laurin, D. (2017). Use of vitamin E, and C supplements for the prevention of cognitive decline. Ann. Pharmacother. 51, 118–124. doi: 10.1177/1060028016673072 | x | x | x |
| Gray, S. L., Anderson, M. L., Crane, P. K., Breitner, J. C., McCormick, W., Bowen, J. D., et al. (2008). Antioxidant vitamin supplement use and risk of dementia or Alzheimer’s disease in older adults. J. Am. Geriatr. Soc. 56, 291–295. doi: 10.1111/j.1532-5415.2007.01531.x | x | x | x |
| Kryscio, R. J., Abner, E. L., Caban-Holt, A., Lovell, M., Goodman, P., Darke, A. K., et al. (2017). Association of antioxidant supplement use and dementia in the prevention of Alzheimer’s disease by vitamin, E, and selenium trial (PREADViSE). JAMA Neurol. 74, 567–573. doi: 10.1001/jamaneurol.2016.5778 |  | x | x |
| luchsinger | x |  |  |
| Masaki, K. H. , White, L. R. , Foley, D. J. , Petrovitch, H. , Izmirlian, G. , Havlik, R. , Ross, G. & Losonczy, K. G. (2000). Association of vitamin E and C supplement use with cognitive function and dementia in elderly men. Neurology, 55 (6), 901-902. doi: 10.1212/WNL.55.6.901-a. |  | x | x |
| morris 1998 | x |  |  |
| morris 2002 | x |  |  |
| Paganini-Hill, A., Kawas, C. H., and Corrada, M.M. (2016). Lifestyle factors and dementia in the oldest-old: the 90+ study. Alzheimer Dis. Assoc. Disord. 30, 21–26. doi: 10.1097/WAD.0000000000000087 |  | x | x |
| Zandi, P. P., Anthony, J. C., Khachaturian, A. S., Stone, S. V., Gustafson, D., Tschanz, J. T., et al. (2004). Reduced risk of Alzheimer disease in users of antioxidant vitamin supplements: the Cache County Study. Arch. Neurol. 61, 82–88. doi: 10.1001/archneur.61.1.82 |  | x |  |
|  | 5 | 6 | 5 |
|  | **CCA= n-r/(rxc)-r** | **39%** |  |
|  | N of study apperances (N) | N of rows r | N of reviews c |
|  | 16 | 9 | 3 |

Table 9: Anticholinergics

|  | Dmochowski 2021 | [Pieper et al 2020](https://pubmed.ncbi.nlm.nih.gov/?term=Pieper+NT&cauthor_id=32603415) | Zheng et al 2021 |  |  |
| --- | --- | --- | --- | --- | --- |
| Ancelin ML, Artero S, Portet F, Dupuy AM, Touchon J, Ritchie K. Non‐degenerative mild cognitive impairment in elderly people and use of anticholinergic drugs: longitudinal cohort study. BMJ. 2006;332:455–459. | x |  | x |  |  |
| Bali V, Chatterjee S, Carnahan RM, Chen H, Johnson ML, Aparasu RR. Risk of dementia among elderly nursing home patients using paroxetine and other selective serotonin reuptake inhibitors. Psychiatr Serv 2015; 66: 1333–40. |  | x |  |  |  |
| Campbell NL, Boustani MA, Lane KA et al. Use of anticholinergics and the risk of cognitive impairment in an African American population. Neurology 2010; 75: 152–9. |  | x | x |  |  |
| Carriere, I., Fourrier-Reglat, A., Dartigues, J.F., Rouaud, O., Pasquier, F., Ritchie, K., Ancelin, M.L., 2009. Drugs with anticholinergic properties, cognitive decline, and dementia in an elderly general population: the 3-city study. Arch. Intern. Med. 169, 1317–1324. |  |  | x |  |  |
| Cai, X., Campbell, N., Khan, B., Callahan, C., Boustani, M., 2013. Long-term anticholinergic use and the aging brain. Alzheimers Dement. 9, 377–385. |  |  | x |  |  |
| Chatterjee S, Bali V, Carnahan RM, Johnson ML, Chen H, Aparasu RR. Anticholinergic medication use and risk of dementia among elderly nursing home residents with depression. Am J Geriatr Psychiatry 2016; 24: 485–95. |  | x | x |  |  |
| Coupland CAC, Hill T, Dening T, Morriss R, Moore M, Hippisley‐ Cox J. Anticholinergic drug exposure and the risk of dementia: a nested case‐control study. JAMA Intern Med. 2019;179:1084–1093. | x |  | x |  |  |
| Gray SL, Anderson ML, Dublin S, et al. Cumulative use of strong anticholinergics and incident dementia: a prospective cohort study. JAMA Intern Med. 2015;175:401–407. | x | x | x |  |  |
| Grossi CM, Richardson K, Fox C et al. Anticholinergic and benzodiazepine medication use and risk of incident dementia: a UK cohort study. BMC Geriatr 2019; 19: 276. in press. |  | x |  |  |  |
| Jessen, F., Kaduszkiewicz, H., Daerr, M., Bickel, H., Pentzek, M., Riedel-Heller, S., Wagner, M., Weyerer, S., Wiese, B., van den Bussche, H., Broich, K., Maier, W., 2010. Anticholinergic drug use and risk for dementia: target for dementia prevention. Eur. Arch. Psychiatry Clin. Neurosci. 260 (Suppl 2), S111–115. |  |  | x |  |  |
| Hafdi, M., Hoevenaar-Blom, M.P., Beishuizen, C.R.L., Moll van Charante, E.P., Richard, E., van Gool, W.A., 2020. Association of benzodiazepine and anticholinergic drug usage with incident dementia: a prospective cohort study of community-dwelling older adults. J. Am. Med. Dir. Assoc. 21, 188–193 e183. |  |  | x |  |  |
| Hong CT, Chan L, Wu D, Chen WT, Chien LN. Antiparkinsonism anticholinergics increase dementia risk in patients with Parkinson's disease. Parkinsonism Relat Disord. 2019;65:224–229. | x |  |  |  |  |
| Park HY, Park JW, Song HJ, Sohn HS, Kwon JW. The association between polypharmacy and dementia: a nested casecontrol study based on a 12‐year longitudinal cohort database in South Korea. PLOS One. 2017;12:e0169463. | x |  |  |  |  |
| Richardson K, Fox C, Maidment I, et al. Anticholinergic drugs and risk of dementia: case‐control study. BMJ (Clin Res Ed). 2018;361:k1315. | x | x | x |  |  |
| Naharci, M.I., Cintosun, U., Ozturk, A., Oztin, H., Turker, T., Bozoglu, E., Doruk, H., 2017. Effect of anticholinergic burden on the development of dementia in older adults with subjective cognitive decline. Psychiat. Clin. Psych. 27, 263–270. |  |  | x |  |  |
| Liu, Y.P., Chien, W.C., Chung, C.H., Chang, H.A., Kao, Y.C., Tzeng, N.S., 2020. Are anticholinergic medications associated with increased risk of dementia and behavioral and psychological symptoms of dementia? a nationwide 15-year followup cohort study in Taiwan. Front. Pharmacol. 11, 30. |  |  | x |  |  |
| Shah, R.C., Janos, A.L., Kline, J.E., Yu, L., Leurgans, S.E., Wilson, R.S., Wei, P., Bennett, D.A., Heilman, K.M., Tsao, J.W., 2013. Cognitive decline in older persons initiating anticholinergic medications. PLoS One 8, e64111. |  |  | x |  |  |
| Yang Y-W, LiuH-H, Lin T-H, ChuangH-Y,Hsieh T. Association between different anticholinergic drugs and subsequent dementia risk in patients with diabetes mellitus. PLoS One 2017; 12: e0175335. |  | x |  |  |  |
| Whalley, L.J., Sharma, S., Fox, H.C., Murray, A.D., Staff, R.T., Duthie, A.C., Deary, I.J., Starr, J.M., 2012. Anticholinergic drugs in late life: adverse effects on cognition but not on progress to dementia. J. Alzheimers Dis. 30, 253–261. |  |  | x |  |  |
|  | 6 | 7 | 14 | 27 |  |
|  |  |  |  |  |  |
|  |  |  |  |  |  |
|  | **CCA= n-r/(rxc)-r** |  |  |  | 21% |
|  | N of study apperances (N) | N of rows r | N of reviews c |  |  |
|  | 27 | 19 | 3 |  |  |

Table 10: Benzodiazepines

|  | AlDawsari et al 2022 | Lucchetta et al., 2018 | Islam et al., 2016 |  |  |
| --- | --- | --- | --- | --- | --- |
| Bietry FA, Pfeil AM, Reich O, Schwenkglenks M, Meier CR. Benzodiazepine use and risk of developing Alzheimer’s disease: a case-control study based on Swiss claims data. CNS Drugs 2017;31(3):245–51. | x | x |  |  |  |
| Billioti de Gage S, Moride Y, Ducruet T, et al. Benzodiazepine use and risk of Alzheimer’s disease: case-control study. BMJ 2014;349:g5205. | x | x | x |  |  |
| Billioti de Gage S, Begaud B, Bazin F, et al. Benzodiazepine use and risk of dementia: prospective population based study. BMJ 2012;345:e6231. |  | x | x |  |  |
| Chan T, Leung WC, Li V, et al. Association between high cumulative dose of benzodiazepine in Chinese patients and risk of dementia: a preliminary retrospective case-control study. Psychogeriatrics 2017;17(5):310–6. | x | x |  |  |  |
| Gallacher J, Elwood P, Pickering J, Bayer A, Fish M, Ben- Shlomo Y. Benzodiazepine use and risk of dementia: evidence from the Caerphilly Prospective Study (CaPS). J Epidemiol Community Health 2012;66(10):869–73. | x | x |  |  |  |
| Gerhard T, Devanand DP, Huang C, Crystal S, Olfson M. Lithium treatment and risk for dementia in adults with bipolar disorder: population-based cohort study. Br J Psychiatry. 2015;207(1):46-51. | x |  |  |  |  |
| Gray SL, Dublin S, Yu O, et al. Benzodiazepine use and risk of incident dementia or cognitive decline: prospective population based study. BMJ 2016;352:i90. | x | x |  |  |  |
| Grossi CM, Richardson K, Fox C, et al. Anticholinergic and benzodiazepine medication use and risk of incident dementia: a UK cohort study. BMC Geriatr. 2019;19(1):1-10. https://doi.org/10.1186/ s12877-019-1280-2 | x |  |  |  |  |
| Gomm W, von Holt K, Thom e F, et al. Regular benzodiazepine and Z-substance use and risk of dementia: an analysis of German claims data. J Alzheimers Dis 2016;54(2):801–8. |  | x |  |  |  |
| Hafdi M, Hoevenaar-Blom MP, Beishuizen CRL, van Charante EP, Richard E, van Gool WA. Association of benzodiazepine and anticholinergic drug usage with incident dementia: a prospective cohort study of community-dwelling older adults. J Am Med Dir Assoc. 2020; 21(2):188-193. https://doi.org/10.1016/j.jamda.2019.05.010 | x |  |  |  |  |
| Imfeld P, Bodmer M, Jick SS, Meier CR. Benzodiazepine use and risk of developing Alzheimer’s disease or vascular dementia: a case-control analysis. Drug Saf 2015;38(10):909–19. | x | x | x |  |  |
| Mawanda F, Wallace RB, McCoy K, Abrams TE. PTSD, psychotropic medication use, and the risk of dementia among US veterans: a retrospective cohort study. J Am Geriatr Soc 2017;65(5):1043–50. |  | x |  |  |  |
| Nafti M, Sirois C, Kröger E, Carmichael PH, Laurin D. Is benzodiazepine use associated with the risk of dementia and cognitive impairment–not dementia in older persons? The Canadian Study of Health and Aging. Ann Pharmacother. 2020;54(3):219-225. https:// doi.org/10.1177/1060028019882037 | x |  |  |  |  |
| Lagnaoui R, Bégaud B, Moore N, et al. Benzodiazepine use and risk of dementia: a nested case–control study. J Clin Epidemiol. 2002;55(3): 314-318. | x |  | x |  |  |
| Lee J, Jung SJ, Choi JW, Shin A, Lee YJ. Use of sedative-hypnotics and the risk of Alzheimer's dementia: a retrospective cohort study. PLoS ONE. 2018;13(9):1-13. https://doi.org/10.1371/journal.pone. 0204413 | x |  |  |  |  |
| Park HY, Park JW, Song HJ, Sohn HS, Kwon JW. The association between polypharmacy and dementia: a nested case–control study based on a 12-year longitudinal cohort database in South Korea. PLoS ONE. 2017;12(1):1-17. https://doi.org/10.1371/journal.pone. 0169463 | x |  |  |  |  |
| Richardson K, Mattishent K, Loke YK, et al. History of benzodiazepine prescriptions and risk of dementia: possible bias due to prevalent | x |  |  |  |  |
| Shash D, Kurth T, Bertrand M, et al. Benzodiazepine, psychotropic medication, and dementia: a population-based cohort study. Alzheimer’s Dement 2016;12(5):604–13. | x | x | x |  |  |
| Shih HI, Lin CC, Tu YF, et al: An increased risk of reversible dementia may occur after zolpidem derivative use in the elderly population: a population-based case-control study. Medicine (Baltimore) 2015; 94:e809. |  |  | x |  |  |
| Takada M, Fujimoto M, Hosomi K. Association between benzodiazepine use and dementia: data mining of different medical databases. Int J Med Sci. 2016;13(11):825-834. https://doi.org/10.7150/ijms. 16185 | x |  |  |  |  |
| Tapiainen V, Taipale H, Tanskanen A, Tiihonen J, Hartikainen S, Tolppanen AM. The risk of Alzheimer's disease associated with benzodiazepines and related drugs: a nested case–control study. Acta Psychiatr Scand. 2018;138(2):91-100. https://doi.org/10.1111/acps. 12909 | x |  |  |  |  |
| Tolppanen A-M, Taipale H, Hartikainen S. Head or brain injuries and Alzheimer’s disease: a nested case-control register study. Alzheimer’s Dement 2017;13(12):1371–9. | x | x |  |  |  |
| Tseng LY, Huang ST, Peng LN, Chen LK, Hsiao FY. Benzodiazepines, z-hypnotics, and risk of dementia: special considerations of half-lives and concomitant use. Neurotherapeutics. 2020;17(1):156-164. https://doi.org/10.1007/2Fs13311-019-00801-9 | x |  |  |  |  |
| Wu CS, Wang SC, Chang IS, Lin KM: The association between dementia and long-term use of benzodiazepine in the elderly: nested case-control study using claims data. Am J Geriatr Psychiatry 2009; 17: 614–620. |  |  | x |  |  |
| Wu CS, Ting TT, Wang SC, Chang IS, Lin KM. Effect of benzodiazepine discontinuation on dementia risk. Am J Geriatr Psychiatry 2011;19(2):151–9. | x | x | x |  |  |
|  | 20 | 12 | 8 | 40 |  |
|  |  |  |  |  |  |
|  |  |  |  |  |  |
|  | **CCA= n-r/(rxc)-r** |  |  |  |  |
|  | N of study apperances (N) | N of rows r | N of reviews c |  | 30% |
|  | 40 | 25 | 3 |  |  |

Table 11: antidepressants

|  | AlDawsari et al 2022 | Wang et al 2023 |
| --- | --- | --- |
| Grossi 2019 | x |  |
| Lee 2018 | x |  |
| Brodrick 2016 | x |  |
| Liu 2020 | x |  |
| Richardson 2018 | x |  |
| Brauer 2019 | x |  |
| Heath 2018 | x |  |
| Bali 2015 | x |  |
| Lee 2017 | x |  |
| Kessing 2010 | x |  |
| Kessing 2011 | x |  |
| Goveas 2012 | | x |
| Chatterjee 2015 | | x |
| Han 2020 |  | x |
| Peakman 2020 | | x |
| Su 2020 |  | x |
| Lee 2016 |  | x |
|  | 11 | 6 |
| **CCA= n-r/(rxc)-r** | 0% |  |
| N of study apperances (N) | N of rows r | N of reviews c |
| 17 | 17 | 2 |

Table 12: Androgen deprivation therapy

|  | Zhang et al 2022 | Sari Motlagh et al. 2021 | Cui et al. 2021 | Kim et al 2017 | Hinojosa-gonzalez et al 2024 |
| --- | --- | --- | --- | --- | --- |
| Baik SH, Kury FSP, McDonald CJ (2017) Risk of Alzheimer's disease among senior medicare beneficiaries treated with androgen deprivation therapy for prostate cancer. J Clin Oncol 35, 3401-3409. | x |  |  |  | x |
| Capitanio U, Isbarn H, Jeldres C et al: The use of luteinizing hormone releasing hormone agonists administrated to patients with prostate cancer predisposes to dementia: a population-based analysis. J Urol 2009; 181: 296. |  | x |  |  |  |
| Chung SD, Lin HC, Tsai MC, Kao LT, Huang CY, Chen KC. Androgen deprivation therapy did not increase the risk of Alzheimer’s and Parkinson’s disease in patients with prostate cancer. Andrology. 2016;4:481–5. |  |  | x | x |  |
| Deka R, Simpson DR, Bryant AK et al: Association of androgen deprivation therapy with dementia in men with prostate cancer who receive definitive radiation therapy. JAMA Oncol 2018; 4: 1616. |  | x |  |  | x |
| Hong JH, Huang CY, Chang CH, Muo CH, Jaw FS, Lu YC, et al. Different androgen deprivation therapies might have a differential impact on cognition—an analysis from a population-based study using time-dependent exposure model. Cancer Epidemiol. 2020; 64:101657. |  |  | x |  | x |
| Huang WK, Liu CH, Pang ST, Liu JR, Chang JW, Liaw CC, Hsu CL, Lin YC, See LC (2020) Type of androgen deprivation therapy and risk of dementia among patients with prostate cancer in Taiwan. JAMA Netw Open 3, e2015189. | x |  |  |  | x |
| Jayadevappa R, Chhatre S, Malkowicz SB, Parikh RB, Guzzo T, Wein AJ (2019) Association between androgen deprivation therapy use and diagnosis of dementia in men with prostate cancer. JAMA Netw Open 2, e196562. | x |  | x |  | x |
| Jhan JH, Yang YH, Chang YH, Guu SJ, Tsai CC (2017) Hormone therapy for prostate cancer increases the risk of Alzheimer's disease: a nationwide 4-year longitudinal cohort study. Aging Male 20, 33-38. | x |  | x | x |  |
| Kang J, Shin DW, Han K, Park SH, Lee WG, Yoo JE, et al. Risk of dementia in prostate cancer survivors: A nationwide cohort study in Korea. Curr Probl Cancer. 2020;44:100578. |  |  |  |  | x |
| Kao LT, Lin HC, Chung SD et al: No increased risk of dementia in patients receiving androgen deprivation therapy for prostate cancer: a 5-year follow-up study. Asian J Androl 2017; 19: 414. |  | x | x | x | x |
| Khosrow-Khavar F, Rej S, Yin H, Aprikian A, Azoulay L (2017) Androgen deprivation therapy and the risk of dementia in patients with prostate cancer. J Clin Oncol 35, 201-207. | x | x | x | x | x |
| Kim JW, Kim DK, Lee HS, Park JY, Ahn HK, Ha JS, et al. Androgen Deprivation Therapy in Patients with Prostate Cancer is Associated with the Risk of Subsequent Alzheimer’s Disease but Not with Vascular Dementia. World J Mens Health. 2021;40:481. |  |  |  |  | x |
| Kim YA, Kim SH, Joung JY, Yang MS, Back JH, Kim SH. The Insignificant Correlation between Androgen Deprivation Therapy and Incidence of Dementia Using an Extension Survival Cox Hazard Model and Propensity-Score Matching Analysis in a Retrospective, Population-Based Prostate Cancer Registry. Cancers. 2022;14:2705. |  |  |  |  | x |
| Krasnova A, Epstein M, Marchese M et al: Risk of dementia following androgen deprivation therapy for treatment of prostate cancer. Prostate Cancer Prostatic Dis 2020; 23: 410. |  | x | x |  | x |
| Liu JM, Shen CY, Lau WCY, Shao SC, Man KKC, Hsu RJ, et al. Association between Androgen Deprivation Therapy and Risk of Dementia in Men with Prostate Cancer. Cancers. 2021;13:3861. |  |  |  |  | x |
| Lonergan PE, Washington SL, Cowan JE, Zhao S, Broering JM, Cooperberg MR, et al. Androgen Deprivation Therapy and the Risk of Dementia after Treatment for Prostate Cancer. J Urol. 2022;207:832–40. |  |  |  |  | x |
| Nead KT, Gaskin G, Chester C, Swisher-McClure S, Dudley JT, Leeper NJ, Shah NH (2016) Androgen deprivation therapy and future Alzheimer's disease risk. J Clin Oncol 34, 566-571. | x |  |  | x | x |
| Nead KT, Gaskin G, Chester C, Swisher-McClure S, Dudley JT, Leeper NJ, Shah NH. Influence of age on androgen deprivation therapy-associated Alzheimer’s disease. Sci Rep. 2016; 6:35695. |  |  |  | x |  |
| Nead KT, Gaskin G, Chester C et al: Association between androgen deprivation therapy and risk of dementia. JAMA Oncol 2017; 3: 49. |  | x | x | x |  |
| Ng HS, Koczwara B, Roder D, Vitry A. Development of comorbidities in men with prostate cancer treated with androgen deprivation therapy: an Australian population-based cohort study. Prostate Cancer Prostatic Dis. 2018;21:403–10. |  |  | x |  |  |
| Nguyen C, Lairson DR, Swartz MD et al: Risks of major long-term side effects associated with androgen-deprivation therapy in men with prostate cancer. Pharmacotherapy 2018; 38: 999. |  | x | x |  | x |
| Robinson D, Garmo H, Van Hemelrijck M, Damber JE, Bratt O, Holmberg L, et al. Androgen deprivation therapy for prostate cancer and risk of dementia. BJU Int. 2019;124:87–92. |  |  |  |  | x |
| Shim M, Bang WJ, Oh CY, Lee YS, Jeon SS, Ahn H, et al. Risk of dementia and Parkinson’s disease in patients treated with androgen deprivation therapy using gonadotropin-releasing hormone agonist for prostate cancer: A nationwide population-based cohort study. Forloni G, editor. PLoS One. 2020;15:e0244660. |  |  |  |  | x |
| Tae BS, Jeon BJ, Shin SH, Choi H, Bae JH, Park JY (2019) Correlation of androgen deprivation therapy with cognitive dysfunction in patients with prostate cancer: a nationwide population-based study using the National Health Insurance Service Database. Cancer Res Treat 51, 593-602. | x | x |  |  | x |
| Tully KH, Nguyen DD, Herzog P, Jin G, Noldus J, Nguyen PL, Kibel AS, Sun M, McGregor B, Basaria S, Trinh QD (2021) Risk of dementia and depression in young and middle-aged men presenting with nonmetastatic prostate cancer treated with androgen deprivation therapy. Eur Urol Oncol 4, 66-72. | x | x | x |  | x |
|  | 8 | 9 | 11 | 7 | 19 |
|  | **CCA= n-r/(rxc)-r** | | 29% |  |  |
|  | N of study apperances (N) | N of rows r | N of reviews c | |  |
|  | 54 | 25 | 5 |  |  |
